# Supplementary material for: Usability, occupational performance and satisfaction evaluation of a smart environment controlled by infrared oculography by people with severe motor disabilities
Source: PLoS One. 2021 Aug 13;16(8):e0256062. doi: 10.1371/journal.pone.0256062 (PMC8362986; doi:10.1371/journal.pone.0256062)

PLOS ONE

Usability, occupational performance and satisfaction evaluation of a smart environment controlled by infrared oculography by people with severe motor disabilities

--Manuscript Draft--

**Manuscript Number:**	PONE-D-20-12562
**Article Type:**	Research Article
**Full Title:**	Usability, occupational performance and satisfaction evaluation of a smart environment controlled by infrared oculography by people with severe motor disabilities
**Short Title:**	Evaluation of a smart environment controlled by infrared oculography
**Corresponding Author:**	Mariana Midori Sime, Ph.D.<br>Federal University of Espirito Santo<br>Vitória, Espirito Santo BRAZIL
**Keywords:**	smart environment; smart home; people with disabilities; assistive technology; infrared oculography; effectiveness
**Abstract:**	<p class="MDPI17abstract" style="margin-top:0cm;line-height:200%">A smart environment is an assistive technology device that can enable people with motor disabilities to control its equipment (TV, radio, fan, etc.) through a human-machine interface activated by different inputs. However, assistive technology resources are not always considered useful, reaching quite high abandonment rates. This study aims to evaluate the effectiveness of a smart environment controlled through infrared oculography by people with severe motor disabilities. The study sample was composed of six volunteers. Initially, sociodemographic data forms, the Functional Independence Measure (FIM^{TM}) and the Canadian Occupational Performance Measure (COPM) were applied. The volunteers used the system in their domestic environment for a week. Afterwards, they were reevaluated with regards to occupational performance (COPM), satisfaction with the use of the assistive technology resource (QUEST 2.0), psychosocial impact (PIADS) and usability of the system (SUS), as well as through semi-structured interviews for suggestions or complaints. Having ‘control of the TV’ was the common demand, in which four of the six volunteers presented positive results in all assessment protocols, reaching greater independence in the control of the smart environment equipment. Non-acceptance of disability and lack of social support may have influenced the participants who showed negative results in using the system correctly. <o:p></o:p></p>
**Order of Authors:**	Mariana Midori Sime, Ph.D.<br>Alexandre Bissoli<br>Daniel Lavino-Júnior<br>Teodiano Bastos-Filho
**Additional Information:**	
**Question**	**Response**
**Financial Disclosure**<br><br>Enter a financial disclosure statement that describes the sources of funding for the work included in this submission. Review the submission guidelines for detailed requirements. View published research articles from *PLOS ONE* for specific	AB and TBF received a award in Google Research Awards for Latin America, of Google Inc.<br>The funders had no role in study design, data collection and analysis, decision to publish, or preparation of the manuscript.

examples.<br><br>This statement is required for submission and **will appear in the published article** if the submission is accepted. Please make sure it is accurate.<br><br>**Unfunded studies**<br>Enter: *The author(s) received no specific funding for this work.*<br><br>**Funded studies**<br>Enter a statement with the following details:<br>• Initials of the authors who received each award<br>• Grant numbers awarded to each author<br>• The full name of each funder<br>• URL of each funder website<br>• Did the sponsors or funders play any role in the study design, data collection and analysis, decision to publish, or preparation of the manuscript?<br>• **NO** - Include this sentence at the end of your statement: *The funders had no role in study design, data collection and analysis, decision to publish, or preparation of the manuscript.*<br>• **YES** - Specify the role(s) played.<br><br>* typeset	
**Competing Interests**<br><br>Use the instructions below to enter a competing interest statement for this submission. On behalf of all authors, disclose any competing interests that could be perceived to bias this work—acknowledging all financial support and any other relevant financial or non-financial competing interests.<br><br>This statement **will appear in the published article** if the submission is accepted. Please make sure it is accurate. View published research articles from *PLOS ONE* for specific examples.	The authors have declared that no competing interests exist.

**NO authors have competing interests**<br><br>Enter: *The authors have declared that no competing interests exist.*<br><br>**Authors with competing interests**<br><br>Enter competing interest details beginning with this statement:<br><br>*I have read the journal's policy and the authors of this manuscript have the following competing interests: [insert competing interests here]*<br><br>* typeset	
**Ethics Statement**<br><br>Enter an ethics statement for this submission. This statement is required if the study involved:<br><br>• Human participants<br>• Human specimens or tissue<br>• Vertebrate animals or cephalopods<br>• Vertebrate embryos or tissues<br>• Field research<br><br>Write "N/A" if the submission does not require an ethics statement.<br><br>General guidance is provided below. Consult the submission guidelines for detailed instructions. **Make sure that all information entered here is included in the Methods section of the manuscript.**	This study was approved by the Human Research Ethics Committee of Federal University of Espirito Santo (UFES), Brazil, under protocol no. 976.828, CAAE 39410614.6.0000.5060. All participants who agreed to participate in the research signed a Free and Informed Consent Form (FICF) and received a copy of it.

**Format for specific study types**<br><br>**Human Subject Research (involving human participants and/or tissue)**<br>• Give the name of the institutional review board or ethics committee that approved the study<br>• Include the approval number and/or a statement indicating approval of this research<br>• Indicate the form of consent obtained (written/oral) or the reason that consent was not obtained (e.g. the data were analyzed anonymously)<br><br>**Animal Research (involving vertebrate animals, embryos or tissues)**<br>• Provide the name of the Institutional Animal Care and Use Committee (IACUC) or other relevant ethics board that reviewed the study protocol, and indicate whether they approved this research or granted a formal waiver of ethical approval<br>• Include an approval number if one was obtained<br>• If the study involved *non-human primates*, add *additional details* about animal welfare and steps taken to ameliorate suffering<br>• If anesthesia, euthanasia, or any kind of animal sacrifice is part of the study, include briefly which substances and/or methods were applied<br><br>**Field Research**<br><br>Include the following details if this study involves the collection of plant, animal, or other materials from a natural setting:<br>• Field permit number<br>• Name of the institution or relevant body that granted permission	
**Data Availability**<br><br>Authors are required to make all data underlying the findings described fully available, without restriction, and from the time of publication. PLOS allows rare exceptions to address legal and ethical concerns. See the PLOS Data Policy and FAQ for detailed information.	No - some restrictions will apply

A Data Availability Statement describing where the data can be found is required at submission. Your answers to this question constitute the Data Availability Statement and **will be published in the article**, if accepted.<br><br>**Important:** Stating 'data available on request from the author' is not sufficient. If your data are only available upon request, select 'No' for the first question and explain your exceptional situation in the text box.<br><br>Do the authors confirm that all data underlying the findings described in their manuscript are fully available without restriction?	
**Describe where the data may be found in full sentences. If you are copying our sample text, replace any instances of XXX with the appropriate details.**<br><br>• If the data are **held or will be held in a public repository**, include URLs, accession numbers or DOIs. If this information will only be available after acceptance, indicate this by ticking the box below. For example: *All XXX files are available from the XXX database (accession number(s) XXX, XXX.*).<br>• If the data are all contained **within the manuscript and/or Supporting Information files**, enter the following: *All relevant data are within the manuscript and its Supporting Information files.*<br>• If neither of these applies but you are able to provide **details of access elsewhere**, with or without limitations, please do so. For example:<br><br>*Data cannot be shared publicly because of [XXX]. Data are available from the XXX Institutional Data Access / Ethics Committee (contact via XXX) for researchers who meet the criteria for access to confidential data.*<br><br>*The data underlying the results presented in the study are available from (include the name of the third party*	Data cannot be shared publicly because of Ethics Committe[illegible]rms.

and contact information or URL).<br>• This text is appropriate if the data are owned by a third party and authors do not have permission to share the data.<br><br>* typeset	
Additional data availability information:	

Powered by Editorial Manager® and ProduXion Manager® from Aries Systems Corporation

Manuscript Click here to download Manuscript Manuscript - Usability.doc

Usability, occupational performance and satisfaction evaluation of a smart environment controlled by infrared oculography by people with severe motor disabilities

Mariana Sime[1*¶,#a], Alexandre Bissoli[2&], Daniel Lavino-Júnior[3&], Teodiano Bastos-Filho[4¶,#b]

[1] Occupational Therapy Department, Federal University of Espirito Santo (UFES), Vitoria-ES, Brazil

[2] Postgraduate Program in Electrical Engineering, Electrical Engineering Department, Federal University of Espirito Santo (UFES), Vitoria-ES, Brazil

[3] Electrical Engineering Department, Federal University of Espirito Santo (UFES), Vitoria-ES, Brazil

[4] Postgraduate Program in Electrical Engineering, Electrical Engineering Department and Postgraduate Program in Biotechnology, Federal University of Espirito Santo (UFES), Vitoria-ES, Brazil

[#a] Current Address: Department of Occupational Therapy, Federal University of Espirito Santo – Maruípe Campus, Vitória, Espirito Santo, Brazil.

[#b] Current Address: Department of Electrical Engineering, Federal University of Espirito Santo – Goiabeiras Campus, Vitória, Espirito Santo, Brazil.

* Corresponding author

E-mail: mariana.sime@ufes.br (MS)

[¶] These authors contributed equally to this work.

[&] These authors also contributed equally to this work.

Abstract

A smart environment is an assistive technology device that can enable people with motor disabilities to control its equipment (TV, radio, fan, etc.) through a human-machine interface activated by different inputs. However, assistive technology resources are not always considered useful, reaching quite high abandonment rates. This study aims to evaluate the effectiveness of a smart environment controlled through infrared oculography by people with severe motor disabilities. The study sample was composed of six volunteers. Initially, sociodemographic data forms, the Functional Independence Measure (FIM™) and the Canadian Occupational Performance Measure (COPM) were applied. The volunteers used the system in their domestic environment for a week. Afterwards, they were reevaluated with regards to occupational performance (COPM), satisfaction with the use of the assistive technology resource (QUEST 2.0), psychosocial impact (PIADS) and usability of the system (SUS), as well as through semi-structured interviews for suggestions or complaints. Having 'control of the TV' was the common demand, in which four of the six volunteers presented positive results in all assessment protocols, reaching greater independence in the control of the smart environment equipment. Non-acceptance of disability and lack of social support may have influenced the participants who showed negative results in using the system correctly.

Keywords: smart environment; smart home; people with disabilities; assistive technology; infrared oculography; effectiveness

Introduction

A Smart Environment (SE) is a space (room, house, office, etc.) in which the services (temperature, lighting, communication, entertainment, security, etc.) and/or equipment (heating, air conditioning, lamps, radio, television, alarm, etc.) are managed intelligently using technology (computer, tablet, smartphone, remote control, etc.), aiming to assist users or residents with their daily activities and providing them with better quality of life (QoL) [1,2].

Smart environments, or smart homes when referring to people's homes, can also be focused on monitoring residents (older people or people with disabilities). In these cases, the SE learns the normal patterns of habits and behaviors, thus recognizing abnormal situations such as falls, prolonged time in the same place or position, repeated walking between two rooms, and changes in routine [2–6].

However, more recently, many studies have focused on the development of smart homes that aim ~~at~~ to provid~~ing~~e greater independence for people with motor disabilities, combining their residual skills with the physical environment, since this group ~~presents~~ experiences several limitations f~~or accessibl~~ in the use of e use of environments and equipment control [1,7–13].

Despite the gradual increase in the number of these studies, only few of them have addressed the benefits of SEs for people with disabilities with regard to the exercise of autonomy, i.e., freedom of opinion, choice and decision [14], improving performance and usability.

The reviews by Martin et al. [15] and Brandt et al. [16] found no evidence about the effectiveness of smart homes for people with disabilities. Differences in sample size and interventions and instruments used hinder comparison between studies, but it was possible to notice a tendency to facilitate independence, instrumental Activities of Daily Living (ADL), socialization, and quality of life.

Marikyan, Papagiannidis and Alamanos [17] consider that there is increased research addressing SEs; however, they criticize the fact that the studies are restricted to three themes: they do not consider the multiple diversities of the smart home concept; focus on the functioning of technological devices, architecture and infrastructure; present possible benefits of technology, but are little dedicated to pointing out the perspective of users.

In this sense, further studies addressing the effectiveness of SEs in the everyday life of people with severe motor disabilities should be carried out. The home environment interaction system used in this study was developed at the Assistive Technology Center (NTA) of the Federal University of Espirito Santo (UFES), Brazil. It consists of a smart global box (gBox) coupled to a computer software that enables the user to control electronic equipment (TV, radio, and fan and lighting using eye-tracking technology [18].

The main objective of this study was to evaluate the effectiveness of the developed SE controlled through infrared oculography for specific use by people with severe motor disabilities.

The study participants were assessed with regards to their occupational performance and satisfaction with the use of the Assistive Technology (AT) device, the psychosocial impact of using the environment control system, the usability of the equipment, as well as to their suggestions and complaints about the system.

The work is organized as follows: in addition to this introduction, a literature review on people with disabilities, AT, and SE is presented in second section, a description of the materials and methods used can be found in third section, the results and discussion are presented in fourth section, the fifth section contains the study conclusions, and sixth section presents details of the submitted patent.

Literature Review

People with disabilities

Prevalence of people with disabilities has increased worldwide over the years. According to the World Report on Disability [19], there is a progressive concern with this theme due to the aging of populations and the consequent increased risk of disabilities and incidence of chronic diseases such as diabetes, cardiovascular conditions, and cancer.

In adults or older people, motor disabilities often result from diseases or injuries that affect the Central Nervous System and/or Peripheral Nervous System, such as Muscular Dystrophies, Multiple Sclerosis, Amyotrophic Lateral Sclerosis, Stroke, Cranioencephalic Trauma, Spinal Trauma, among others.

The literature indicates that individuals with the aforementioned health conditions present sensory, motor, language and behavioral impairments at different levels, which lead to deficits in their occupational performance for carrying out ADL independently, as well as deficits in the interaction with people and objects [20–29], and these individuals can become quite dependent on family members and/or caregivers [30].

The aforementioned studies demonstrate how much these handicaps or disabilities impair various aspects of these people's everyday lives, decreasing their QoL, changing their occupational roles, or increasing their dependence to perform ADL. Thus, it is necessary to address this problem since many diseases or traumas affect young and/or working age populations. In this context, AT devices can be used in order to alleviate disability in these populations.

Aiming at better understanding the consequences of these diseases and aging processes, the World Health Organization (WHO) published, in 2001, the International Classification of Functioning, Disability and Health (ICF) with the general objective of

establishing a common and standardized language between different countries, as well as structuring and organizing information related to human functionality [31].

Thus, the ICF understands that a person's functionality and disability are the result of the dynamic interaction between the following elements: Health Condition, Body Functions and Structures, Activity, Participation, and Contextual Factors (Environmental and Personal). The focus is no longer centered on the disease or disorder, and people's disabilities are configured as their environments/contexts limit their activities and restrict their social participation, not favoring their functionality [31–33].

Assistive Technology (AT)

Assistive technology has been defined in ifferent ways: the area of knowledge that includes products, resources, methodologies, strategies and services [34]; items, products and equipment acquired, adapted or modified [35] with the aim of improving the functional performance, independence, and QoL of people with disabilities.

According to the ICF, AT devices and resources are allocated into the Environmental Factors of this classification, and are very important for people with disabilities due to the expansion of forms of communication, mobility, environment control, integration with networks, and social and learning skills [31,36].

Assistive technology resources can be classified or categorized according to 1) their purpose – adaptations for ADL, Alternative and Augmentative Communication (AAC), accessibility for computer use, etc. [36]; 2) their complexity – low-tech (easier to acquire, control or build, not requiring electricity) or high-tech (more difficult to acquire, more expensive and complex, including electronic components, or being electronically powered); 3) ranging from hard technology (palpable, whose components can be

purchased and/or assembled, such as computer hardware, AAC, or mobility devices) to soft technology (less palpable, with support for the use of devices, such as knowledge, strategies, training, services and software) [37].

Therefore, knowledge of several professionals from different areas may be involved in the prescription, development, manufacturing and application of AT, such as engineers, designers, physiotherapists, speech therapists, physicians, occupational therapists, prosthetists, among others [36–38].

The literature indicates that AT resources provide countless benefits, of both low and high technology, to people with motor disabilities and/or older people, especially in the area of education, work, and life in the community [39]; to the functional performance of ADL, psychosocial aspects, and reduction of expenses with caregivers and hospital admissions [40–42]; to the communication and psychosocial adjustments of people with Incarceration Syndrome [43,44] or degenerative diseases [45]; to independence, social participation and reduced caregiver burden [46,47]; to improving the function of upper limbs [21].

In this context, SE[illegible]s can be indicated for people with severe motor disabilities, as they enable even individuals with little mobility to control the lighting and electronic equipment of their environment (TV, radio, fan) through a Human-Machine Interface (HMI) configured to be triggered by different inputs such as voice; surface electromyography: touch, head movements, inspiration and expiration (sip-n-puff), eye blink; electroencephalography: brain waves; infrared oculography: eye tracking.

However, it is known that although AT plays an important role in the recovery or improvement of the functionality of people with disabilities, clinical practice and the literature show high rates of abandonment or non-use of AT devices by users [48–51]. In the same sense, the literature also points to a lack of studies that address the effectiveness

provided by AT in this population [52,53], as well as the importance of good assessment using valid, reliable and viable instruments, and covering various aspects of AT [54].

Several studies have been conducted with the aim of understanding the abandonment or non-use of AT devices. Many reasons have been described: people do not like or need the device anymore, stigma and shame for the need of AT, highlight of the need for help, cognitive problems, cultural or traditional reasons, unfamiliarity of its advantages, because the device is chosen only by the therapist or the family (not by themselves), low esthetic quality, difficulty in maintaining and programming it, and difficulty in performing the desired activities [42,47,48,55].

With the aim of decreasing AT abandonment and dissatisfaction rates and increasing the sense of independence, the literature highlights the importance of patient/user participation in the development of the AT resource or device [46,56–58], or in the process of defining and choosing the device best suited to their needs. It is also important that a trained and updated team carry out the evaluation and monitoring of AT use [46,48,49,59].

Regarding SEs, it is necessary to assess the individuals' real demands in controlling the environment, the inputs that can be used, the expectations and factors involved in the process of acceptance and use of this resource, as well as understanding the benefits that this technology can bring to their occupational performance and use satisfaction.

Zhao, Zhang and Crabtree [60] highlight that HMI design plays an important role in the development and improvement of SEs, as well as advances in the technology of sensors, monitors and controllers, which provide the necessary tools for their more efficient and effective use.

Thus, for the indication and implementation of AT devices, a possible start would be from the study of several conceptual models, from which the Human Activity Assistive

Technology (HAAT) model [37,61] was used in this study. The HAAT model proposes to understand the role of AT in the lives of people with disabilities, and is based on four elements: the Human, the Activity, the Assistive Technology, and the Context in which the other three elements are inserted. Thus, the HAAT model describes “someone (human) doing something (activity) in a context using assistive technology” [37] (p.7).

According to Cook and Polgar [37], the Activity is the central element of the model, as it defines the objective of using the AT resource, directed to the person's needs. It also includes temporal aspects (duration and frequency of participation), involvement of other people, and place where it occurs. The Human component includes motor, sensory, cognitive and affective skills, as well as the roles that the person plays in life (a combination of the many activities that characterize their identity), their experience with technology, and their motivation to resume the performance of activities and for the use of technology [37]. The Context considers the physical (natural and built environments), social (people, society, attitudes), cultural (beliefs, rituals, values, being part of a group) and institutional (laws, regulations, policies, funding that enable social inclusion) aspects [37]. Finally, the Assistive Technology factor is the facilitator, so that the person can perform a certain activity in the context in which they are inserted [37].

Thus, during the process of preparing and/or indicating an AT resource or device, it is important to understand the activity that the person wants and needs to perform, the capacities they have, and the influence of different aspects of the context that will influence their acquisition and use.

In this study, the person with motor disabilities is considered as the Human component; the Activity is the use and control of electronic equipment; the AT component is the SE, controlled by eye tracking; the Context refers to higher autonomy and independence within the domestic environment.

Smart Environment (SE)

According to Rampinelli et al. [62], an SE consists of a network of sensors used to obtain information from the observed world, together with a network of actuators (computer screens, wheelchairs, equipment, devices, etc.) that enable users to interact. For those authors, the sensor network and the intelligence that regulates it are invisible to the user, making decisions and cooperating between different autonomous agents in order to carry out an action or task.

Lee and Hashimoto [63] present SE as a space where distributed sensors, connected to a network, observe people and provide them with information, in addition to controlling all systems through actuators. This space includes robots, which are assisted by the SE, working as physical agents and performing services for people.

According to Aarts and Encarnação [64], in the term Ambient Intelligence, Ambience refers to the environment and reflects the need for a strong integration of technology with everyday objects, whereas Intelligence refers to how the environment should be able to recognize the people who live in it, adapt to them, learn from their behavior, and possibly act on their behalf.

Therefore, the objective of an SE is to improve “the quality of people’s lives by creating the desired atmosphere and providing appropriate functionality by means of intelligent, personalized and interconnected systems and services” [64] (p.2).

Aldrich [65] proposes five hierarchical classes of smart homes, complemented by Gentry [13], based on the functionality available to the user: Class 1 is made up of homes that contain intelligent stand-alone objects: simple and autonomous appliances and objects that work intelligently by remote control, touch, sip-n-puff, voice control and eye gaze command, and contain light sensors, smoke and water leak detectors, alarm clocks and medication alarms. Class 2 comprises homes that contain intelligent and

communicating objects that use wired and wireless networks to exchange information with each other, such as devices controlled by computers; they usually connect Class 1 devices to computers, making it possible to schedule sequential activities. Class 3 includes connected homes that have internal and external networks, enabling remote control of equipment, via internet, cameras or telephone; they connect class 2 systems to external computers. Class 4 is composed of learning homes in which computers register the patterns of human activities and administer the devices according to the residents' needs. Class 5 includes attentive homes, which are also based on learning the patterns of human activities to control technology in anticipation of the residents' needs.

According to Gentry [13], classes 1 and 2 are composed of passive systems that cannot monitor the performance of residents, unlike those in classes 3, 4 and 5.

Lee and Hashimoto [63] claim that in an SE, the architecture of software and hardware must present six properties: 1) be modular (components can be added or removed from the environment); 2) be scalable (enable integration of local spaces with larger systems); 3) present integration (be simple and integrated with intelligent components or services existing in the space); 4) be achievable (technologies and elements that can be easily obtained); 5) be inexpensive (due to the number of components required and use of standard hardware); 6) be of easy setup and maintenance (automatic calibration and easy adaptation to the environment).

Zhao, Zhang and Crabtree [60] discuss approaches that they consider to be innovative and that provide general guidance to assist designers and developers with improving user experience and satisfaction with the SE, such as having more efficient general control panels (familiar to users), effective user interfaces (known by users and with individualized settings) and variable accessibility (setting limits for children), as well as securing privacy. In contrast, Gentry [13] reports that smart home technology was

initially developed to provide comfort for people and that, over time, has been used to assist people with mobility, sensory or cognitive problems. According to that author, smart homes for people with disabilities refer to the use of electronic AT resources aiming at increasing their independence and reducing their need for help from caregivers or family members.

For the control of electronic equipment in a smart home by people with motor disabilities, different ways of capturing their inputs can be used, and Infrared Oculography (IROG) is a technique that has been significantly studied in computer science [10,13,18,66,67].

In IROG, a device performs eye movement tracking, calculating the point on the computer screen the user is looking at (a technique known as eye tracking). Eye tracking devices have a video camera equipped with high resolution infrared (IR) light-emitting diodes (LED) that reflect and increase the contrast between the pupil and the iris, allowing precise pupil location and facilitating the tracking of eye movement. This movement then functions as an HMI modality, enabling users to control several applications through eye tracking [68–70].

This technique has proved to be one of the most indicated and useful for people with severe motor disabilities who are unable to hold or manipulate a computer keyboard or mouse, making them able to use this HMI in an easier, comfortable and intuitive way [71], without the need to place electrodes or equipment on their bodies. Another factor that contributes to the use of IROG is that eye movement is one of the few abilities maintained in people with severe motor disabilities [72].

Materials and Methods

This study was approved by the Human Research Ethics Committee of Federal University of Espirito Santo (UFES), Brazil, under protocol no. 976.828, CAAE 39410614.6.0000.5060. All participants who agreed to participate in the research signed a Free and Informed Consent Form (FICF) and received a copy of it.

Materials

The following materials were used in this study:

Notebook computer: HP 14-AP020, with Intel® Core™ i3-5005U processor, Windows® 10 Home Edition operating system, 4GB RAM and 500GB HD memory, integrated graphics with Intel® HD graphics technology, and 14” LED screen. Tobii Eye Tracker 4C [73]: the advantages of the Tobii 4C for this study are as follows: a) possibility of making movements with the head without changing the equipment calibration, b) possibility to choose whether the control will be carried out with only one or both eyes, c) booting with the computer, and d) register of the user with no need for frequent calibrations. For better use, the Gaze Point software [74], which allows controlling the mouse cursor, was also installed in the notebook computer. Global Box (gBox): an SE controller module [18]. Through the computer, the user sends commands to the gBox (Fig 1) via Wi-Fi to activate the home devices, which are attached to the box sockets. SE control interface (CI) (Fig 2) [18]: configured in a Web application with a management center in which it is possible to view the last commands emitted by the user, configure the signals of the TV remote control(s), record the actions performed by the user, download the data history, and change access passwords. The CI covers the whole notebook computer screen, allowing greater concentration during use. When clicking on START in the initial interface (a), a CI containing the main menu (b)

appears on the screen, and the user can go to the devices they wish to turn on (START), configure the system (CONFIG), or return to the initial screen (CLOSE). In the configuration menu (c), the user can choose the size of the icons (small, medium, or large) and the reaction time of the cursor (0.5s, 1.0s, 2.0s, or 3.0s). In the home equipment control menu (d), the devices registered for control (fan, radio, TV, and lighting) are displayed. To activate the device, the mouse cursor must be positioned on its icon for the time defined in the settings. When activated, the background of the icon turns yellow (e), except for the TV icon, which when activated directs the user to a submenu (f) to turn it on/off or control its channels or volume. In this submenu, when any icon is clicked on, it will momentarily show a yellow background, indicating the click. Router: D-Link Wi-Fi Router + 150Mbps repeater mode: it has the function of sending equipment control commands from the notebook computer to the gBox. Portable table: used to position the equipment (Fig 3), facilitating its transport and use by the user, occupying the smallest space possible in the residence.

Fig 1. gBox: electronic module to control home devices in the SE.

Fig 2. User control interface. Reproduced with permission from [18].

Fig 3. Portable table, eye tracker, and notebook computer are installed.

Data collection instruments

The following instruments were used to collect information from the volunteers during HMI use:

Sociodemographic data forms applied at the beginning of the evaluation to collect the participant's personal data (name, age, gender, occupation, primary caregiver, education level, contacts, etc.), as well as information on the diagnosis and history of the disease or injury, body structures affected, main complaints, treatments undergone, and experience with the use of technology.

Functional Independence Measure (FIM^{TM}) [75,76]: it assesses the degree of assistance needed so that users can perform motor and cognitive activities. Eighteen tasks are evaluated, divided into self-care, sphincter control, transfers, locomotion, communication, and social cognition. The score ranges from 1 (total assistance) to 7 (complete independence). In this study, this measure was applied to characterize the difficulties of each volunteer in the performance of ADL, and was scored based on the volunteers or caregivers' reports.

Canadian Occupational Performance Measure (COPM) [77]: created for the practice of Client-centered Occupational Therapy, it evaluates the intervention results, scoring from 1 to 10 the changes in the client's perception of their performance in carrying out activities and satisfaction with performance. Changes are considered significant when two or more points are reached between the initial and final assessments. It was directed to obtain information on demands for the use of home devices in SEs. In this study, it was applied at evaluation and reevaluation.

Psychosocial Impact of Assistive Devices Scale (PIADS) [78]: it assesses the effects of an AT device on the functional independence, well-being and QoL of users. It is self-administered, punctuating the 26 sentences in the questionnaire using a Likert scale that varies between -3 (maximum negative impact) and +3 (maximum positive impact). This instrument is divided into three subscales: Competence (impact of the device on functional independence, performance and productivity), Adaptability

(feeling of willingness to risk, how much the device enables participation in activities), and Self-esteem (effect of the device on self-confidence, self-esteem, and emotional well-being), which according to the developers are fundamental for QoL. This scale was applied at reevaluation.

Quebec User Evaluation of Satisfaction with Assistive Technology (QUEST 2.0) [79,80]: it assesses satisfaction with the AT resource (eight items) and with the services received by the user (four items) from a scale ranging from 1 (not fully satisfied) to 5 (very satisfied). In its second part, the interviewees must indicate three out of the 12 items that they consider most important. This instrument aimed to assess the satisfaction with the environment control system and the services offered by the team with regard mainly to guidelines, equipment installation, and training for use. It was applied at reevaluation.

System Usability Scale (SUS) [81]: it is used to evaluate the usability of the environment control system. It is self-administered, with ten sentences covering a variety of aspects of the system usability, such as need for support, training and complexity, with a high level of apparent validity for measuring usability. It is scored using a 5-point Likert scale ranging from 1 (strongly disagree) to 5 (strongly agree). This tool was applied at reevaluation.

Semi-structured interviews applied at reevaluation with the aim of obtaining information about the process of using the system that could not be obtained from the other instruments. Issues such as time of use, positive and negative points of the system, complaints, and suggestions for improvement were addressed. For better registration and analysis, the interviews were audio recorded and later transcribed.

Participants

Inclusion criteria were as follows: people aged ≥18 years with motor disabilities that compromised the normal interaction with equipment in the home environment, such as people with quadriplegia, Amyotrophic Lateral Sclerosis (ALS), Multiple Sclerosis (MS), among other health conditions indicated by rehabilitation institutions or professionals. Volunteers should also have a caregiver of age and literate. Individuals with cognitive deficits that compromised understanding of the equipment functioning and use, as well as of the assessment instruments, and with visual deficits not corrected by lenses (glasses or contact lenses) were excluded from the study. Information on the cognitive deficit of each volunteer was obtained from their assisting professionals.

Procedures

A visit to each of the volunteer's homes was scheduled for the initial assessment and installation of the system. After acceptance, each volunteer or caregiver signed a FICF, responded to the sociodemographic data form, and underwent application of the FIM^{TM} and COPM measures, which address activities that require the use of equipment at home.

The system was installed in the residence room most used during the day as indicated by the volunteer or caregiver. Figure 4 shows the system installed in the home of one of the volunteers.

Fig 4. System installed in the home of one of the study participants.

Both the volunteer and the caregiver were trained to use the system. The caregiver's role was to turn on the notebook computer, position the table where the computer and the eye tracker were installed, and calibrate the eye tracker whenever necessary. A copy of

the user manual containing detailed explanations on the eye tracker calibration and equipment control was also provided to the participants.

The system remained installed at each participant's home for one week, as in the study conducted by Calvo et al. [82], who evaluated the impact of using an eye tracking device on the QoL of people with ALS for seven days and found improvement in the perceived QoL of users. When necessary, extra visits to the participants' homes were made in order to make adjustments or assist with use.

At the end of that period, reassessments were carried out using the COPM, the other instruments (QUEST 2.0, PIADS, and SUS), and the semi-structured interview.

It is worth mentioning that a pilot study was previously carried out with a participant with motor disabilities aiming to verify the system functionality in a common home environment, and whether the defined methodology was adequate to the study objectives with regard to application of the evaluation protocols and reassessments, verbal and written guidelines, and training. For this volunteer, specifically, the system was used for three weeks and presented no problems.

The pre- and post-test statistical analyses of the COPM instrument were performed using the Paired Sample *t*-Test considering a statistically significant difference of 5% ($p<0.05$). This statistical test was chosen because of its robustness, considering the low sampling power obtained with small samples. Results of the other instruments are presented descriptively.

Results and Discussion

Six people with disabilities participated in this study. Of these, two individuals did not use the system during the period that the equipment remained in their homes, and their cases will be discussed later.

Despite its small sample size, this research aimed to analyze few volunteers for a prolonged time of use of the system in their homes, and not occasionally in the laboratory, because its main objectives were to assess occupational performance, usability, and satisfaction with the developed AT device.

Table 1 shows the main information about the volunteers who used the system.

Table 1. Data of participants who used the system

Volunteer	Age	Gender	Education level	Caregiver	Health condition	Time elapsed since diagnosis
**PP1**	63	F[a]	Complete higher education	Husband	Amyotrophic Lateral Sclerosis + Psoriatic Arthritis	5 months
**PP3**	30	F	Incomplete higher education	Mother + formal caregiver	Autoimmune vasculitis	8 years
**PP5**	46	M[b]	Complete high school	Fiancée	Spinal cord injury (incomplete C7 level)	29 years
**PP6**	58	M	Complete higher education	Former wife + children	Amyotrophic Lateral Sclerosis	1 year and 9 months

[a] F – Female; [b] M – Male.

The mean age of participants was 49 years. Volunteers PP1, PP5, and PP6 presented basic knowledge of technologies, more focused on the use of cell phones. Volunteer PP3 had intermediate knowledge in using computers and cell phones before presenting the disease signs and symptoms.

Results on functionality collected through the FIM™ are presented in Table 2.

Table 2. FIM™ results of participants who used the system

Volunteer	Motor FIM™	Cognitive FIM™	Total FIM™
**PP1**	47/91	35/35	82/126
**PP3**	25/91	23/35	48/126
**PP5**	44/91	35/35	79/126
**PP6**	67/91	35/35	102/126

As previously described, the FIM™ score considers the need for assistance in each activity, ranging from 1 (total assistance) to 7 (complete independence). For a better understanding of the volunteers' motor condition, the following brief descriptions of each of them are presented. As for the cognitive part of the FIM™, only volunteer PP3 presented a lower score as a result of difficulty in communication.

- PP1: 63 year-old female.

  Diagnosed in June 2018 with ALS and Psoriatic Arthritis with onset of symptoms in 2016. She cannot walk long distances and can only take a few steps at home. Currently, she spends most of her time in a wheelchair or in bed. She presents a deficit in strength and dexterity in the upper limbs. She mentions that taking a shower and eating alone takes her longer than usual. She frequently uses her smartphone to communicate with friends and family, and the TV remote control with some difficulty, pressing the wrong buttons and taking longer. Her husband is her main caregiver, assisting her with the most difficult activities (getting dressed and transfers).

- PP3: 30 year-old female.

  In 2010, she started showing signs of strabismus, lethargy, and loss of balance. She was immediately assisted for accelerated loss of motor skills. After investigating several illnesses unsuccessfully, she was diagnosed with autoimmune vasculitis. Currently, she is bedridden, cannot actively move her trunk and limbs, and is being fed with the use of a gastrostomy tube. She has strabismus and can control only the

movements of the left eye. She communicates only by blinking her eyes (‘yes’ or ‘no’ responses) or through slight head movements. She is totally motor dependent. The family complains mainly about her communication difficulties. When she is not undergoing rehabilitation treatments, she spends all day watching TV, which is controlled by a formal caregiver or her mother.

- PP5: 46 year-old male.

Diagnosed with spinal cord injury (C7 level incomplete) since the age of 16 after diving into the sea and hitting his spine onto a sandbar. He underwent treatment for 3 years and 6 months at our rehabilitation center. In 2017, he acquired pneumonia and, as a consequence of constant coughing, the injured vertebrae suffered consecutive impacts, resulting in spinal cord compression. In May 2018, after being diagnosed with compressive myelopathy and severe spinal canal stenosis, he underwent a surgical procedure, receiving pins in the cervical vertebrae. As he suffered the accident when he was an adolescent, he has learned to adapt to several activities. However, he only walks short distances with the aid of forearm crutches or the support of another person. He presents no dexterity in his hands and cannot make fine grips; gross grasps are made by fitting objects into his hand, whose fingers remain flexed. The activities that he needs the most assistance with are grooming, getting dressed, toileting, and use of instruments and equipment that require coordination and dexterity (knife, remote control, fan, etc.), for which he is assisted by his fiancée (main caregiver) or his daughters, who are still minors.

- PP6: 58 year-old male.

Diagnosed with ALS since April 2017 with onset of symptoms in 2015. He started with loss of strength in the left hand, reaching the entire left upper limb, followed by the right upper limb. The lower limbs are not affected. Currently, he does not

perform movements of the shoulders and elbows, remaining with the upper limbs pending. He has reduced strength in the left fingers and cannot move his right hand. His greatest difficulties regard feeding, grooming, getting dressed, and bathing. He spends much of the day alone at home watching TV. He has difficulty using the TV remote control, and often drops it when trying to handle it, or presses the wrong buttons. He keeps the fan on the floor and turns it on with his foot.

In the COPM assessment, which is directed to activities that require the use of equipment, the volunteers were asked to indicate which activities were important in their everyday lives. Having 'control of the TV' was considered important by all participants who used the system (PP1, PP3, PP5, and PP6), being able to 'turn the fan on/off' was deemed essential by two participants (PP5 and PP6), and having 'control of the lights' was a significant demand for only one of the participants (PP1).

The TV is a very popular appliance and an important resource of entertainment. Thus, the desire to improve control over this appliance by the people with disabilities that participated in this study is understandable. In the study by Myburg et al. [83], TV control systems were among the most frequent environment control devices in the population studied (people with spinal cord injury).

Although the system presents more options to control electronic devices, it was noticed that the volunteers of this study did not use all the possible equipment, according to assessments of the demand of each participant carried out using the COPM. Several researchers have reported the significance of considering factors that are important for the people who will use an AT device [46,48,58] aiming at better adherence and results.

Two volunteers (PP1 and PP6) wear glasses. Duchowski [69] points out that the use of lenses (contact lenses or glasses) can interfere with the eye tracker ability to locate the

corneal reflex, as they have reflective surfaces; however, the use of glasses did not interfere with the performance in using the system in the present study.

The way the equipment was organized, on a portable table, allowed the kit (notebook computer + eye tracker) to adapt itself to the different room configurations of the participants, with little interference in the organization and dynamic of the homes. The system was set up in the living room of three participants (PP1, PP5 and PP6) and in the bedroom of one participant (PP3).

At reassessment, volunteers PP1, PP5 and PP6 responded to the questions of the instruments with the help of the researcher to make markings on paper, and the interview was answered without help. With regard to volunteer PP3, the interview was conducted with her mother and, for the other evaluations, the scales were designed in the notebook computer and the participant was asked to move the mouse cursor with the use of the eye tracker to indicate the alternative most appropriate for each item evaluated. It was very difficult to choose the answers because the cursor position showed great variation, but the COPM was fully answered by the volunteer. The SUS and QUEST 2.0 (Brazilian version) were answered jointly with the participant's mother. Due to tiredness, her mother responded to the PIADS based on what she believed her daughter's responses would be.

Results of the COPM and the Paired Sample *t*-Test for all participants are shown in Table 3.

Table 3. COPM and Paired Sample *t*-Test results of participants who used the system

Volunteer	Demands	Performance		Satisfaction		Change	
		P1[a]	P2[b]	S1[c]	S2[d]	P2 Total - P1 Total	S2 Total - S1 Total
**PP1**	Control of the TV	7	8	5	8		
	Control of the lights	6	7	5	8		
	Total score	6.5	7.5	5	8	1	3
**PP3**	Control of the TV	1	7	1	7		
	Total score	1	7	1	7	6	6
**PP5**	Control of the TV	1	10	5	10		
	Turn the fan on/off	1	10	1	10		
	Total score	1	10	3	10	9	7
**PP6**	Control of the TV	3	9	3	10		
	Turn the fan on/off	5	9	3	10		
	Total score	4	9	3	10	5	7
p*-value		Performance		Satisfaction			
**Control of the TV**		0.045		0.009			
**COPM total score**		0.050		0.009			

[a] P1- initial performance; [b] P2- final performance; [c] S1- initial satisfaction; [d] S2- final satisfaction. * Paired Sample *t*-Test ($p<0.05$).

In the COPM, the volunteers self-evaluated their performance (P) in activities they considered important and their satisfaction (S) with performance before and after using the system using a scale from 1 to 10; the higher the score, the better the performance or satisfaction.

The final assessments (P2 or S2) of all participants who used the system were higher than their initial assessments (P1 or S1). Except for the change in performance of volunteer PP1, all other evaluations showed changes greater than two points, which is considered by Law et al. [77] as a clinically important change.

For the Paired Sample *t*-Test, only the events ‘control of the TV’ and ‘COPM total score’ were analyzed, as these events were common to all participants.

Statistically significant results were observed for performance and satisfaction regarding ‘control of the TV’ and for total satisfaction after using the system. Borderline results were obtained with respect to total performance’.

Despite the reduced sample size and the short usage time of the AT system, the results clearly showed that the use of the system provided participants with a new way of interacting with the environment more actively.

For volunteers PP3, PP5 and PP6, the initial evaluation scores indicate that they were unable to perform the activities or presented great difficulty in performing them, also reflecting on their low satisfaction with performance. At the final assessment, the scores reflect that the use of the SE control system enabled better performance and, consequently, greater satisfaction with performance for the volunteers.

Among all the participants, volunteer PP1 was the only one who still has some manual skills, thus she can operate the TV remote control, although with some difficulty, but gets tired along the process. Therefore, she presented higher initial scores and smaller changes at reevaluation.

Table 4 shows the results obtained with application of the QUEST 2.0 instrument.

Table 4. QUEST 2.0 scores

Volunteer	Resource	Service delivery	Total
**PP1**	4.5	5.0	4.7
**PP3**	4.4	5.0	4.6
**PP5**	5.0	5.0	5.0
**PP6**	5.0	5.0	5.0

The assessment related to satisfaction with the AT resource involves eight items, namely, dimensions, weight, adjustments, safety, durability, simplicity of use, comfort, and effectiveness [79]. In order to evaluate the items in this category, the volunteers were asked to take into account the entire set of hardware (gBox, notebook computer, eye tracker, router, and portable table) and software (equipment control program) installed in their residences.

Assessment of satisfaction with the service provided involves four items, namely, service delivery, repairs and servicing, professional services, and follow-up services [79], and the study participants should consider the service provided by the researchers with regard to installation of the equipment, explanations, training, troubleshooting, and necessary follow-up during the week they are using it.

Results regarding the degree of satisfaction with the SE ranged from 4.4 to the maximum score, 5.0, with scores below 5.0 referring to difficulties in calibrating the eye tracker, occasional visual discomfort, and difficulty in using the system (in the case of the volunteer with the greatest motor impairment).

With regard to satisfaction with the service, the average of all volunteers was 5.0, the highest possible score. Good professionals and services are items appointed by Lenker et al. [46] as a positive points in the process of obtaining an AT resource, and lead to better results with use. In contrast, lack of continuous support for the use of AT devices can lead participants to lose interest in their use [40].

The total average of each volunteer varied between 4.6 and 5.0, corresponding to high levels of satisfaction. This result corroborates those reported in two studies of the systematic review conducted by Brandt et al. [16] on environmental control systems and smart homes used by people with disabilities, in which the participants presented high total average scores for satisfaction with the tested systems.

Table 5 presents a distribution of the items that the study participants considered important about the SE control system. In such evaluation, each volunteer should indicate three items. It can be observed that three of the four volunteers pointed to effectiveness as an important feature of the SE tested. This term is defined by Demers et al. [84] as the "goal achievement with the AT device" (p.189), reinforcing that the system has met the needs of these people.

Table 5. Important items regarding the SE system

Item	Number of citations
**Effectiveness**	3
**Adjustment**	2
**Simplicity of use**	2
**Professional services**	2
**Follow-up services**	1
**Comfort**	1
**Safety**	1

Our findings corroborate the study by Shone Stickel et al. [85], whose participants also assessed effectiveness as the most important attribute of electronic AT devices for performance of ADL.

As for the PIADS instrument, Table 6 presents the score for each subscale and the final average of the volunteers.

Table 6. PIADS subscale scores

Volunteer	Competence	Adaptability	Self-esteem	Average
**PP1**	2.6	3.0	3.0	2.9
**PP3**	1.3	0.7	1.8	1.3
**PP5**	2.5	3.0	2.6	2.7
**PP6**	2.8	3.0	2.3	2.7

In the PIADS, respondents assessed how they were affected by the SE system in each of the 26 sentences of the scale ranging from -3.0 to +3.0, with 0 indicating that no effect was perceived. Participants PP1, PP5 and PP6 had average values close to the maximum score (3.0), indicating a maximum positive impact with the use of the SEs. They assigned the highest values to the Adaptability subscale, indicating that with the use of the system they felt more willing to take risks and more motivated to participate socially [78]. Volunteer PP3 presented the lowest average among the participants. Because she was the volunteer with the most significant motor impairment, she had more difficulty in controlling the equipment, getting tired and frustrated sometimes. In addition, this instrument, as previously mentioned, was answered by her mother based on what she believed her daughter’s assessment would be. Thus, it may not reliably represent the volunteer's assessment.

The developers of this instrument [78] claim that these three subscales are sufficiently sensitive to assess the psychosocial impact of an AT device or resource on the user, and are included in the QoL concept. The instrument development study [86] points out that the longer the period of use, the greater the feeling of competence. The developers raised two hypotheses for this result: 1) the longer the usage time, the more the users appreciate the effect; 2) the longer usage time reflects the user's real need for the device.

Due to the short time of use of the SEs in this study (one week), it is not possible to state that there was a real change in the psychosocial aspects of the participants, but it indicates a tendency toward this change, in view of the results.

Figure 5 illustrates the results of the instrument used to assess the usability of the system, the System Usability Scale (SUS).

Fig 5. SUS results of participants who used the system.

The results in Figure 5 show that the participants' average SUS score was 85.6. According to Bangor, Kortum and Miller [87], products evaluated in the range of 80 points are considered good, and products evaluated in the range of 90 points are considered exceptional. Thus, the usability of our system was well evaluated. The lowest evaluation refers, again, to participant PP3, whose answers pointed to some degree of complexity in the system and the need of a technical person or prior learning. As previously mentioned, the motor impairment of this participant interfered with the use of the eye tracker, hindering her control of the SE system. In fact, several factors may have influenced this difficulty, such as position in bed, control of only one eye, small opening of the eye sometimes, tiredness with use, and difficulty of caregivers with the use of computers and programs.

Despite the lower ratings assigned by this volunteer, it seems that for all participants, on average, the assessment instruments showed positive results in relation to occupational performance, satisfaction with performance, satisfaction with the SE system, and usability of the system.

Many studies have evaluated improvements in these aspects after people with disabilities used environment control systems or electronic AT devices [12,40,85,88,89], whereas other studies have assessed ways of interacting with the environment through eye trackers [90,91]. However, no studies with the same objectives and using the same methodology of the present research, that is, use of IROG technology for SE control, have been found for comparison.

As the results show better occupational performance, satisfaction with performance and with the SE, and system usability, it can be concluded that the SE controlled by IROG evaluated in this research provided people with motor disabilities with more independent operation and control of the equipment.

Information collected through the interviews complemented the assessment instruments used. All volunteers who used the SE system found it useful, mainly because it provided them with greater independence and exercise of autonomy in controlling the equipment, as it can be verified in some of their answers:

"Ah, it is useful in all aspects, right? Turn on, turn off" (PP1)

"I think it was good. I think (PP3) was happy to get it, right? You saw her expression of joy, right? So it was (useful). The part that I found most positive is giving autonomy, right? This is fundamental!"(PP3's mother)

"Its ... accessibility to be able to move. (...) it was very useful ... with the difficulty that I have (...). The facility for you to pick up and do things" (PP5)

"It brings independence! Not depending on anybody to 'turn up the volume!', 'Switch channels!', or 'turn on the television!', 'turn off the television!'" (PP6)

These statements corroborate the researched literature [92-95], since independence, control and privacy are highly important aspects pointed by people with disabilities who used environment control systems or electronic aids to daily living (EADL).

Volunteer PP6's speech also points to another theme present in the study conducted by Verdonck, Chard and Nolan [93]: the embarrassment that people with disabilities present regarding their recurring requests for help, followed by apologies, as they feel uncomfortable to interrupt their caregivers' routine. According to those authors, the use of

EADL changes this dynamic of the relationship, with fewer apologies, less discomfort, and reduced caregiver burden.

As examples of difficulties or aspects that need to be improved in our system, the volunteers reported the delay to switch TV channels, which are very distant from each other; feeling tired or presenting a mild headache from using the eye tracker; depending on a caregiver or family member to start the system and open the CI; complicated processes for calibrating the eye tracker.

In this sense, the following suggestions were made: having a numeric keyboard, so that the user could click on the desired channel; remove the need to use the notebook computer keyboard for some tasks, such as when typing the password to access the CI; make the system simpler and more logical for people who do not have much experience with computers. Other suggestions that emerged were allow the system to also control the fixed lighting of the residence and make and receive phone calls via a smartphone.

Regarding the user manual, volunteers PP1, PP5 and PP6 reported that they did not need to access it, because the explanation and training provided by the researcher were sufficient to use the SE system. Volunteer PP3's mother, on the other hand, reported that the manual did not clarify her doubts, requiring the presence of one of the researchers for assistance.

The literature highlights how important explanations and training are for understanding the use and for adherence to the AT resource. Myburg et al. [83] conducted interviews with people with spinal cord injury who used environment control systems and found that training was considered crucial for the total integration of the system in their lives. Participants also stressed that involvement of the occupational therapist in the test, prescription and configuration of the environment control system is equally important.

The system usage records were obtained through the Web application, which enabled verification of the number of days that each volunteer effectively used the SE (Table 7).

Table 7. Usage registration information obtained through the Web application

Volunteer	Number of days of use
**PP1**	2
**PP3**	5
**PP5**	2
**PP6**	4

As it can be observed, although the system remained in each volunteer's home for seven days, it was not used every day. The justifications given by the volunteers included trips, medical or rehabilitation consultations, and other leisure activities, such as going to church or taking short tours.

However, some other hypotheses that may have influenced the relationship between the user and the system were raised, corroborating the literature: the fact that the system has limitations, not allowing total independence and requiring a caregiver or family member to activate part of the equipment (turn on the notebook computer, correctly position the eye tracker in relation to the user and calibrate it) [94,96] or, when there is presence of some motor disability, people prefer to behave as they are more accustomed [97]. Another possibility is that, if there were a family member in the same room watching TV together with the participant, the control of the device would probably be performed by this person, using the standard TV remote control.

Table 8 shows the data of the participants who did not use the system.

Table 8. Data of volunteers who did not use the system

Volunteer	Age	Gender	Education level	Caregiver	Health Condition	Time elapsed since diagnosis
PP2	68	F[a]	Complete higher education	Formal caregivers	Multiple Sclerosis	4 years
PP4	48	M[b]	Complete high school	Mother and formal caregivers	Spinal Cord Injury (C5 level)	2 years

[a] F – Female; [b] M – Male.

At the initial assessment using the COPM, both PP2 and PP4 reported that watching TV was a very important activity in their everyday lives, but that they were not satisfied with the way they performed this activity. Thus, the TV was the only device connected to the gBox for control.

Table 9 presents the results of the FIM™ with respect to functionality.

Table 9. FIM™ results of the participants who did not use the system

Participant	Motor FIM™	Cognitive FIM™	Total FIM™
**PP2**	26/91	35/35	61/126
**PP4**	13/91	35/35	48/126

According to the FIM™ data, both volunteers (PP2 and PP4) had need for maximum assistance to perform motor activities and presented total independence for cognitive activities. A brief description of each of these participants is presented ahead:

- PP2: 68 year-old female.

Diagnosed with MS since 2008 with onset of symptoms in 2004, presenting numbness in the limbs and optic neuritis. She used to live in another state and moved after requests by her children for diagnostic investigation and treatment. She

spends most of the day in her bedroom, sitting in the armchair and watching TV. She is dependent regarding all ADL and does not walk alone. She is able to perform some movements with her upper limbs, but with little amplitude and strength, and handles the smartphone and the TV remote control with difficulty. She complains mainly about lack of independence and always needing to ask for help. She has two married children, but lives in an apartment only with formal caregivers. She underwent training, but attempts to train her caregivers were unsuccessful. One of them reported not knowing how to use a computer and kept distance, whereas the other briefly stayed in the bedroom and did not wait until the end of the explanation. At reassessment, PP2 stated that she found the equipment useful, responding positively to all the assessment instruments. However, when downloading the system data records through the Web application, it was found that she did not use it at all. Subsequently, the occupational therapist who assists her weekly reported that she has a depressed mood, difficulty in accepting the diagnosis, and a high expectation of improvement. During the semi-structured interview, she mentioned difficulties to manipulate the computer, start using it, without the assistance of the caregivers.

- - PP4: 48 year-old male.

Diagnosed with spinal cord injury (C5 level) after a gunshot wound during a robbery in 2016. He is very dependent regarding performance of ADL, and needs assistance to carry out all activities. During the day, he remains seated in an armchair in the living room watching TV. His main complaint is having to depend on others for everything. He was married, but his wife filed for divorce five months after the accident. Currently, he is living with his mother, who helps him, but he also has formal caregivers hired by his health medical organization. On the day of installation and training, he seemed interested and showed that he understood the

system operation, but his caregiver showed no interest. At reassessment, he responded to all the instrument questions and reported having used the SE during all days of the week. However, no data were found in the system usage record. In the interview, he reported lack of total independence as a difficulty with the equipment.

Both of these participants present some similar characteristics that may have contributed to not using the equipment: they have difficulty dealing with the diagnosis or with their current health condition; caregivers not close or not engaged in this additional task; they report that the equipment does not allow total independence and that they have little knowledge of technology, limited to communication through the smartphone.

Costa et al. [97] found some factors that can contribute to the understanding of the non-use of SEs: lack of equipment functionality (for not providing the desired independence), difficulty in use, embarrassment in using the device, lack of support from family members, lack of user motivation. Their study focused on other equipment, but it gives evidence of important aspects that can lead to the non-use or abandonment of AT devices.

With regard to non-acceptance of diagnoses, studies have shown that this is an important factor to be considered when prescribing or selecting an AT device or resource [49,98]; however, in the present study, this information only appeared at the time of reevaluation.

Wessels et al. [49] reported that there is a difference in the way the AT resource will be viewed between people who were born with a disability (for them, technology opens a new range of possibilities) and those who have acquired a disability (because, for them, technology will never replace the lost function).

Volunteers PP2 and PP4 are in the second group, since they acquired the disability as adults. A recurring line in the interviews is that they were very active and independent

people in the past, and now they find themselves depending on others for practically all activities. For them, in addition to the loss of independence, the disability has also brought other types of losses, such as moving from their hometowns, changing their standard of living, ending their relationships, or losing their jobs. Such cases often result in periods of depression [49]. In this sense, for these people, it is hypothesized that they would only benefit from technology if their dependency could be completely reversed.

Another associated factor that may have contributed to non-use of the SE is that, for many people, the AT device highlights the disability [42,47,98]. A study conducted by Verza et al. [98] with patients diagnosed with MS found that 30.3% of the reasons for abandoning or not using an AT device are due to the patient's non-acceptance. Those authors explain that, although the AT device is seen as a possibility to increase functionality, it can be interpreted by the patient as a validation of their disability and loss of independence, resulting in decreased self-esteem.

It should be noted that, although the system registers activation of the equipment, this information was not passed on to the participants, so that the use of the system was based on their real needs and desires, and not on the fact that they felt obliged to use it.

It is worth restating that, in view of therapeutic objectives, it is important that the professional involved perform a wide and in-depth assessment of the patient's real demands, their expectations, and possibilities of the proposed AT device, as well as consider their participation in the choice. These points are important to ensure acceptance and continuity of use, since abandonment can represent a waste of time and financial expenses (their own or of the government) [40,48,50,51].

Conclusions

Volunteers who used the IROG-controlled SE system showed better occupational performance and satisfaction with performance. In addition, the psychosocial impact of using this system was close to the maximum and, for three participants, satisfaction with the system was well evaluated and the system was considered of good usability. The volunteer who had a more pronounced motor impairment and, therefore, more difficulty in using the system, rated its psychosocial impact and usability with lower scores.

Suggestions for system improvement were associated with changes in the interface aiming at fewer commands to select TV channels; eliminating the need to use the notebook computer keyboard as much as possible and simplifying steps, enabling greater independence for people with disability to use the system; increasing the possibilities of equipment and devices that can be controlled (e.g., fixed residential lighting and smartphones).

The two participants who did not use the system presented characteristics such as non-acceptance of their diagnoses or current health conditions, weak relationship with caregivers, and need for assistance with using the equipment. In contrast, the fact that an AT device possibly reinforces dependence may have corroborated these results.

The HAAT model was used as a basis for definition of the study methodology aiming to achieve its objectives.

Although the AT resource (SE system) was previously defined, in order to be evaluated the choice of volunteers was not random, and professionals were asked to indicate the possible participants. Regarding the Activity component, the activities considered important for each participant (controlling the TV for entertainment, controlling lighting and/or fan) were evaluated according to their wishes. Although there were more possibilities of equipment available for control, only those that the

participant was interested in were installed. Still in the Activity component, it was considered the most familiar place to conduct the research, that is, each of the volunteer’s homes.

Regarding the Human component, the motor and cognitive skills of the volunteer were taken into account. As the study objective was to evaluate the use of the system by people with severe motor disabilities, in the least intrusive way possible, the eye tracker using IROG technology was defined as the most suitable device for this purpose. Motivation to resume performance of the activities was assessed through the COPM in order to cover the important activities to be carried out.

With respect to the Context component, it was noticed that the volunteers who had greater social support (from family members and/or formal caregivers) actually used the SE and were able to provide a more in-depth assessment. Those who did not have this support, or had it in a weakened way, did not use the SE.

Finally, with regard to the Assistive Technology component, it was evaluated that the SE system functioned as a facilitator to carry out the desired activities, enabling greater exercise of autonomy and independence.

Concerning the ICF, its components can also be broken down into the elements of this research. Health Condition considered the disability or the diagnosis of the participants. In Body Functions and Structures, the movement limitations resulting from the participants’ health conditions interfered in the way each of them interacted with the electronic equipment. Activities important for the volunteers and their Participation were assessed through the FIM™ and COPM instruments. Personal Factors (satisfaction and psychosocial impact) were verified using the QUEST 2.0 and PIADS instruments. Finally, with regard to Environmental Factors, the SE system, as in the HAAT model, is considered a facilitator, and its usability was assessed through the SUS scale.

Considering that disability is indicated by the HAAT model and the ICF as inherent in social structures, and not in the person, the results found suggest that the SE system enabled reduction in the incapacity of the volunteers, who thus had greater participation in related activities.

The main contribution of this study was the wide evaluation of equipment that aims to allow greater independence for people with severe motor disabilities, from the point of view of its operation and usability, as well as the benefits it provided to the people who used it. Several assessment instruments were used to achieve the study objectives. In addition, a system user manual containing the steps for its effective use was also produced.

For professionals, this study highlights the importance of a good evaluation for the prescription and development of AT resources, as well as new possibilities to provide people with severe motor disabilities with greater independence to carry out their everyday activities with regard to equipment control, avoiding abandonment or non-use.

Future studies with larger samples and longer duration should be conducted, expanding the possibilities of controlled equipment and devices, in order to understand whether the benefits remain in long term.

Patents

The gBox patent, together with the environment control system named "Remote micro-controlled device for charging residential loads via the Internet with emitter and receiver of commands via integrated infrared", was submitted to the Institute of Technological Innovation – INIT at UFES, Brazil, and evaluation is under process.

Acknowledgments

The authors would like to thank all the professionals and participants involved in this study for their contribution.

References

1. Kadam R, Mahamuni P, Parikh Y. Smart Home System. International Journal of Innovative Research in Advanced Engineering. 2015; 2:81-86.
2. Rivera-Illingworth F, Callaghan V, Hagras H. A neural network agent based approach to activity detection in AmI environments. In: Proceedings of IEEE International Workshop on Intelligent Environments; 28-29th June 2005; Colchester (United Kingdom). Institution of Engineering and Technology; 2005. 1-12. doi: 10.1049/ic:20050222.
3. Huang B, Tian G, Wu H, Zhou F. A method of abnormal habits recognition in intelligent space. Engineering Applications of Artificial Intelligence. 2014; 29:125-133. doi: 10.1016/j.engappai.2013.12.010.
4. Lupalu J, Bouchard K, Bouzouane A, Bouchard B, Giroux B. Unsupervised mining of activities for smart home prediction. Procedia Computer Science. 2013; 19:503-510. doi: https: 10.1016/j.procs.2013.06.067.
5. Rashidi P, Cook DJ, Holder LB, Schmitter-Edgecombe M. Discovering activities to recognize and track in a smart environment. IEEE Transactions on Knowledge and Data Engineering. 2011; 23:527-539. doi: 10.1109/TKDE.2010.148.
6. Luhr S, West G, Venkatesh S. Recognition of emergent human behaviour in a smart home: A data mining approach. Pervasive and Mobile Computing. 2007; 3:95-116. doi: 10.1016/j.pmcj.2006.08.002.

7. Tello RJMG, Bissoli ALC, Ferrara F, Müller S, Ferreira A, Bastos-Filho TF. Development of a Human Machine Interface for Control of Robotic Wheelchair and Smart Environment. IFAC-PapersOnLine. 2015; 48:136-141. doi: 10.1016/j.ifacol.2015.12.023.

8. Hussein A, Adda M, Atieh M, Fahs W. Smart Home Design for Disabled People based on Neural Networks. Procedia Computer Science. 2014; 37:117-126. doi: 10.1016/j.procs.2014.08.020.

9. Rampinelli M, Covre VB, Queiroz FM, Vassallo RF, Bastos-Filho TF, Mazo M. An intelligent space for mobile robot localization using a multi-camera system. Sensors. 2014; 14:15039-15064. doi: 10.3390/s140815039.

10. Adami I, Antona M, Stephanidis C. Ambient Assisted Living for the Motor Impaired. In. Koroupetroglou G, editor. Assistive Technologies, Disability Informatics and Computer Access for Motor Limitations. USA: IGI Global; 2013, pp. 76-104.

11. Bastos Filho TF, Fernandes MR, Lucena-Jr VF, Pereira CE. Proposal of Architecture for Integration of a Wheelchair in an Intelligent Space. In: Proceedings of 4th IEEE Biosignals and Biorobotics Conference (ISSNIP); 18-20th February 2013; Rio de Janeiro-RJ (Brazil); 2013. 6-11.

12. Ocepek J, Roberts AEK, Vidmar G. Evaluation of treatment in the smart home IRIS in terms of functional independence and occupational performance and satisfaction. Computational and Mathematical Methods in Medicine. 2013; 2013:10 pages. doi: 10.1155/2013/926858.

13. Gentry T. Smart homes for people with neurological disability: State of the art. NeuroRehabilitation. 2009; 25:209-217. doi: 10.3233/NRE-2009-0517.

14. Muñoz D, Fortes P. O princípio da autonomia e o consentimento livre e esclarecido. In. Iniciação à Bioética. Costa SIF, Oselka G, Garrafa V, coordinators. Brasília: CFM; 1998. pp. 53-70.
15. Martin S, Kelly G, Kernohan WG, McCreight B, Nugent C. Smart home technologies for health and social support. Cochrane Database of Systematic Reviews. 2008; 4:1-10. doi: 10.1002/14651858.CD006412.pub2.
16. Brandt Å, Samuelsson K, Töytäri O, Salminen A-L. Activity and participation, quality of life and user satisfaction outcomes of environmental control systems and smart home technology: a systematic review. Disability and Rehabilitation: Assistive Technology. 2011; 6(3):189-206. doi: 10.3109/17483107.2010.532286.
17. Marikyan D, Papagiannidis S, Alamanos E. A systematic review of the smart home literature: A user perspective. Technological Forecasting & Social Change. 2019; 138:139-154. doi: 10.1016/j.techfore.2018.08.015.
18. Bissoli A, Lavino-Junior D, Sime M, Encarnação L, Bastos-Filho T. A Human-Machine Interface Based on Eye Tracking for Controlling and Monitoring a Smart Home Using the Internet of Things. Sensors. 2019; 19:859-884. doi: 10.3390/s19040859.
19. World Health Organization – WHO. World Report on Disability. WHO Press; Printed in Malta; 2011. Available from: https://www.who.int/disabilities/world_report/2011/en/.
20. Siqueira SC, Vitorino PVO, Prudente COM, Santana TS, Melo GF. Quality of life of patients with Amyotrophic Lateral Sclerosis. Rev Rene. 2017; 18(1):139-146. doi: 10.15253/2175-6783.2017000100019.
21. Marrie RA, Cutter GR, Tyry T, Cofield SS, Fox R, Salter A. Upper limb impairment is associated with use of assistive devices and unemployment in

multiple sclerosis. Multiple Sclerosis and Related Disorders. 2017; 13:87-92. doi: 10.1016/j.msard.2017.02.013.

22. Shamshiri H, Fatehi F, Abolfazli R, Harirchian MH, Sedighi B, Zamani B, et al. Trends of quality of life changes in amyotrophic lateral sclerosis patients. J Neurol Sci. 2016; 368:35-40. doi: 10.1016/j.jns.2016.06.056.
23. Bertoni R, Lamers I, Chen CC, Feys P, Cattaneo D. Unilateral and bilateral upper limb dysfunction at body functions, activity and participation levels in people with multiple sclerosis. Mult Scler. 2015; 21(12):1566-1574. doi: 10.1177/1352458514567553.
24. Souza FDA, Cruz DMC, Ferrigno ISV, Tsukimoto GR, Figliolia CS. Correlação entre papéis ocupacionais e independência de usuários com lesão medular em processo de reabilitação. Mundo da Saúde. 2013; 37(2):166-175. doi: 10.15343/0104-7809.2013372166175.
25. Riazi A, Bradshaw SA, Playford ED. Quality of life in the care home: a qualitative study of the perspectives of residents with multiple sclerosis. Disabil Rehabil. 2012; 34(24):2095-2102. doi: 10.3109/09638288.2012.672539.
26. Lee YC, Chen YM, Hsueh IP, Wang YH, Hsieh CL. The impact of stroke: insights from patients in Taiwan. Occup Ther Int. 2010; 17(3):152-158. doi: 10.1002/oti.301.
27. Wolf TJ, Baum C, Connor LT. Changing face of stroke: Implications for occupational therapy practice. AJOT. 2009; 63:621-625. doi: 10.5014/ajot.63.5.621.
28. Groot V, Beckerman H, Lankhorst GJ, Polman CH, Bouter LM. The initial course of daily functioning in multiple sclerosis: a three-year follow-up study. Mult Scler. 2005; 11:713-718. doi: 10.1191/1352458505ms1238oa.

29. Pedretti LW, Early ME. Desempenho ocupacional e modelos de prática para disfunção física. In. Pedretti LW, Early ME. Terapia ocupacional: capacidades práticas para disfunções físicas. São Paulo: Roca; 2004.
30. Camacho A, Esteban J, Paradas C. Informe de la Fundación del Cerebro sobre el impacto social de la esclerosis lateral amiotrófica y las enfermedades neuromusculares. Neurología. 2015; 33:35-46. doi: 10.1016/j.nrl.2015.02.003.
31. World Health Organization – WHO. International Classification of Functioning, Disability and Health: ICF. World Health Organization; 2001.
32. Farias N, Buchalla CM. A Classificação Internacional de Funcionalidade, Incapacidade e Saúde da Organização Mundial da Saúde: conceitos, usos e perspectivas. Rev Bras Epidemiol. 2005; 8(2):187-193. doi: 10.1590/S1415-790X2005000200011.
33. Dahl TH. International classification of functioning, disability and health: an introduction and discussion of its potential impact on rehabilitation services and research. J Rehabil Med. 2002; 34:201-204. doi: 10.1080/165019702760279170.
34. Brasil. CORDE. Comitê de Ajudas Técnicas, ATA VII; 2008. Available from: <http://www.mj.gov.br/sedh/ct/corde/dpdh/corde/comite_at.asp>.
35. United States Congress. Assistive Technology Act of 2004: Amendment to the assistive technology act of 1998 (Public Law 108–364); 2004.
36. Luzo MCM, Mello MAF, Capanema VM. Recursos tecnológicos em terapia ocupacional – órteses e tecnologia assistiva. In. De Carlo MMRP, Luzo MCM, organizers. Terapia ocupacional: reabilitação física e contextos hospitalares. São Paulo: Roca; 2004. pp. 99-126.
37. Cook A, Polgar J. Assistive Technologies: Principles and Practice. 4th ed. St. Louis: Mosby Elselvier; 2015.

38. Pelosi MB, Nunes L. Formação em serviço de profissionais da saúde na área de tecnologia assistiva: o papel do terapeuta ocupacional. Rev Bras Crescimento Desenvolv Hum. 2009; 19(3):435-444.
39. Borg J, Lindström A, Larsson S. Assistive technology in developing countries: national and international responsibilities to implement the Convention on the Rights of Persons with Disabilities. Viewpoint. Lancet. 2009; 374(28):1863-1865. doi: 10.1016/S0140-6736(09)61872-9.
40. Jamwal R, Callaway L, Ackerl J, Farnworth L, Winkler D. Electronic assistive technology used by people with acquired brain injury in shared supported accommodation: Implications for occupational therapy. British Journal of Occupational Therapy. 2017; 80(2):89-98. doi: 10.1177/0308022616678634.
41. Cruz DMC, Ioshimoto MTA. Tecnologia assistiva para as atividades de vida diária na tetraplegia completa C6 pós-lesão medular. Rev Triang: Ens. Pesq. Ext. Uberaba. 2010; 3(2):177-190. doi: 10.18554/rt.v3i2.153.
42. Andrade VS, Pereira LSM. Influência da tecnologia assistiva no desempenho funcional e na qualidade de vida de idosos comunitários frágeis: uma revisão bibliográfica. Rev Bras Geriatr Gerontol. 2009; 12(1):113-122. doi: 10.1590/1809-9823.2009120110.
43. Plotkin A, Sela L, Weissbrod A, Kahana R, Haviv L, Yeshurun Y, et al. Sniffing enables communication and environmental control for the severely disabled. PNAS. 2010; 107(32):14413-14418. doi: 10.1073/pnas.1006746107.
44. Lulé D, Zickler C, Häcker S, Bruno MA, Demertzi A, Pellas F, et al. Life can be worth living in locked-in syndrome. Progress in Brain Research. 2009; 177:339-351. doi: 10.1016/S0079-6123(09)17723-3.

45. Dusik CL, Santarosa L. Mousekey, Teclado Virtual Silábico-Alfabético: Tecnologia Assistiva para Pessoas com Deficiência Física. In: Memorias XVIII Congreso Internacional de Informática Educativa. Nuevas Ideas en Informática Educativa 9 – TISE 2013; Porto Alegre-Brasil. Digitalizado en Chile; 2013. 90-98.
46. Lenker JA, Harris F, Taugher M, Smith RO. Consumer perspectives on assistive technology outcomes. Disabil Rehabil Assist Technol. 2013; 8(5):373-380. doi: 10.3109/17483107.2012.749429.
47. Squires LA, Williams N, Morrison VL. Matching and accepting assistive technology in multiple sclerosis: A focus group study with people with multiple sclerosis, carers and occupational therapists. Journal of Health Psychology. 2016; 24:1-15. doi: 10.1177/1359105316677293.
48. Cruz DMC, Emmel MLG, Manzini MG, Mendes PVB. Assistive technology accessibility and abandonment: challenges for occupational therapists. The Open Journal of Occupational Therapy. 2016; 4(1):Article 10. doi: 10.15453/2168-6408.1166.
49. Wessels R, Dijcks B, Soede M, Gelderblom GJ, De Witte L. Non-use of provided assistive technology devices: a literature overview. Technology and Disability. 2003; 15(4):231-238. doi: 10.3233/TAD-2003-15404.
50. Riemer-Reiss ML, Wacker RR. Factors associated with assistive technology discontinuance among individuals with disabilities. Journal of Rehabilitation. 2000; 66(3):44-50.
51. Phillips B, Zhao H. Predictors of assistive technology abandonment. Assistive Technology. 1993; 5(1):36-45. doi: 10.1080/10400435.1993.10132205.

52. Alves ACJ, Matsukura TS. Revisão sobre avaliações para indicação de dispositivos de tecnologia assistiva. Rev Ter Ocup Univ São Paulo. 2014; 25(2):199-207. doi: 10.11606/issn.2238-6149.v25i2p199-207.
53. Lovarini M, Mccluskey A, Curtin M. Editorial: Critically appraised papers. Limited high-quality research in the effectiveness of assistive technology. Aust Occup Ther J. 2006; 50(1):50.
54. Gelderblom GJ, De Witte LP. The assessment of assistive technology outcomes, effects and costs. Technol and Disabil. 2002; 14(3):91-94. doi: 10.3233/tad-2002-14302.
55. Alper S, Raharinirina S. Assistive technology for individuals with disabilities: a review and synthesis of the literature. Journal of Special Education Technology. 2006; 21(2):47-64. doi: 10.1177/016264340602100204.
56. Kinney A, Goodwin DM, Gitlow L. Measuring assistive technology outcomes: a user centered approach. Assistive Technology Outcomes and Benefits. 2016; 10:94-110.
57. Peterson DB, Murray GC. Ethics and assistive technology service provision. Disabil Rehabil Assist Technol. 2006; 1:59-67. doi: 10.1080/09638280500167241.
58. Rocha EF, Castiglioni MC. Reflexões sobre recursos tecnológicos: ajudas técnicas, tecnologia assistiva, tecnologia de assistência e tecnologia de apoio. Rev Ter Ocup Univ São Paulo. 2005; 16(3):97-104. doi: 10.11606/issn.2238-6149.v16i3p97-104.
59. Netten JJ, Dijkstra PU, Geertzen JHB, Postema K. What influences a patient's decision to use custom-made orthopaedic shoes? BMC Musculoskeletal Disorders. 2012; 13:92. doi: 10.1186/1471-2474-13-92.

60. Zhao Y, Zhang X, Crabtree J. Human-computer interaction and user experience in smart home research: a critical analysis. Issues in Information Systems. 2016; 17(III):11-19.

61. Cook A, Polgar J. Cook and Hussey's Assistive Technologies: Principles and Practice. 3rd ed. St. Louis: Mosby Elselvier; 2008.

62. Rampinelli M, Bastos TF, Vassallo RF, Pizarro D. Implementation of an intelligent space for localizing and controlling a robotic wheelchair. In: 2012 ISSNIP Biosignals and Biorobotics Conference: Biosignals and Robotics for Better and Safer Living (BRC); 9-11th January 2012; Manaus-Brazil: IEEE – Institute of Electrical and Electronics Engineers; 2012. 1-4. doi: 10.1109/BRC.2012.6222187.

63. Lee JH, Hashimoto H. Intelligent Space: concept and contents. Advanced Robotics. 2002; 16(3):265-280. doi: 10.1163/156855302760121936.

64. Aarts EH, Encarnação JL. Into Ambient Intelligence. In. Aarts EH, Encarnação JL, editors. True visions: The emergence of ambient intelligence. Springer: Netherlands; 2008. pp. 1-16.

65. Aldrich F. Smart homes: past, present and future. In. Harper R, editor. Inside the Smart Home. Springer: London; 2003. pp. 17-39.

66. Ding Z, Luo J, Deng H. Accelerated exhaustive eye glints localization method for infrared video oculography. In: SAC '18 – Proceedings of the 33rd Annual ACM Symposium on Applied Computing; April 2018; Pau France. New York-NY (United States of America): Association for Computing Machinery; 2018. 620–627. doi: https://doi.org/10.1145/3167132.3167200.

67. Eid MA, Giakoumidis N, El-Saddik A. A Novel Eye-Gaze-Controlled Wheelchair System for Navigating Unknown Environments: Case Study with a Person with ALS. IEEE Access. 2016; 4:558-573. doi:10.1109/ACCESS.2016.2520093.

68. Giannotto EC. Uso de rastreamento do olhar na avaliação da experiência do tele-usuário de aplicações de TV interativa. M.Sc. Thesis, Polytechnic School of the University of São Paulo. Department of Computer Engineering and Digital Systems. 2009, 290 p. Available from: https://teses.usp.br/teses/disponiveis/3/3141/tde-15042009-151212/pt-br.php.

69. Duchowski AT. Eye tracking methodology: Theory and practice. 2nd ed. London: Springer-Verlag; 2007.

70. Anders S, Weiskopf N, Lule D, Birbaumer N. Infrared oculography—validation of a new method to monitor startle eyeblink amplitudes during fMRI. NeuroImage. 2004; 22(2):767-770. doi: 10.1016/j.neuroimage.2004.01.024.

71. Sharma A, Abrol P. Eye gaze techniques for human computer interaction: a research survey. International Journal of Computer Applications. 2013; 71(9):18-29. doi: 10.5120/12386-8738.

72. Van Es MA, Hardiman O, Chio A, Al-Chalabi A, Pasterkamp RJ, Veldink JH, et al. Amyotrophic lateral sclerosis. The Lancet. 2017; 390(10107):2084-2098. doi: 10.1016/S0140-6736(17)31287-4.

73. Tobii Gaming. Tobii Eye Tracker 4C. Available from: https://gaming.tobii.com/product/tobii-eye-tracker-4c/. Access in: February 24, 2019.

74. Tobii Dynavox. Gaze Point. Available from: https://www.tobiidynavox.com/en-us/software/free-resources/gaze-point-1/. Acess in: February 24, 2019.

75. Riberto M, Miyazaki MH, Jucá SSH, Sakamoto H, Pinto PPN, Battistella LR. Validação da Versão Brasileira da Medida de Independência Funcional. Acta Fisiatr. 2004; 11(2):72-76. doi: 10.5935/0104-7795.20040003.

76. Linacre JM, Heinemann AW, Wright BD, Granger CV, Hamilton BB. The structure and stability of the Functional Independence Measure. Arch Phys Med Rahabil. 1994; 75:127-132. doi: 10.1016/0003-9993(94)90384-0.

77. Law M, Baptiste S, Carswell A, McColl M, Polatajko H, Pollock N. Medida canadense de desempenho ocupacional (COPM). Magalhães LC, Magalhães, LV, Cardoso AA, organizers and translators. Belo Horizonte: Ed UFMG; 2009.

78. Jutai J, Day H. Psychosocial Impact of Assistive Devices Scale (PIADS). Technol and Disabil. 2002; 14:107-111. doi: 10.1037/t45599-000.

79. Demers L, Weiss-Lambrou R, Ska B. The Quebec User Evaluation of Satisfaction with Assistive Technology (QUEST 2.0): an overview and recent progress. Technol and Disabil. 2002; 14:101-105. doi: 10.13072/midss.298.

80. Carvalho KEC, Gois Junior MB, Sá KN. Tradução e validação do Quebec User Evaluation of Satisfaction with Assistive Technology (QUEST 2.0) para o idioma português do Brasil. Rev. Bras. Reumatol. 2014; 54(4):260-267. doi: 10.1016/j.rbr.2014.04.003.

81. Brooke J. SUS: a "quick and dirty" usability scale. In: Jordan PW, Thomas B, McClelland IL, Weerdmeester B, editors. Usability Evaluation in Industry. London: Taylor and Francis; 1996. 189-194.

82. Calvo A, Chiò A, Castellina E, Corno F, Farinetti L, Ghiglione P, et al. Eye tracking impact on Quality-of-life of ALS patients. In: Computers Helping People with Special Needs. Proceedings of 11th International conference on computers helping people with special needs; 9-11th July 2008; Linz (Austria): Springer-Verlag Berlin Heidelberg; 2008. 70-77. doi: 10.1007/978-3-540-70540-6.

83. Myburg M, Allan E, Nalder E, Schuurs S, Amsters D. Environmental control systems – the experiences of people with spinal cord injury and the implications for

prescribers. Disability and Rehabilitation: Assistive Technology. 2017; 12(2):128-136. doi: 10.3109/17483107.2015.1099748.

84. Demers L, Wessels R, Weiss-Lambrou R, Ska B, de Witte LP. Key dimensions of client satisfaction with assistive technology: a cross-validation of a Canadian measure in the Netherlands. J Rehabil Med. 2001; 33:187-191. doi: 10.1080/165019701750300663.
85. Shone SM, Ryan S, Rigby PJ, Jutai JW. Toward a comprehensive evaluation of the impact of electronic aids to daily living: evaluation of consumer satisfaction. Disabil Rehabil. 2002; 24:115-125. doi: 10.1080/09638280110066794.
86. Day H, Jutai J. Measuring the psychosocial impact of assistive devices: the PIADS. Canadian Journal of Rehabilitation. 1996; 9(2):159-168.
87. Bangor A, Kortum P, Miller JA. The System Usability Scale (SUS): An Empirical Evaluation. International Journal of Human-Computer Interaction. 2008; 24(6):574-594. doi: 10.1080/10447310802205776.
88. Little R. Electronic aids for daily living. Phys Med Rehabil Clin N Am. 2010; 21:33-42. doi: 10.1016/j.pmr.2009.07.008.
89. Boman I-L, Tham K, Granqvist A, Bartfai A, Hemmingsson H. Using electronic aids to daily living after acquired brain injury: A study of the learning process and the usability. Disability and Rehabilitation: Assistive Technology. 2007; 2(1):23-33. doi: 10.1080/17483100600856213.
90. Boustany G, Itani AED, Youssef R, Chami O, Abu-Faraj ZO. Design and development of a rehabilitative eye-tracking based home automation system. In: Proceedings of 2016 3rd Middle East Conference on Biomedical Engineering (MECBME); 6-7th October 2016; Beirut (Lebanon). Institute of Electrical and

Electronics Engineers (IEEE): Curran Associates, Inc.; Jan 2017. 30-33. doi: 10.1109/MECBME.2016.7745401.

91. Brennan CP, McCullagh PJ, Galway L, Lightbody G. Promoting autonomy in a smart home environment with a smarter interface. In: Proceedings of 2015 37th Annual International Conference of the IEEE Engineering in Medicine and Biology Society (EMBC); August 25-29 2015; MiCo – Milano Conference Center, Milan (Italy). Institute of Electrical and Electronics Engineers (IEEE): Curran Associates, Inc.; Dec 2015. 5032-5035. doi: 10.1109/EMBC.2015.7319522.
92. Hooper B, Verdonck M, Amsters D, Myburg M, Allan E. Smart-device environmental control systems: experiences of people with cervical spinal cord injuries. Disability and Rehabilitation: Assistive Technology. 2018; 13(8):24-730. doi: 10.1080/17483107.2017.1369591.
93. Verdonck MC, Chard G, Nolan M. Electronic aids to daily living: be able to do what you want. Disability and Rehabilitation: Assistive Technology. 2010; 6(3):268-281. doi:10.3109/17483107.2010.525291.
94. Judge S, Robertson Z, Hawley MS. Users' Perceptions of Environmental Control Systems. In: Emiliani PL, Burzagli L, Como A, Gabbanini F, Salminen A-L (eds.). Assistive Technology from Adapted Equipment to Inclusive Environments - AAATE 2009; August 31st–September 2nd 2009; Florence (Italy). Amsterdam: IOS Press; 2009. 426-431.
95. Harmer J, Bakheit AMO. The benefits of environmental control systems as perceived by disabled users and their carers. British Journal of Occupational Therapy. 1999; 62(9):394-398. doi: 10.1177/030802269906200902.

96. Palmer P, Seale J. Exploring the attitudes to environmental control systems of people with physical disabilities: a grounded theory approach. Technol Disabil. 2007; 9(1):17-27. doi: 10.3233/TAD-2007-19103.

97. Costa CR, Ferreira FMRM, Bortolus MV, Carvalho MGR. Dispositivos de tecnologia assistiva: fatores relacionados ao abandono. Cad. Ter. Ocup. UFSCar. 2015; 23(3):611-624. doi: 10.4322/0104-4931.ctoAR0544.7.8.

98. Verza R, Carvalho ML, Battaglia MA, Uccelli MM. An interdisciplinary approach to evaluating the need for assistive technology reduces equipment abandonment. Mult Scler. 2006; 12(1):88-93. doi: 10.1191/1352458506ms1233oa.

Figure 1 Click here to download Figure Fig 1.tif

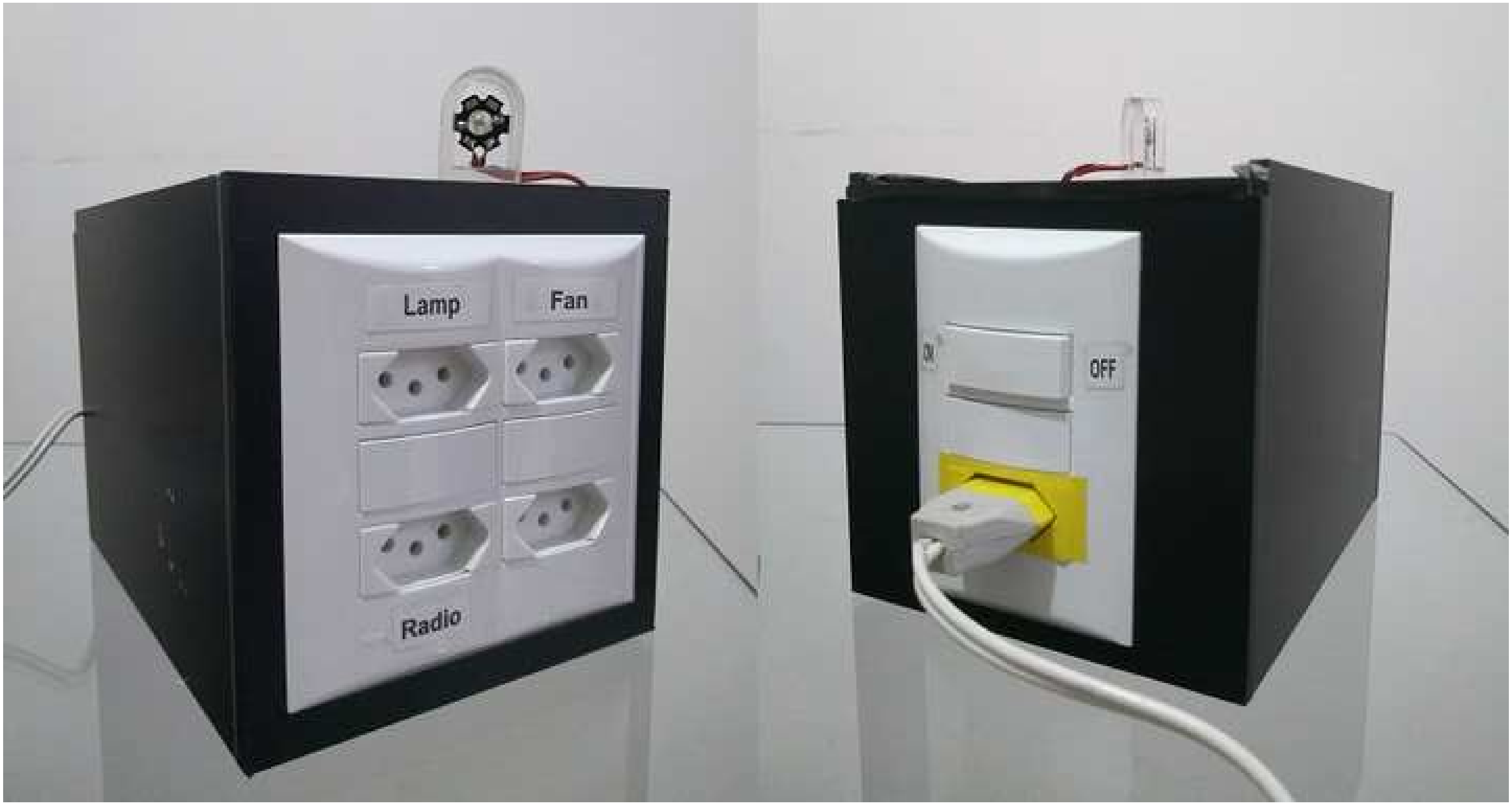

Figure 2

Click here to download Figure Fig 2.tif

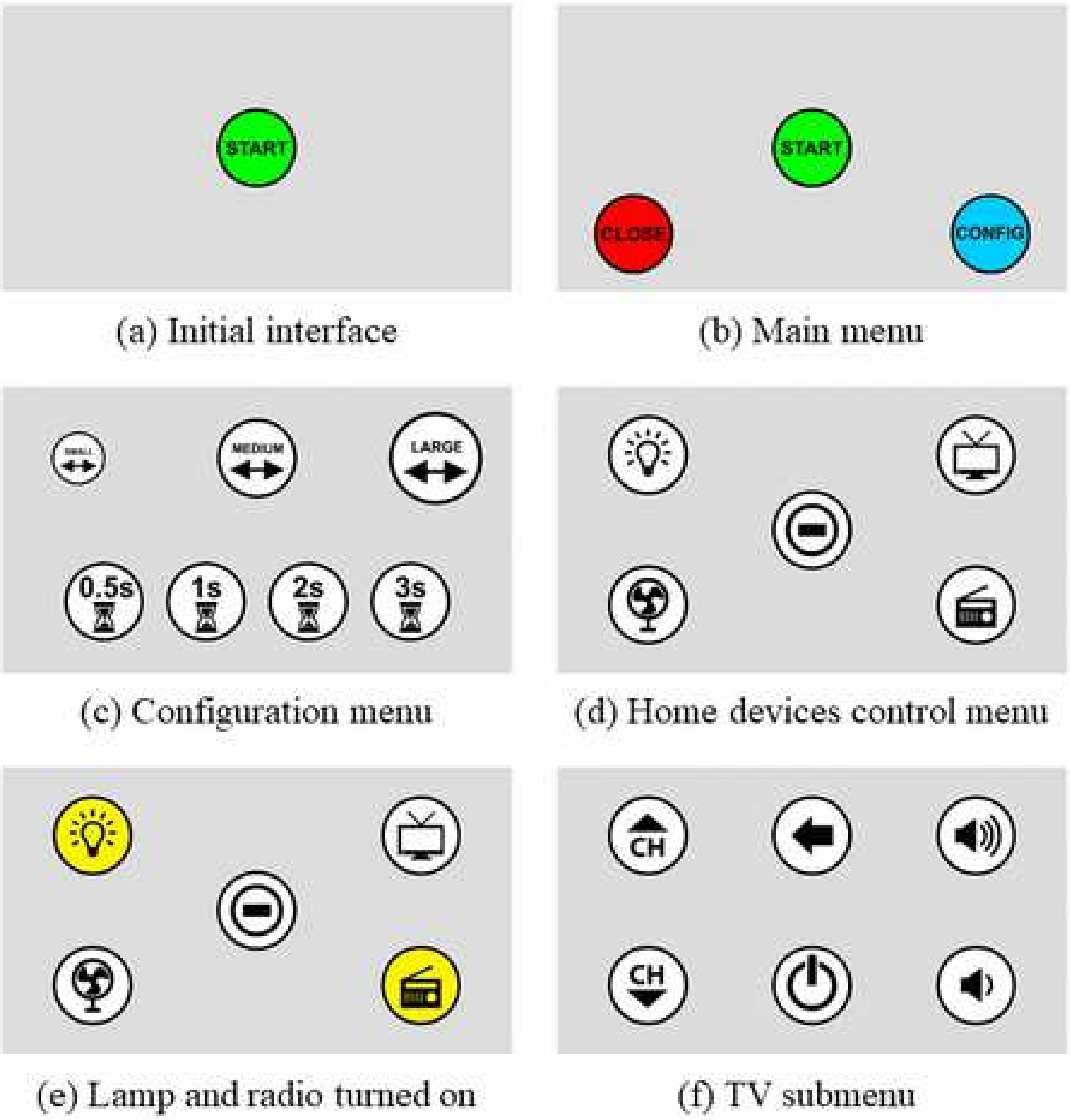

Figure 3

Click here to download Figure Fig 3.tif

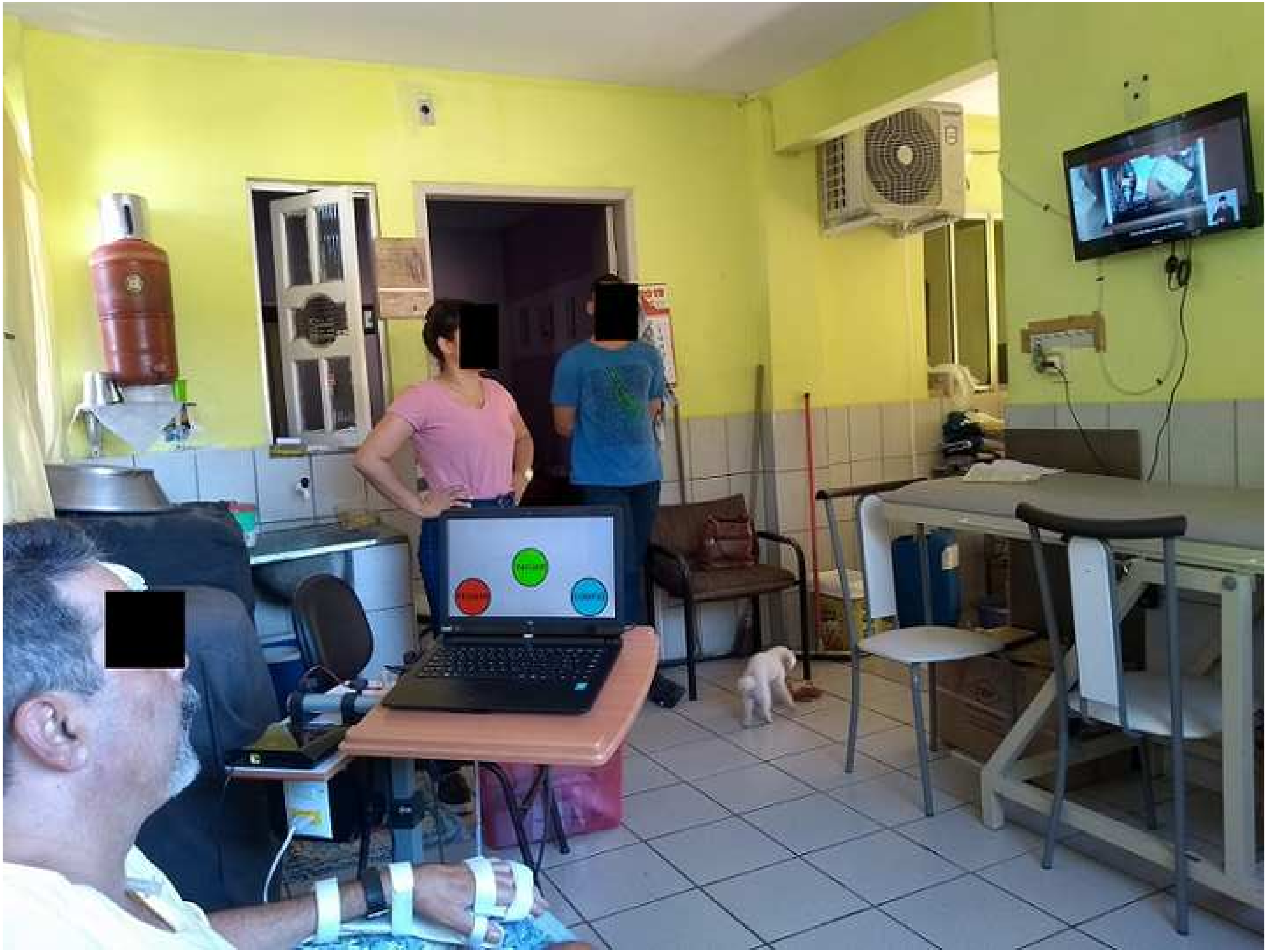

Figure 4

Click here to download Figure Fig 4.tif

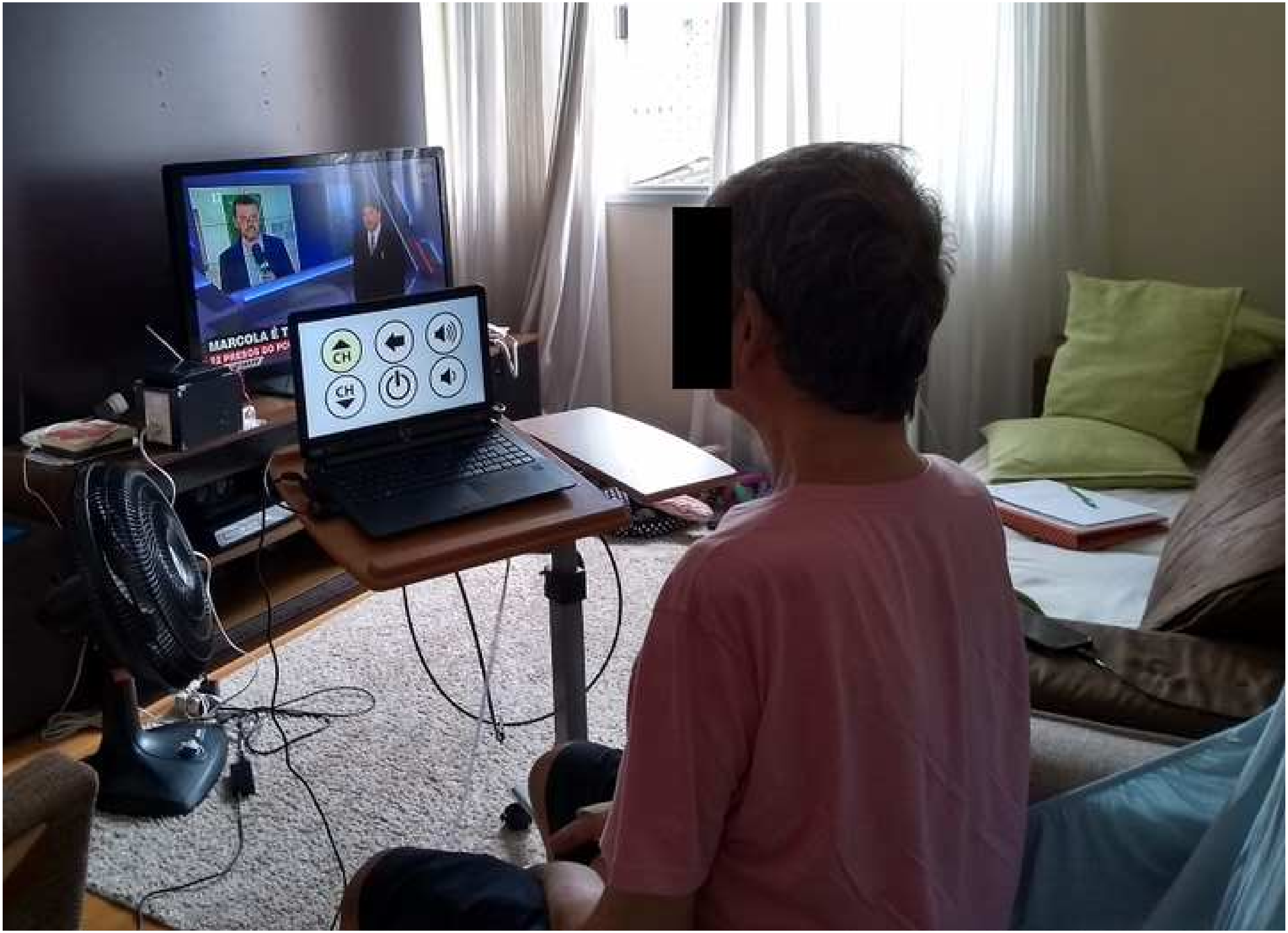

Figure 5 Click here to download Figure Fig 5.tif

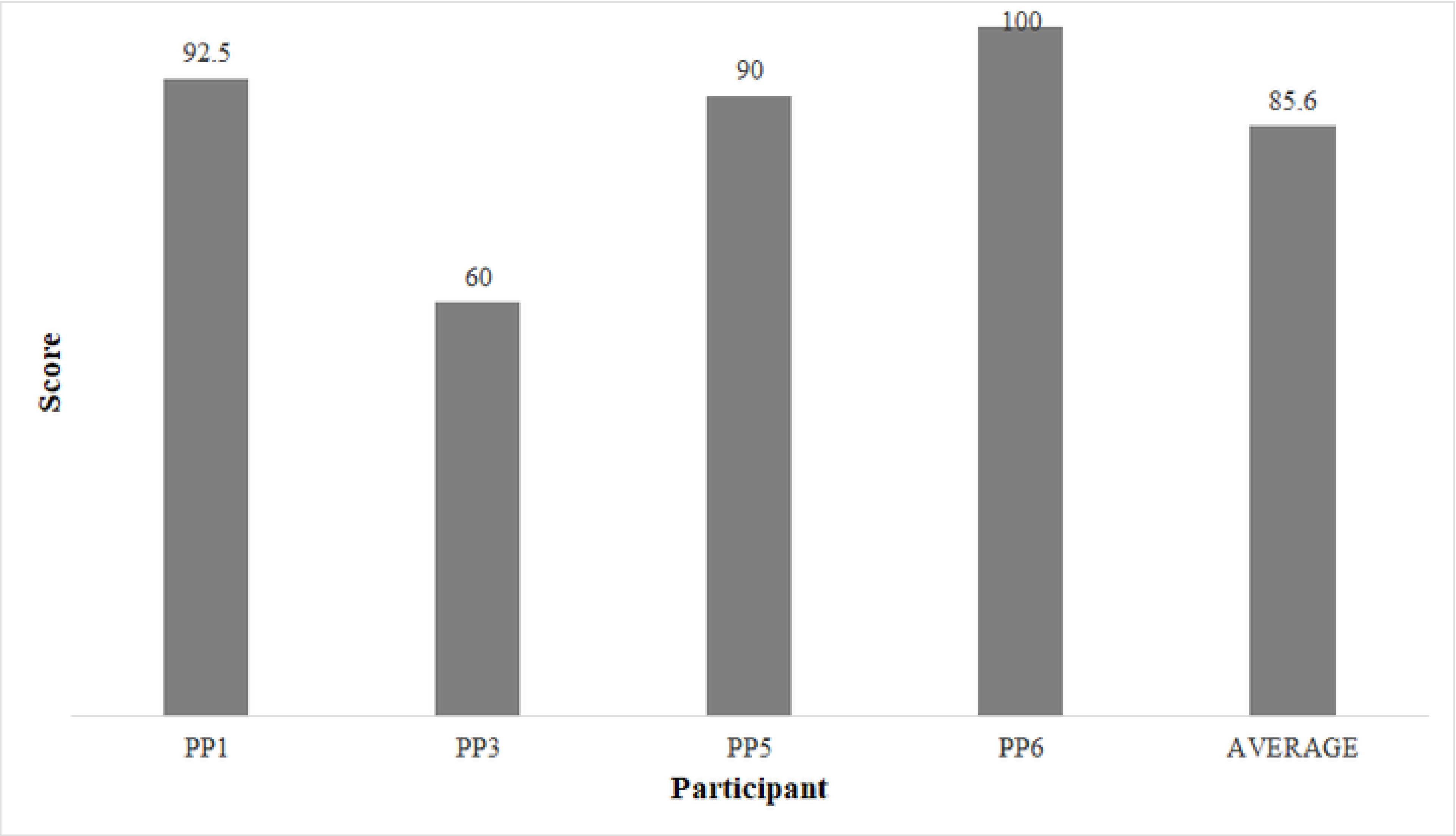

Supplement: Attachment — Submitted filename: PONE-D-20-12562_reviewer.pdf [file pone.0256062.s002.pdf]
