# Supplementary material for: Usability, occupational performance and satisfaction evaluation of a smart environment controlled by infrared oculography by people with severe motor disabilities
Source: PLoS One. 2021 Aug 13;16(8):e0256062. doi: 10.1371/journal.pone.0256062 (PMC8362986; doi:10.1371/journal.pone.0256062)
Supplement: Attachment — Submitted filename: PONE-D-20-12562_R1.pdf [file pone.0256062.s004.pdf]

PLOS ONE

Usability, occupational performance and satisfaction evaluation of a smart environment controlled by infrared oculography by people with severe motor disabilities

--Manuscript Draft--

Manuscript Number:	PONE-D-20-12562R1
Article Type:	Research Article
Full Title:	Usability, occupational performance and satisfaction evaluation of a smart environment controlled by infrared oculography by people with severe motor disabilities
Short Title:	Evaluation of a smart environment controlled by infrared oculography
Corresponding Author:	Mariana Midori Sime, Ph.D.<br>Universidade Federal do Espirito Santo<br>Vitória, Espirito Santo BRAZIL
Keywords:	smart environment; smart home; people with disabilities; Assistive technology; infrared oculography; effectiveness
Abstract:	A smart environment is an assistive technology space that can enable people with motor disabilities to control their equipment (TV, radio, fan, etc.) through a human-machine interface activated by different inputs. However, assistive technology resources are not always considered useful, reaching quite high abandonment rate. This study aims to evaluate the effectiveness of a smart environment controlled through infrared oculography by people with severe motor disabilities. The study sample was composed of six individuals with motor disabilities Initially, sociodemographic data forms, the Functional Independence Measure (FIM TM), and the Canadian Occupational Performance Measure (COPM) were applied. The participants used the system in their domestic environment for a week. Afterwards, they were reevaluated with regards to occupational performance (COPM), satisfaction with the use of the assistive technology resource (QUEST 2.0), psychosocial impact (PIADS) and usability of the system (SUS), as well as through semi-structured interviews for suggestions or complaints. The most common demand from the participants of this research was ‘control of the TV’. Two participants did not use the system. All participants who used the system (four) presented positive results in all assessment protocols, evidencing greater independence in the control of the smart environment equipment. In addition, they evaluated the system as useful and with good usability. Non-acceptance of disability and lack of social support may have influenced the results.
Order of Authors:	Mariana Midori Sime, Ph.D.
	Alexandre Luís Cardoso Bissoli
	Daniel Lavino-Júnior
	Teodiano Freire Bastos-Filho
Response to Reviewers:	Response to Reviewers<br><br>Title: Usability, occupational performance and satisfaction evaluation of a smart environment controlled by infrared oculography by people with severe motor disabilities<br><br>In response to the reported pending issues, the following information is required:<br><br>-Point 1: The paper needs to be substantially shortened and condensed.<br>Answer to point 1: As recommended by the reviewer, the introduction and review have been combined and condensed. The description of the materials and instruments has been reduced. The results and discussion were separated and reorganized.<br><br>-Point 2: Details and photos that might allow to identify the participants should be omitted.<br>Answer to point 2: The vignettes with the details and photos of the participants have been removed and only the sociodemographic information that contributed to the discussion was maintained.

Powered by Editorial Manager® and ProduXion Manager® from Aries Systems Corporation

	-Point 3: About the funding received of the Google Inc.<br>Answer to point 3: Google Inc. only supported the research through Google's Latin America Research Awards to two authors, Teodiano Freire Bastos-Filho and Alexandre Luís Cardoso Bissoli, and this does not alter our adherence to Plos ONE policies on sharing data and materials. This statement can also be found in the cover letter, in the Competing Interests Statement section, as recommended<br>-Point 4: Please explain in more detail why "data cannot be shared publicly because of Ethics Committee terms".<br>Answer to point 4: Our data contained information that identified the participants. This information has been omitted and the data are being shared in the Supporting Information files.<br>- Point 5: Please amend the methods section and ethics statement of the manuscript to explicitly state that the patient/participant has provided consent for publication: "The individual in this manuscript has given written informed consent (as outlined in PLOS consent form) to publish these case details"<br>Ansewer to point 5: About the use of personal data, participants or their guardians signed the Free and Informed Consent Form (FICF), which guarantees that all personal data are confidential and private, even after the publication of the research. The data protection has been guaranteed by identifying participants by codes, instead of their names or initials, and by not including information such as date of birth and address. Detailed information about the participants health condition had already been excluded from the manuscript after the original decision letter. The terms with the highlighted subsection in both the original version in Portuguese and the translated version in English were included in the list of documents for submission. The current situation in Brazil, due to the COVID-19 pandemic is very serious, and it has been very difficult to meet the participants to obtain their signature on the PLOS specific consent form. Since they are people with disabilities and comorbidities, they have been in quarantine, as a measure to prevent spread of the Sars-CoV-2 virus. The methods section and ethics statement of the manuscript were amended to explicitly state that the participants has provided consent for publication, through the FICF.<br><br>Sincerely,<br>Mariana Midori Sime (e-mail: mariana.sime@ufes.br)<br>Vitória-ES (Brazil), May 01, 2021.
**Additional Information:**	
**Question**	**Response**
**Financial Disclosure**<br><br>Enter a financial disclosure statement that describes the sources of funding for the work included in this submission. Review the submission guidelines for detailed requirements. View published research articles from *PLOS ONE* for specific examples.<br><br>This statement is required for submission and **will appear in the published article** if the submission is accepted. Please make sure it is accurate.	AB and TBF received a award in Google Research Awards for Latin America, of Google Inc.<br>The funders had no role in study design, data collection and analysis, decision to publish, or preparation of the manuscript.

**Unfunded studies**<br>Enter: *The author(s) received no specific funding for this work.*<br><br>**Funded studies**<br>Enter a statement with the following details:<br>• Initials of the authors who received each award<br>• Grant numbers awarded to each author<br>• The full name of each funder<br>• URL of each funder website<br>• Did the sponsors or funders play any role in the study design, data collection and analysis, decision to publish, or preparation of the manuscript?<br>• **NO** - Include this sentence at the end of your statement: *The funders had no role in study design, data collection and analysis, decision to publish, or preparation of the manuscript.*<br>• **YES** - Specify the role(s) played.<br><br>* typeset	
**Competing Interests**<br><br>Use the instructions below to enter a competing interest statement for this submission. On behalf of all authors, disclose any competing interests that could be perceived to bias this work—acknowledging all financial support and any other relevant financial or non-financial competing interests.<br><br>This statement **will appear in the published article** if the submission is accepted. Please make sure it is accurate. View published research articles from *PLOS ONE* for specific examples.	Google Inc. only supported the research through Google's Latin America Research Awards (https://research.google/philosophy/) to two authors, Teodiano Freire Bastos-Filho and Alexandre Luís Cardoso Bissoli, and this does not alter our adherence to Plos ONE policies on sharing data and materials.

**NO authors have competing interests**<br><br>Enter: *The authors have declared that no competing interests exist.*<br><br>**Authors with competing interests**<br><br>Enter competing interest details beginning with this statement:<br><br>*I have read the journal's policy and the authors of this manuscript have the following competing interests: [insert competing interests here]*<br><br>* typeset	
**Ethics Statement**<br><br>Enter an ethics statement for this submission. This statement is required if the study involved:<br><br>• Human participants<br>• Human specimens or tissue<br>• Vertebrate animals or cephalopods<br>• Vertebrate embryos or tissues<br>• Field research<br><br>Write "N/A" if the submission does not require an ethics statement.<br><br>General guidance is provided below. Consult the submission guidelines for detailed instructions. **Make sure that all information entered here is included in the Methods section of the manuscript.**	This study was approved by the Human Research Ethics Committee of Federal University of Espirito Santo (UFES), Brazil, under protocol no. 976.828, CAAE 39410614.6.0000.5060. All participants or their legal guardians signed and received a copy of the Free and Informed Consent Form (FICF), allowing the publication of their data collected in the research, as long as the confidentiality of personal information is guaranteed.

Powered by Editorial Manager® and ProduXion Manager® from Aries Systems Corporation

**Format for specific study types**<br><br>**Human Subject Research (involving human participants and/or tissue)**<br>• Give the name of the institutional review board or ethics committee that approved the study<br>• Include the approval number and/or a statement indicating approval of this research<br>• Indicate the form of consent obtained (written/oral) or the reason that consent was not obtained (e.g. the data were analyzed anonymously)<br><br>**Animal Research (involving vertebrate animals, embryos or tissues)**<br>• Provide the name of the Institutional Animal Care and Use Committee (IACUC) or other relevant ethics board that reviewed the study protocol, and indicate whether they approved this research or granted a formal waiver of ethical approval<br>• Include an approval number if one was obtained<br>• If the study involved *non-human primates*, add *additional details* about animal welfare and steps taken to ameliorate suffering<br>• If anesthesia, euthanasia, or any kind of animal sacrifice is part of the study, include briefly which substances and/or methods were applied<br><br>**Field Research**<br><br>Include the following details if this study involves the collection of plant, animal, or other materials from a natural setting:<br>• Field permit number<br>• Name of the institution or relevant body that granted permission	
**Data Availability**<br><br>Authors are required to make all data underlying the findings described fully available, without restriction, and from the time of publication. PLOS allows rare exceptions to address legal and ethical concerns. See the PLOS Data Policy and FAQ for detailed information.	Yes - all data are fully available without restriction

A Data Availability Statement describing where the data can be found is required at submission. Your answers to this question constitute the Data Availability Statement and **will be published in the article**, if accepted.<br><br>**Important:** Stating 'data available on request from the author' is not sufficient. If your data are only available upon request, select 'No' for the first question and explain your exceptional situation in the text box.<br><br>Do the authors confirm that all data underlying the findings described in their manuscript are fully available without restriction?	
**Describe where the data may be found in full sentences. If you are copying our sample text, replace any instances of XXX with the appropriate details.**<br><br>• If the data are **held or will be held in a public repository**, include URLs, accession numbers or DOIs. If this information will only be available after acceptance, indicate this by ticking the box below. For example: *All XXX files are available from the XXX database (accession number(s) XXX, XXX.*).<br>• If the data are all contained **within the manuscript and/or Supporting Information files**, enter the following: *All relevant data are within the manuscript and its Supporting Information files.*<br>• If neither of these applies but you are able to provide **details of access elsewhere**, with or without limitations, please do so. For example:<br><br>*Data cannot be shared publicly because of [XXX]. Data are available from the XXX Institutional Data Access / Ethics Committee (contact via XXX) for researchers who meet the criteria for access to confidential data.*<br><br>*The data underlying the results presented in the study are available from (include the name of the third party*	All relevant data are within the manuscript and its Supporting Information files.

and contact information or URL).<br>• This text is appropriate if the data are owned by a third party and authors do not have permission to share the data.<br><br>* typeset	
Additional data availability information:	Tick here if your circumstances are not covered by the questions above and you need the journal's help to make your data available.

Powered by Editorial Manager® and ProduXion Manager® from Aries Systems Corporation

Manuscript
Click here to access/download;Manuscript;Manuscript.doc

Usability, occupational performance and satisfaction evaluation of a smart environment controlled by infrared oculography by people with severe motor disabilities

Mariana Midori Sime[1*¶,#a], Alexandre Luís Cardoso Bissoli[2&], Daniel Lavino-Júnior[3&], Teodiano Freire Bastos-Filho[4¶,#b]

[1] Occupational Therapy Department, Federal University of Espirito Santo (UFES), Vitoria, Brazil

[2] Postgraduate Program in Electrical Engineering, Electrical Engineering Department, Federal University of Espirito Santo (UFES), Vitoria, Brazil

[3] Electrical Engineering Department, Federal University of Espirito Santo (UFES), Vitoria, Brazil

[4] Postgraduate Program in Electrical Engineering, Electrical Engineering Department and Postgraduate Program in Biotechnology, Federal University of Espirito Santo (UFES), Vitoria, Brazil

[#a] Current Address: Department of Occupational Therapy, Federal University of Espirito Santo – Campus Maruipe, Vitoria, Brazil.

[#b] Current Address: Postgraduate Program in Electrical Engineering, Federal University of Espirito Santo – Campus Goiabeiras, Vitoria, Brazil.

* Corresponding author

E-mail: mariana.sime@ufes.br (MMS)

[¶] These authors contributed equally to this work.

[&] These authors also contributed equally to this work.

Abstract

A smart environment is an assistive technology space that can enable people with motor disabilities to control their equipment (TV, radio, fan, etc.) through a human-machine interface activated by different inputs. However, assistive technology resources are not always considered useful, reaching quite high abandonment rate. This study aims to evaluate the effectiveness of a smart environment controlled through infrared oculography by people with severe motor disabilities. The study sample was composed of six individuals with motor disabilities Initially, sociodemographic data forms, the Functional Independence Measure (FIM^{TM}), and the Canadian Occupational Performance Measure (COPM) were applied. The participants used the system in their domestic environment for a week. Afterwards, they were reevaluated with regards to occupational performance (COPM), satisfaction with the use of the assistive technology resource (QUEST 2.0), psychosocial impact (PIADS) and usability of the system (SUS), as well as through semi-structured interviews for suggestions or complaints. The most common demand from the participants of this research was ‘control of the TV’. Two participants did not use the system. All participants who used the system (four) presented positive results in all assessment protocols, evidencing greater independence in the control of the smart environment equipment. In addition, they evaluated the system as useful and with good usability. Non-acceptance of disability and lack of social support may have influenced the results.

Keywords: smart environment; smart home; people with disabilities; assistive technology; infrared oculography; effectiveness

Introduction

Assistive technology (AT) can be defined as an area of knowledge that includes products, resources, methodologies, strategies and services [1], or items, products and equipment acquired, adapted or modified [2], always with the aim of improving the functional performance, independence, and quality of life (QoL) of people with disabilities [1,2].

The literature indicates that individuals with diseases or injuries that affect the central nervous system, such as Multiple Sclerosis, Amyotrophic Lateral Sclerosis, Stroke, and Cranioencephalic or Spinal Cord Injury, can present sensory, motor, language and behavioral impairments at different levels, which lead to deficits in their occupational performance for carrying out Activities of Daily Living (ADL) independently, or interacting with people and objects [3–11], making them quite dependent on family members and/or caregivers [12].

According to the International Classification of Functioning, Disability and Health (ICF), people's impairments are configured as their environments/contexts limit their activities and restrict their social participation, not favoring their functionality [13–15].

The elements that constitute the ICF's model are Health Condition, Body Functions and Structures, and Activity, Participation, and Contextual Factors (Environmental and Personal), with AT devices and resources included in Environmental Factors [13–15], which improve the functionality of people with motor disabilities and/or older people, in different areas and health conditions [4,16–23].

However, although AT plays an important role in the recovery or improvement of the functionality of people with disabilities, the rates of abandonment and/or non-use of AT devices are high [25–28] for many reasons [19,23,24,28].

Conceptual models assist researchers and professionals with making better indications and implementation of AT devices. For instance, the Human Activity Assistive Technology (HAAT) model proposes to understand the role played by AT in the lives of people with disabilities. The HAAT model is based on four elements: the Human, the Activity, the Assistive Technology, and the Context in which the other three elements are inserted. It briefly describes "someone (human) doing something (activity) in a context using assistive technology" [29] (p.7).

Thus, during the process of preparing and/or indicating an AT resource or device, it is important to understand the activity that the person wants and needs to perform, the capacities they have, and the different aspects of the context that will influence their acquisition and use. Several studies have highlighted the importance of patient/user participation in the development of AT resources or devices [22,30–32], or in the process of defining and choosing the device that best suits their needs and of training and updating the team to evaluate and monitor the AT use [22,24,27,33].

Although there are several definitions of Smart Environment (SE) [34–36], it can be defined as a space (room, house, etc.) where services (temperature, lighting, entertainment, security, etc.) and/or equipment (lamps, home appliances, alarms, etc.) are managed intelligently using technology (personal computer, tablet, smartphone, remote control, etc.), through a Human-Machine Interface (HMI), aiming to assist users or residents with their ADL and provide them with better QoL [37,38].

Many studies have focused on the development of SEs that aim to provide greater independence for people with motor disabilities, combining their residual skills with the physical environment, since this group experiences several limitations in the use of environments and equipment control [37,39–45]. The secondary objective is to reduce their need for assistance from caregivers or family members [45].

Despite the gradual increase in the number of these studies, only few of them have addressed the benefits of SEs for people with disabilities regarding the exercise of autonomy, i.e., freedom of opinion, choice and decision [46], improvement of performance, and usability.

The reviews by Martin et al. [47] and Brandt et al. [48] found no evidence about the effectiveness of SEs for people with disabilities. Differences in sample size, interventions, and instruments used hinder comparison between these studies, but it was possible to notice a tendency to facilitate independence, instrumental ADL, socialization, and QoL.

Marikyan, Papagiannidis and Alamanos [49] consider that there is increased research addressing SEs; however, they are restricted to three themes: they ignore the multiple diversities of the concepts; focus on the functioning of technological devices, architecture and infrastructure; are little dedicated to the perspective of users.

For the control of electronic equipment in an SE by people with motor disabilities, different ways of capturing their inputs can be used. Among them, Infrared Oculography (IROG) is a technique that has been significantly studied in computer science [42,45,50–52].

In IROG, a device performs eye movement tracking, calculating the point on the computer screen the user is looking at. Eye tracking devices have a video camera equipped with high resolution infrared (IR) light-emitting diodes (LED) that reflect and increase the contrast between the pupil and the iris, allowing precise pupil location and facilitating the tracking of eye movement. This movement then functions as an HMI modality, enabling users to control several applications [53–55].

This technique has proved to be one of the most indicated and useful for people with severe motor disabilities, enabling them to use HMIs in an easier, comfortable and

intuitive way [56], without the need to place electrodes or equipment on their bodies. Another contributing factoris that eye movement is one of the few abilities maintained in people with severe motor disabilities [57].

Since the literature points to a lack of studies that address the effectiveness provided by AT [58,59] as well as the importance of good assessment using valid, reliable and viable instruments, and covering various resource aspects [60], it is important that further studies addressing the effectiveness of SEs in the everyday life of people with severe motor disabilities be conducted.

The SE system used in this study was developed at the Assistive Technology Center of the Federal University of Espirito Santo (UFES), Brazil. It consists of a smart global box (gBox) coupled to a computer software that enables the user to control the TV, radio, fan and/or lighting using eye-tracking technology [50].

In this sense, the main objective of this study was to evaluate the effectiveness of the developed SE controlled through IROG for specific use by people with severe motor disabilities.

Materials and Methods

This study was approved by the Human Research Ethics Committee of Federal University of Espirito Santo, Brazil, under protocol no. 976.828, CAAE 39410614.6.0000.5060. All participants or their legal guardians signed and received a copy of the Free and Informed Consent Form, allowing the publication of their data collected in the research, as long as the confidentiality of personal information is guaranteed.

Materials

The following materials were used in this study:

1. Notebook computer with Intel® Core™ i3-5005U processor, Windows® 10 Home Edition operating system, 4GB RAM, 500 GB HD memory, and 14” LED screen. 2. Tobii Eye Tracker 4C [61], which allows: a) booting with the computer, b) controlling with only one or both eyes, c) making movements with the head, maintaining the calibration. 3. Gaze Point software [62]: to control the mouse cursor using Tobii Eye Tracker 4C. 4. Global Box (gBox) (Fig 1): an SE controller module [50] that receives commands from the computer, via Wi-Fi, to activate home devices. 5. SE Control Interface (CI) (Fig 2) [50]: configured in a Web application in which it is possible to download the use data history, among other options. 6. Wi-Fi Router: to send the signal from the notebook computer to the gBox. 7. Portable table: used to position the equipment (Fig 3), facilitating its transport and use.

Fig 1. gBox: electronic module to control home devices in the SE.

Fig 2. User CI. Reproduced with permission from [50].

After clicking on START (a), the main menu (b) appears on the screen and the user can go to the icon associated with the device they wish to control (START), configure the system (CONFIG) (c), or return to the initial screen (CLOSE). To activate the devices (d), the mouse cursor must be positioned on desired icon for the time defined in the settings, then its background turns yellow (e), except for the TV icon, which has an individual submenu (f) to turn it on/off or control its channels or volume.

Fig 3. Portable table, eye tracker, and notebook computer are installed.

Data collection instruments

The following instruments were used to collect information about the participants during HMI use:

Sociodemographic data forms: used to collect the participants' personal data, information on the diagnosis and history of the disease or injury, and experience with technology.

Functional Independence Measure (FIM^{TM}) [63,64]: it assesses the degree of assistance needed for users to perform motor and cognitive ADL.

Canadian Occupational Performance Measure (COPM) [65]: it evaluates changes in the client's perception of their performance in activities and their satisfaction with them.

Psychosocial Impact of Assistive Devices Scale (PIADS) [66]: it assesses the effects of an AT device on the functional independence, well-being and QoL of users.

Quebec User Evaluation of Satisfaction with Assistive Technology (QUEST 2.0) [67,68]: it measures satisfaction with the AT resource and the service delivered.

System Usability Scale (SUS) [69]: it evaluates the usability of the environment control system.

Semi-structured interviews: they were audio recorded, carried out to obtain information about the process of using the system (positive and negative points, and suggestions).

Participants

Inclusion criteria: people aged ≥18 years with motor disabilities that compromised the normal interaction with equipment in the home environment, indicated by

rehabilitation institutions or professionals; they should also have a caregiver of age and literate. Exclusion criteria: individuals with cognitive deficits (related by their assisting professionals) that compromised understanding of the equipment functioning and use, as well as of the assessment instruments, and with visual deficits not corrected by glasses or contact lenses.

Procedures

A visit to each of the participant's homes was scheduled for the initial assessment and installation of the system. After acceptance, each participant or caregiver signed a FICF, responded to the sociodemographic data form and the FIM^{TM} and COPM measures, the last directed to activities that require the use of equipment.

The system was installed in the residence room most used during the day as indicated by the participant or caregiver (Figure 4).

Fig 4. System installed in the home of one of the study participants.

Both the participant and the caregiver were trained to use the system and received a copy of the user manual containing explanations on the eye tracker calibration and equipment control. The caregiver's role was to turn on the notebook computer, position the portable table, and calibrate the eye tracker whenever necessary.

The system remained installed at each participants' homes for one week, as in the study conducted by Calvo et al. [70]. When necessary, extra visits to the participants' homes were made in order to make adjustments or assist with use.

At the end of that period, reassessments were carried out using the COPM, the other instruments (QUEST 2.0, PIADS, and SUS), and the semi-structured interview.

It is worth mentioning that a pilot study was previously carried out with a participant with motor disabilities aiming to verify the system functionality in a common home environment, and whether the methodology was adequate to the study objectives.

The pre- and post-test statistical analyses of the COPM instrument were performed using the Paired Sample *t*-Test considering a statistically significant difference of 5% ($p<0.05$). This statistical test was chosen because of its robustness, considering the low sampling power obtained with small samples. Results of the other instruments are presented descriptively.

Results

Six people with disabilities participated in this study. Of these, two individuals did not use the system during the period that the equipment remained in their homes, and their cases will be presented and discussed separately.

Table 1 shows the main information about the participants who used the system.

Table 1. Data of participants who used the system

Participant	Gender	Type of caregiver	Health condition	Time elapsed since diagnosis
**PP1**	F[a]	Informal caregiver[c]	ALS[e] + Psoriatic Arthritis	5 months
**PP3**	F	Informal + formal caregiver[d]	Autoimmune vasculitis	8 years
**PP5**	M[b]	Informal caregiver	SCI[f] (incomplete C7 level)	29 years
**PP6**	M	Informal caregivers	ALS	1 year and 9 months

[a] F – Female; [b] M – Male; [c] Informal caregiver – refers to a family member who cares the person; [d] Formal caregiver – refers to a professionals who are paid to care; [e] ALS – Amyotrophic Lateral Sclerosis; [f] SCI – Spinal Cord Injury.

The mean age of participants was 49 years, ranging from 30 to 63 years. Participants PP1, PP5, and PP6 presented basic knowledge of technologies, more focused on the use of cell phones. Participant PP3, the youngest, had intermediate knowledge in using computers and cell phones before presenting the disease signs and symptoms.

Table 2 presents the results on functional independence collected through the FIM™.

Table 2. FIM™ results of participants who used the system

Participant	Motor FIM™	Cognitive FIM™	Total FIM™
**PP1**	47/91	35/35	82/126
**PP3**	25/91	23/35	48/126
**PP5**	44/91	35/35	79/126
**PP6**	67/91	35/35	102/126

As previously described, the FIM™ score considers the need for assistance in each activity. The participants' lower motor scores refer to difficulties in performing ADLs as well as in holding and manipulating objects used daily. Participant PP3 presented a lower cognitive score as a result of difficulty in communication.

In the COPM assessment, the participants were asked to indicate which activities were important in their everyday lives. Having 'control of the TV' was considered important by all participants, being able to 'turn the fan on/off' was deemed essential by two participants (PP5 and PP6), and having 'control of the lights' was a significant demand for only one of the participants (PP1).

At reassessment, participants PP1, PP5 and PP6 responded to the instruments with the help of the researcher to make markings on paper, and the interview was answered without help. With regard to participant PP3, the interview was conducted with her mother and, for the other evaluations, the scales were designed in the notebook computer and the participant indicate the alternative most appropriate moving the mouse cursor. The COPM was fully answered by the participant. The SUS and QUEST 2.0 were

answered jointly with the participant's mother. Due to tiredness, her mother responded to the PIADS based on what she believed her daughter's responses would be.

Results of the COPM and the Paired Sample *t*-Test for all participants are shown in Table 3.

Table 3. COPM and Paired Sample *t*-Test results of participants who used the system

Participant	Demands	Performance		Satisfaction		Change	
		P1[a]	P2[b]	S1[c]	S2[d]	P2 Total - P1 Total	S2 Total - S1 Total
**PP1**	Control of the TV	7	8	5	8		
	Control of the lights	6	7	5	8		
	Total score	6.5	7.5	5	8	1	3
**PP3**	Control of the TV	1	7	1	7		
	Total score	1	7	1	7	6	6
**PP5**	Control of the TV	1	10	5	10		
	Turn the fan on/off	1	10	1	10		
	Total score	1	10	3	10	9	7
**PP6**	Control of the TV	3	9	3	10		
	Turn the fan on/off	5	9	3	10		
	Total score	4	9	3	10	5	7
p*-value		Performance		Satisfaction			
**Control of the TV**		0.045		0.009			
**COPM total score**		0.050		0.009			

[a] P1- initial performance; [b] P2- final performance; [c] S1- initial satisfaction; [d] S2- final satisfaction. * Paired Sample *t*-Test ($p<0.05$).

For the Paired Sample *t*-Test, only the events 'control of the TV' and 'COPM total score' were analyzed, as these events were common to all participants.

Statistically significant results were observed for performance and satisfaction regarding ‘control of the TV’ and for total satisfaction after using the system. Borderline results were obtained with respect to total performance.

Table 4 shows the results obtained with application of the QUEST 2.0 instrument. The results are very close or equal to 5.0 (the highest possible score), corresponding to high levels of satisfaction.

Table 4. QUEST 2.0 scores

Participant	Resource	Service delivery	Total
**PP1**	4.5	5.0	4.7
**PP3**	4.4	5.0	4.6
**PP5**	5.0	5.0	5.0
**PP6**	5.0	5.0	5.0

Table 5 presents the items that the participants considered most important about the SE control system. Each participant should indicate three items, and ‘effectiveness’ was pointed by three of the four participants as an important feature of the SE tested.

Table 5. Important items regarding the SE system

Item	Number of citations
**Effectiveness**	3
**Adjustment**	2
**Simplicity of use**	2
**Professional services**	2
**Follow-up services**	1
**Comfort**	1
**Safety**	1

As for the PIADS instrument, Table 6 presents the score for each subscale and the final average of the participants, in which participants PP1, PP5, and PP6 were close to the maximum score (3.0).

Table 6. PIADS subscale scores

Participant	Competence	Adaptability	Self-esteem	Average
**PP1**	2.6	3.0	3.0	2.9
**PP3**	1.3	0.7	1.8	1.3
**PP5**	2.5	3.0	2.6	2.7
**PP6**	2.8	3.0	2.3	2.7

Figure 5 illustrates the results of the SUS, whose average score was 85.6.

Fig 5. SUS results of participants who used the system.

Through the interviews, all participants who used the SE system found it useful, mainly because it provided them with greater independence and exercise of autonomy in controlling the equipment, as it can be verified in some of their answers:

"Ah, it is useful in all aspects, right? Turn on, turn off" (PP1)

"I think it was good. I think (PP3) was happy to get it, right? You saw her expression of joy, right? So, it was (useful). The part that I found most positive is giving autonomy, right? This is fundamental!" (PP3's mother)

"Its ... accessibility to be able to move. (...) it was very useful ... with the difficulty that I have (...). The facility for you to pick up and do things" (PP5)

"It brings independence! Not depending on anybody to 'turn up the volume!', 'Switch channels!', or 'turn on the television!', 'turn off the television!'" (PP6)

As examples of difficulties or aspects that need to be improved in our system, the participants reported the delay to switch between distant TV channels; feeling tired or having a mild headache caused by the use of the eye tracker; dependence on a caregiver

or family member to start the system and open the CIs; complicated process for calibrating the eye tracker.

The following suggestions were made: a numeric keyboard to type the desired channel; remove the need to use the notebook computer keyboard for some tasks, such as login to CI; make the system simpler and more intuitive for people with little experience with computers; allow the system to also control all the home lighting of the residence and make and receive phone calls via a smartphone.

Regarding the user manual, participants PP1, PP5 and PP6 reported that they did not need to access it, because the explanation and training provided by the researchers were sufficient to use the SE system. Participant PP3's mother, on the other hand, reported that the manual did not clarify her doubts, requiring the presence of one of the researchers.

The system usage records, obtained through the Web application, enabled verification of the number of days that each participant effectively used the SE (Table 7).

Table 7. Usage registration information obtained through the Web application

Participant	Number of days of use
**PP1**	2
**PP3**	5
**PP5**	2
**PP6**	4

Table 8 shows the data of the participants who did not use the system.

Table 8. Data of participants who did not use the system

Participant	Gender	Type of caregiver	Health Condition	Time elapsed since diagnosis
PP2	F[a]	Formal caregivers[c]	Multiple Sclerosis	4 years
PP4	M[b]	Informal[d] + formal caregivers	SCI[e] (C5 level)	2 years

[a] F – Female; [b] M – Male; [c] Informal caregiver – refers to a family member who cares the person; [d] Formal caregiver – refers to a professionals who are paid to care; [e] SCI – Spinal Cord Injury.

At the initial assessment using the COPM, both PP2 and PP4 reported that watching TV was a very important activity in their everyday lives, but that they were not satisfied with the way they performed this activity. Thus, the TV was the only device connected to the gBox for control.

Table 9 presents the results of the FIM™ with respect to functional independence.

Table 9. FIM™ results of the participants who did not use the system

Participant	Motor FIM™	Cognitive FIM™	Total FIM™
**PP2**	26/91	35/35	61/126
**PP4**	13/91	35/35	48/126

According to the FIM™ data, both participants (PP2 and PP4) had need for maximum assistance to perform motor activities and presented total independence for cognitive activities.

At reassessment, these participants stated that they found the equipment useful, responding positively to all the assessment instruments. However, the system data records available at the Web application showed that they do not use the equipment at all.

Both participants present some similar characteristics that may have contributed to not using the equipment: they have difficulty dealing with the diagnosis or with their current health condition; caregivers not close or not engaged in this additional task; they report that the equipment does not allow total independence and that they have little knowledge of technology, limited to communication through the smartphone.

Discussion

This study was conducted with six participants. Despite its small sample size, this research aimed to analyze participants using the system in their homes, for a prolonged time, and not occasionally in the laboratory, because its main objectives were to assess occupational performance, usability, and satisfaction with the developed AT system.

All the participants who used the SE system need considerable assistance from their families or formal caregivers to perform their ADL, which was evidenced by the FIM^{TM}. For them, ‘control the TV’ was the most important activity, according to the COPM.

The TV is an extremely popular appliance and an important resource of entertainment, mainly for people with disabilities. According to Myburg et al. [71], TV control systems were among the most frequent environment control devices in the population studied (people with SCI).

Although the system presents more options to control electronic devices, the participants did not use all of them, according to the COPM. Several researchers have reported the significance of considering factors that are important for the people who will use an AT device [22,24,32] aiming at better adherence and results.

Two participants (PP1 and PP6) wear glasses. Duchowski [54] points out that the use of lenses (contact lenses or glasses) can interfere with the eye tracker ability to locate the corneal reflex, as they have reflective surfaces; however, the use of glasses did not interfere with the performance in using the system in the present study.

In the COPM, the participants self-evaluated their performance (P) in the activities they considered important, and their satisfaction (S) with performance before and after using the system. The higher the score, the better the performance or satisfaction.

The final assessments (P2 or S2) of all participants who used the system were higher than their initial assessments (P1 or S1). Except for the change in performance of

participant PP1, all other evaluations showed changes greater than two points, which is considered by Law et al. [65] as a clinically important change.

Statistical analysis of the COPM showed positive results. For participants PP3, PP5 and PP6, the initial evaluation scores indicate that they were unable to perform the activities or presented great difficulty in performing them, also reflecting on their low satisfaction. At the final assessment, the results clearly showed that the participants had a new way of interacting with the environment more actively and, consequently, greater satisfaction with performance.

Among all the participants, participant PP1 was the only one who still has some manual skills, thus she can operate the TV remote control, although with some difficulty, and getting tired along the process. Therefore, she presented higher initial scores and smaller changes at reevaluation.

Regarding the QUEST 2.0, to assess the satisfaction with the resource, the participants should consider the entire set of hardware (gBox, notebook computer, eye tracker, router, and portable table) and software (CI). Participants PP1 and PP3 scored less than 5.0, referring to difficulties in calibrating the eye tracker, occasional visual discomfort, and difficulty in using the system (in the case of the participant with the greatest motor impairment).

To assess the satisfaction with the service provided, the participants considered installation of the equipment, explanations, training, troubleshooting, and necessary follow-up during the week of use. In this item, all the scores were the highest (5.0). Good professionals and services are items appointed by Lenker et al. [22] as a positive point in the process of obtaining an AT resource, leading to better results with use. In contrast, lack of continuous support can lead participants to lose interest in its use [17].

The QUEST 2.0 total average (between 4.6 and 5.0) obtained in this research shows that the participants were satisfied with the SE. This result corroborates the findings of two studies of the systematic review conducted by Brandt et al. [48] on environmental control systems and smart homes used by people with disabilities.

The aspect that the participants considered most important in the SE control system was 'effectiveness'. Demers et al. [72] defined this term as the "goal achievement with the AT device" (p.189), reinforcing that the system has met the needs of these people. Our findings corroborate those by Shone Stickel et al. [73], who also found effectiveness as the most important attribute of electronic AT devices for performance of ADL.

In the PIADS, respondents assessed how they were affected by the SE system. Participants PP1, PP5, and PP6 had the highest average values, indicating a maximum positive impact with the use of the SE. They assigned the highest values to the Adaptability subscale, indicating that with the use of the system they felt more willing to take risks and more motivated to participate socially [66]. Participant PP3, who has the most significant motor impairment, presented the lowest average among the participants. This instrument, as previously mentioned, was answered by her mother based on what she believed her daughter's assessment would be. Thus, it may not reliably represent the participant's assessment.

The developers of this instrument [66] claim that these three subscales (Competence, Adaptability, and Self-esteem) are sufficiently sensitive to assess the psychosocial impact of an AT device or resource on the user, which are included in the QoL concept. In addition, the longer the period of use, the greater the feeling of competence [74], being that the hypotheses for it are that the longer the usage time: 1) the more the users appreciate the effect; 2) reflects the user's real need for the device.

Due to the short time of use of the SEs in this study (one week), it is not possible to state that there was a real change in the psychosocial aspects of the participants, but it indicates a tendency toward this change, in view of the results.

Regarding the SUS instrument, the result (85.6) indicates that the usability of our system was well evaluated. According to Bangor, Kortum and Miller [75], products evaluated in the range of 80 points are considered good, and products evaluated in the range of 90 points are considered exceptional.

The lowest evaluation refers, again, to participant PP3, whose answers pointed to some degree of complexity in the system and the need of a technical person or prior learning. Beyond the motor impairment of this participant, other factors may have interfered with the use of the eye tracker, such as her position in bed, small opening of the eye sometimes, tiredness with use, and difficulty of caregivers with the use of computers and programs.

Despite the lower ratings assigned by this participant, it seems that for all participants, on average, the assessment instruments showed positive results in relation to occupational performance, satisfaction with performance, satisfaction with the SE system, and usability of the system.

Many studies have evaluated improvements in these aspects after people with disabilities used environment control systems or electronic AT devices [17,44,73,76,77], whereas other studies have assessed ways of interacting with the environment through eye trackers [78,79]. However, no studies with the same objectives and using the same methodology of the present research, that is, use of IROG technology for SE control, have been found for comparison.

As the results show better occupational performance, satisfaction with performance and with the SE, and system usability, it can be concluded that the SE controlled by IROG

evaluated in this research provided people with motor disabilities with more independent operation and control of the equipment.

All the reports of participants point positive aspects with the use of the SE system. These statements corroborate the researched literature [80–83], since independence, control and privacy are highly important aspects pointed by people with disabilities who used environment control systems or electronic aids to daily living (EADL).

Participant PP6's speech also points to an outcome present in the study by Verdonck, Chard and Nolan's [81]: the embarrassment that people with disabilities present regarding their recurring requests for help, followed by apologies, as they feel uncomfortable to interrupt their caregivers' routine. According to those authors, the use of EADL changes this dynamic, with fewer apologies, less discomfort, and reduced caregiver burden.

The user manual was an additional material left with the participants to assist with the use of the SE system. The literature highlights how important explanations and training are for understanding the use and for adherence to the AT resource. Myburg et al. [71] found that training was considered crucial for the total integration of the environment control system in the lives of people with spinal cord injury, as well as the involvement of the occupational therapist in the testing, prescription and configuration of the system.

Information obtained through the Web application showed that the system was not used every day. The justifications given by the participants included trips, medical or rehabilitation consultations, and other leisure activities, such as going to church or taking short tours.

However, some other hypotheses were raised, corroborating the literature: the system has limitations, requiring other person to activate part of the equipment [82,84] or, when there is some voluntary movement, people prefer to behave as they are more accustomed

[85]. Another possibility is that the TV was controlled by a person who was in the same room as the participant, using the standard TV remote control.

About the participants who did not use the system, Costa et al. [85] found some factors that can contribute to the understanding: lack of equipment functionality (for not providing the desired independence), difficulty in use, embarrassment in using the device, lack of support from family members, lack of user motivation.

Regarding non-acceptance of diagnoses, studies have shown that this is an important factor to be considered when prescribing or selecting an AT device or resource [25,86]; however, in the present study, this information only appeared at the reevaluation.

Wessels et al. [25] reported that there is a difference in the way the AT resource will be viewed between people who were born with a disability (for them, technology opens a new range of possibilities) and those who have acquired a disability (because, for them, technology will never replace the lost function).

Participants PP2 and PP4 are in the second group, since they acquired the disability as adults. A recurring line in the interviews is that they were very active and independent in the past, and now they are dependent for practically all activities. For them, the disability has also brought other types of losses, such as moving from their hometowns, changing their standard of living, ending relationships, or losing jobs. Such cases often result in periods of depression [25]. In this sense, for these people, it is hypothesized that they would only benefit from technology if their dependency could be completely reversed.

Another associated factor that may have contributed to non-use of the SE is that the AT device can highlights the disability [19,23,86]. Verza et al. [86] found that 30.3% of the reasons for abandoning or not using an AT device are due to the patient's non-acceptance. For those authors, although the AT device is seen as a possibility to increase

functionality, it can be interpreted as a validation of the disability and loss of independence, resulting in decreased self-esteem.

It should be noted that, although the system registers activation of the equipment, this information was not passed on to the participants, so that the use of the system was based on their real needs and desires, and not on the fact that they felt obliged to use it.

It is worth restating that it is important that the professional involved perform a wide and in-depth assessment of the patient's real demands, expectations, and possibilities of the proposed AT device, as well as consider their participation in the choice. These points are important to ensure acceptance and continuity of use, since abandonment can represent a waste of time and financial expenses (their own or from the government) [17,24,26,27].

Conclusions

Participants who used the IROG-controlled SE system showed better occupational performance and satisfaction with performance. In addition, the psychosocial impact was close to the maximum, satisfaction with the system was well evaluated and, for three participants, the usability was considered good. The participant who had a more pronounced motor impairment and, therefore, more difficulty in using the system, rated its psychosocial impact and usability with lower scores.

The two participants who did not use the system presented characteristics such as non-acceptance of their diagnoses or current health conditions, weak relationship with caregivers, and need for assistance with using the equipment. Besides that, the fact that an AT device possibly reinforces disability may have corroborated these results.

The HAAT model was used as a basis for definition of the study methodology aiming to achieve its objectives. Although the AT resource (SE system) was previously defined, in order to be evaluated, the choice of participants was not random, and professionals were asked to indicate the possible participants. Regarding the Activity component, the activities considered important for each participant were evaluated according to their wishes, even though other possibilities are available for control. Still in the Activity component, it was considered the most familiar place to conduct the research, that is, each of the participant's homes.

Regarding the Human component, the motor and cognitive skills of the participant were taken into account. The eye tracker using IROG technology was choose as the most suitable technique, because is less intrusive. Motivation to resume performance of the activities was assessed through the COPM contemplating the most significant.

With respect to the Context component, it was noticed that the participants who had greater social support (from family members and/or formal caregivers) actually used the SE and were able to provide a more in-depth assessment. Those who did not have this support, or had it in a weakened way, did not use the SE.

Finally, regarding the Assistive Technology component, it was evaluated that the SE system functioned as a facilitator to carry out the desired activities, enabling greater exercise of autonomy and independence.

Concerning the ICF, its components can also be broken down into the elements of this research. Health Condition considered the severe motor disability of the participants. In Body Functions and Structures, the movement limitations interfered in the way each of them interacted with the electronic equipment. Activities important for the participants and their Participation were assessed through the FIM™ and COPM instruments. Personal Factors were verified using the QUEST 2.0 and PIADS

instruments. Finally, about Environmental Factors, the SE system, as in the HAAT model, is considered a facilitator, and its usability was assessed through the SUS scale.

Considering that disability is indicated by the HAAT model and the ICF as inherent in social structures, and not in the person, the results found suggest that the SE system enabled reduction in the incapacity of the participants, who thus had greater participation in related activities.

This study provided a wide evaluation of equipment that aims to allow greater independence for people with severe motor disabilities, from the point of view of its operation and usability, as well as the benefits it provided to the people who used it. Several assessment instruments were used to achieve the study objectives.

For professionals, this study highlights the importance of a good evaluation for the prescription and development of AT resources, as well as new possibilities to provide people with severe motor disabilities with greater independence to carry out their everyday activities with regard to equipment control, avoiding abandonment or non-use.

Future studies with larger samples and longer duration should be conducted, expanding the possibilities of controlled equipment and devices, in order to understand whether the benefits remain in long term.

Patents

The gBox patent, together with the environment control system named “Remote micro-controlled device for charging residential loads via the Internet with emitter and receiver of commands via integrated infrared”, was submitted to the Institute of Technological Innovation – INIT at UFES, Brazil, and evaluation is under process.

Acknowledgments

The authors would like to thank all the professionals and participants involved in this study for their contribution.

References

1. Brasil. CORDE. Comitê de Ajudas Técnicas, ATA VII; 2008. Available from: <http://www.mj.gov.br/sedh/ct/corde/dpdh/corde/comite_at.asp>.
2. United States Congress. Assistive Technology Act of 2004: Amendment to the assistive technology act of 1998 (Public Law 108–364); 2004.
3. Siqueira SC, Vitorino PVO, Prudente COM, Santana TS, Melo GF. Quality of life of patients with Amyotrophic Lateral Sclerosis. Rev Rene. 2017; 18(1):139-146. doi: 10.15253/2175-6783.2017000100019.
4. Marrie RA, Cutter GR, Tyry T, Cofield SS, Fox R, Salter A. Upper limb impairment is associated with use of assistive devices and unemployment in multiple sclerosis. Multiple Sclerosis and Related Disorders. 2017; 13:87-92. doi: 10.1016/j.msard.2017.02.013.
5. Shamshiri H, Fatehi F, Abolfazli R, Harirchian MH, Sedighi B, Zamani B, et al. Trends of quality of life changes in amyotrophic lateral sclerosis patients. J Neurol Sci. 2016; 368:35-40. doi: 10.1016/j.jns.2016.06.056.
6. Bertoni R, Lamers I, Chen CC, Feys P, Cattaneo D. Unilateral and bilateral upper limb dysfunction at body functions, activity and participation levels in people with multiple sclerosis. Mult Scler. 2015; 21(12):1566-1574. doi: 10.1177/1352458514567553.
7. Souza FDA, Cruz DMC, Ferrigno ISV, Tsukimoto GR, Figliolia CS. Correlação entre papéis ocupacionais e independência de usuários com lesão medular em

processo de reabilitação. Mundo da Saúde. 2013; 37(2):166-175. doi: 10.15343/0104-7809.2013372166175.

8. Riazi A, Bradshaw SA, Playford ED. Quality of life in the care home: a qualitative study of the perspectives of residents with multiple sclerosis. Disabil Rehabil. 2012; 34(24):2095-2102. doi: 10.3109/09638288.2012.672539.
9. Lee YC, Chen YM, Hsueh IP, Wang YH, Hsieh CL. The impact of stroke: insights from patients in Taiwan. Occup Ther Int. 2010; 17(3):152-158. doi: 10.1002/oti.301.
10. Wolf TJ, Baum C, Connor LT. Changing face of stroke: Implications for occupational therapy practice. AJOT. 2009; 63:621-625. doi: 10.5014/ajot.63.5.621.
11. Pedretti LW, Early ME. Desempenho ocupacional e modelos de prática para disfunção física. In. Pedretti LW, Early ME. Terapia ocupacional: capacidades práticas para disfunções físicas. São Paulo: Roca; 2004.
12. Camacho A, Esteban J, Paradas C. Informe de la Fundación del Cerebro sobre el impacto social de la esclerosis lateral amiotrófica y las enfermedades neuromusculares. Neurología. 2015; 33:35-46. doi: 10.1016/j.nrl.2015.02.003.
13. World Health Organization – WHO. International Classification of Functioning, Disability and Health: ICF. World Health Organization; 2001.
14. Farias N, Buchalla CM. A Classificação Internacional de Funcionalidade, Incapacidade e Saúde da Organização Mundial da Saúde: conceitos, usos e perspectivas. Rev Bras Epidemiol. 2005; 8(2):187-193. doi: 10.1590/S1415-790X2005000200011.

15. Dahl TH. International classification of functioning, disability and health: an introduction and discussion of its potential impact on rehabilitation services and research. J Rehabil Med. 2002; 34:201-204. doi: 10.1080/165019702760279170.

16. Borg J, Lindström A, Larsson S. Assistive technology in developing countries: national and international responsibilities to implement the Convention on the Rights of Persons with Disabilities. Viewpoint. Lancet. 2009; 374(28):1863-1865. doi: 10.1016/S0140-6736(09)61872-9.

17. Jamwal R, Callaway L, Ackerl J, Farnworth L, Winkler D. Electronic assistive technology used by people with acquired brain injury in shared supported accommodation: Implications for occupational therapy. British Journal of Occupational Therapy. 2017; 80(2):89-98. doi: 10.1177/0308022616678634.

18. Cruz DMC, Ioshimoto MTA. Tecnologia assistiva para as atividades de vida diária na tetraplegia completa C6 pós-lesão medular. Rev Triang: Ens. Pesq. Ext. Uberaba. 2010; 3(2):177-190. doi: 10.18554/rt.v3i2.153.

19. Andrade VS, Pereira LSM. Influência da tecnologia assistiva no desempenho funcional e na qualidade de vida de idosos comunitários frágeis: uma revisão bibliográfica. Rev Bras Geriatr Gerontol. 2009; 12(1):113-122. doi: 10.1590/1809-9823.2009120110.

20. Plotkin A, Sela L, Weissbrod A, Kahana R, Haviv L, Yeshurun Y, et al. Sniffing enables communication and environmental control for the severely disabled. PNAS. 2010; 107(32):14413-14418. doi: 10.1073/pnas.1006746107.

21. Lulé D, Zickler C, Häcker S, Bruno MA, Demertzi A, Pellas F, et al. Life can be worth living in locked-in syndrome. Progress in Brain Research. 2009; 177:339-351. doi: 10.1016/S0079-6123(09)17723-3.

22. Lenker JA, Harris F, Taugher M, Smith RO. Consumer perspectives on assistive technology outcomes. Disabil Rehabil Assist Technol. 2013; 8(5):373-380. doi: 10.3109/17483107.2012.749429.

23. Squires LA, Williams N, Morrison VL. Matching and accepting assistive technology in multiple sclerosis: A focus group study with people with multiple sclerosis, carers and occupational therapists. Journal of Health Psychology. 2016; 24:1-15. doi: 10.1177/1359105316677293.

24. Cruz DMC, Emmel MLG, Manzini MG, Mendes PVB. Assistive technology accessibility and abandonment: challenges for occupational therapists. The Open Journal of Occupational Therapy. 2016; 4(1):Article 10. doi: 10.15453/2168-6408.1166.

25. Wessels R, Dijcks B, Soede M, Gelderblom GJ, De Witte L. Non-use of provided assistive technology devices: a literature overview. Technology and Disability. 2003; 15(4):231-238. doi: 10.3233/TAD-2003-15404.

26. Riemer-Reiss ML, Wacker RR. Factors associated with assistive technology discontinuance among individuals with disabilities. Journal of Rehabilitation. 2000; 66(3):44-50.

27. Phillips B, Zhao H. Predictors of assistive technology abandonment. Assistive Technology. 1993; 5(1):36-45. doi: 10.1080/10400435.1993.10132205.

28. Alper S, Raharinirina S. Assistive technology for individuals with disabilities: a review and synthesis of the literature. Journal of Special Education Technology. 2006; 21(2):47-64. doi: 10.1177/016264340602100204.

29. Cook A, Polgar J. Assistive Technologies: Principles and Practice. 4th ed. St. Louis: Mosby Elselvier; 2015.

30. Kinney A, Goodwin DM, Gitlow L. Measuring assistive technology outcomes: a user centered approach. Assistive Technology Outcomes and Benefits. 2016; 10:94-110.

31. Peterson DB, Murray GC. Ethics and assistive technology service provision. Disabil Rehabil Assist Technol. 2006; 1:59-67. doi: 10.1080/09638280500167241.

32. Rocha EF, Castiglioni MC. Reflexões sobre recursos tecnológicos: ajudas técnicas, tecnologia assistiva, tecnologia de assistência e tecnologia de apoio. Rev Ter Ocup Univ São Paulo. 2005; 16(3):97-104. doi: 10.11606/issn.2238-6149.v16i3p97-104.

33. Netten JJ, Dijkstra PU, Geertzen JHB, Postema K. What influences a patient's decision to use custom-made orthopaedic shoes? BMC Musculoskeletal Disorders. 2012; 13:92. doi: 10.1186/1471-2474-13-92.

34. Rampinelli M, Bastos TF, Vassallo RF, Pizarro D. Implementation of an intelligent space for localizing and controlling a robotic wheelchair. In: 2012 ISSNIP Biosignals and Biorobotics Conference: Biosignals and Robotics for Better and Safer Living (BRC); 9-11th January 2012; Manaus-Brazil: IEEE – Institute of Electrical and Electronics Engineers; 2012. 1-4. doi: 10.1109/BRC.2012.6222187.

35. Lee JH, Hashimoto H. Intelligent Space: concept and contents. Advanced Robotics. 2002; 16(3):265-280. doi: 10.1163/156855302760121936.

36. Aarts EH, Encarnação JL. Into Ambient Intelligence. In. Aarts EH, Encarnação JL, editors. True visions: The emergence of ambient intelligence. Springer: Netherlands; 2008. pp. 1-16.

37. Kadam R, Mahamuni P, Parikh Y. Smart Home System. International Journal of Innovative Research in Advanced Engineering. 2015; 2:81-86.

38. Rivera-Illingworth F, Callaghan V, Hagras H. A neural network agent based approach to activity detection in AmI environments. In: Proceedings of IEEE

International Workshop on Intelligent Environments; 28-29th June 2005; Colchester (United Kingdom). Institution of Engineering and Technology; 2005. 1-12. doi: 10.1049/ic:20050222.

39. Tello RJMG, Bissoli ALC, Ferrara F, Müller S, Ferreira A, Bastos-Filho TF. Development of a Human Machine Interface for Control of Robotic Wheelchair and Smart Environment. IFAC-PapersOnLine. 2015; 48:136-141. doi: 10.1016/j.ifacol.2015.12.023.

40. Hussein A, Adda M, Atieh M, Fahs W. Smart Home Design for Disabled People based on Neural Networks. Procedia Computer Science. 2014; 37:117-126. doi: 10.1016/j.procs.2014.08.020.

41. Rampinelli M, Covre VB, Queiroz FM, Vassallo RF, Bastos-Filho TF, Mazo M. An intelligent space for mobile robot localization using a multi-camera system. Sensors. 2014; 14:15039-15064. doi: 10.3390/s140815039.

42. Adami I, Antona M, Stephanidis C. Ambient Assisted Living for the Motor Impaired. In. Koroupetroglou G, editor. Assistive Technologies, Disability Informatics and Computer Access for Motor Limitations. USA: IGI Global; 2013, pp. 76-104.

43. Bastos Filho TF, Fernandes MR, Lucena-Jr VF, Pereira CE. Proposal of Architecture for Integration of a Wheelchair in an Intelligent Space. In: Proceedings of 4th IEEE Biosignals and Biorobotics Conference (ISSNIP); 18-20th February 2013; Rio de Janeiro-RJ (Brazil); 2013. 6-11.

44. Ocepek J, Roberts AEK, Vidmar G. Evaluation of treatment in the smart home IRIS in terms of functional independence and occupational performance and satisfaction. Computational and Mathematical Methods in Medicine. 2013; 2013:10 pages. doi: 10.1155/2013/926858.

45. Gentry T. Smart homes for people with neurological disability: State of the art. NeuroRehabilitation. 2009; 25:209-217. doi: 10.3233/NRE-2009-0517.

46. Muñoz D, Fortes P. O princípio da autonomia e o consentimento livre e esclarecido. In. Iniciação à Bioética. Costa SIF, Oselka G, Garrafa V, coordinators. Brasília: CFM; 1998. pp. 53-70.

47. Martin S, Kelly G, Kernohan WG, McCreight B, Nugent C. Smart home technologies for health and social support. Cochrane Database of Systematic Reviews. 2008; 4:1-10. doi: 10.1002/14651858.CD006412.pub2.

48. Brandt Å, Samuelsson K, Töytäri O, Salminen A-L. Activity and participation, quality of life and user satisfaction outcomes of environmental control systems and smart home technology: a systematic review. Disability and Rehabilitation: Assistive Technology. 2011; 6(3):189-206. doi: 10.3109/17483107.2010.532286.

49. Marikyan D, Papagiannidis S, Alamanos E. A systematic review of the smart home literature: A user perspective. Technological Forecasting & Social Change. 2019; 138:139-154. doi: 10.1016/j.techfore.2018.08.015.

50. Bissoli A, Lavino-Junior D, Sime M, Encarnação L, Bastos-Filho T. A Human-Machine Interface Based on Eye Tracking for Controlling and Monitoring a Smart Home Using the Internet of Things. Sensors. 2019; 19:859-884. doi: 10.3390/s19040859.

51. Ding Z, Luo J, Deng H. Accelerated exhaustive eye glints localization method for infrared video oculography. In: SAC '18 – Proceedings of the 33rd Annual ACM Symposium on Applied Computing; April 2018; Pau France. New York-NY (United States of America): Association for Computing Machinery; 2018. 620–627. doi: https://doi.org/10.1145/3167132.3167200.

52. Eid MA, Giakoumidis N, El-Saddik A. A Novel Eye-Gaze-Controlled Wheelchair System for Navigating Unknown Environments: Case Study with a Person with ALS. IEEE Access. 2016; 4:558-573. doi:10.1109/ACCESS.2016.2520093.
53. Giannotto EC. Uso de rastreamento do olhar na avaliação da experiência do tele-usuário de aplicações de TV interativa. M.Sc. Thesis, Polytechnic School of the University of São Paulo. Department of Computer Engineering and Digital Systems. 2009, 290 p. Available from: https://teses.usp.br/teses/disponiveis/3/3141/tde-15042009-151212/pt-br.php.
54. Duchowski AT. Eye tracking methodology: Theory and practice. 2nd ed. London: Springer-Verlag; 2007.
55. Anders S, Weiskopf N, Lule D, Birbaumer N. Infrared oculography—validation of a new method to monitor startle eyeblink amplitudes during fMRI. NeuroImage. 2004; 22(2):767-770. doi: 10.1016/j.neuroimage.2004.01.024.
56. Sharma A, Abrol P. Eye gaze techniques for human computer interaction: a research survey. International Journal of Computer Applications. 2013; 71(9):18-29. doi: 10.5120/12386-8738.
57. Van Es MA, Hardiman O, Chio A, Al-Chalabi A, Pasterkamp RJ, Veldink JH, et al. Amyotrophic lateral sclerosis. The Lancet. 2017; 390(10107):2084-2098. doi: 10.1016/S0140-6736(17)31287-4.
58. Alves ACJ, Matsukura TS. Revisão sobre avaliações para indicação de dispositivos de tecnologia assistiva. Rev Ter Ocup Univ São Paulo. 2014; 25(2):199-207. doi: 10.11606/issn.2238-6149.v25i2p199-207.
59. Lovarini M, Mccluskey A, Curtin M. Editorial: Critically appraised papers. Limited high-quality research in the effectiveness of assistive technology. Aust Occup Ther J. 2006; 50(1):50.

60. Gelderblom GJ, De Witte LP. The assessment of assistive technology outcomes, effects and costs. Technol and Disabil. 2002; 14(3):91-94. doi: 10.3233/tad-2002-14302.

61. Tobii Eye Tracker 4C. Available from: https://gaming.tobii.com/product/tobii-eye-tracker-4c/. Access in: February 24, 2019.

62. Tobii Dynavox. Gaze Point. Available from: https://www.tobiidynavox.com/en-us/software/free-resources/gaze-point-1/. Acess in: February 24, 2019.

63. Riberto M, Miyazaki MH, Jucá SSH, Sakamoto H, Pinto PPN, Battistella LR. Validação da Versão Brasileira da Medida de Independência Funcional. Acta Fisiatr. 2004; 11(2):72-76. doi: 10.5935/0104-7795.20040003.

64. Linacre JM, Heinemann AW, Wright BD, Granger CV, Hamilton BB. The structure and stability of the Functional Independence Measure. Arch Phys Med Rahabil. 1994; 75:127-132. doi: 10.1016/0003-9993(94)90384-0.

65. Law M, Baptiste S, Carswell A, McColl M, Polatajko H, Pollock N. Medida canadense de desempenho ocupacional (COPM). Magalhães LC, Magalhães, LV, Cardoso AA, organizers and translators. Belo Horizonte: Ed UFMG; 2009.

66. Jutai J, Day H. Psychosocial Impact of Assistive Devices Scale (PIADS). Technol and Disabil. 2002; 14:107-111. doi: 10.1037/t45599-000.

67. Demers L, Weiss-Lambrou R, Ska B. The Quebec User Evaluation of Satisfaction with Assistive Technology (QUEST 2.0): an overview and recent progress. Technol and Disabil. 2002; 14:101-105. doi: 10.13072/midss.298.

68. Carvalho KEC, Gois Junior MB, Sá KN. Tradução e validação do Quebec User Evaluation of Satisfaction with Assistive Technology (QUEST 2.0) para o idioma português do Brasil. Rev. Bras. Reumatol. 2014; 54(4):260-267. doi: 10.1016/j.rbr.2014.04.003.

69. Brooke J. SUS: a "quick and dirty" usability scale. In: Jordan PW, Thomas B, McClelland IL, Weerdmeester B, editors. Usability Evaluation in Industry. London: Taylor and Francis; 1996. 189-194.

70. Calvo A, Chiò A, Castellina E, Corno F, Farinetti L, Ghiglione P, et al. Eye tracking impact on Quality-of-life of ALS patients. In: Computers Helping People with Special Needs. Proceedings of 11th International conference on computers helping people with special needs; 9-11th July 2008; Linz (Austria): Springer-Verlag Berlin Heidelberg; 2008. 70-77. doi: 10.1007/978-3-540-70540-6.

71. Myburg M, Allan E, Nalder E, Schuurs S, Amsters D. Environmental control systems – the experiences of people with spinal cord injury and the implications for prescribers. Disability and Rehabilitation: Assistive Technology. 2017; 12(2):128-136. doi: 10.3109/17483107.2015.1099748.

72. Demers L, Wessels R, Weiss-Lambrou R, Ska B, de Witte LP. Key dimensions of client satisfaction with assistive technology: a cross-validation of a Canadian measure in the Netherlands. J Rehabil Med. 2001; 33:187-191. doi: 10.1080/165019701750300663.

73. Shone SM, Ryan S, Rigby PJ, Jutai JW. Toward a comprehensive evaluation of the impact of electronic aids to daily living: evaluation of consumer satisfaction. Disabil Rehabil. 2002; 24:115-125. doi: 10.1080/09638280110066794.

74. Day H, Jutai J. Measuring the psychosocial impact of assistive devices: the PIADS. Canadian Journal of Rehabilitation. 1996; 9(2):159-168.

75. Bangor A, Kortum P, Miller JA. The System Usability Scale (SUS): An Empirical Evaluation. International Journal of Human-Computer Interaction. 2008; 24(6):574-594. doi: 10.1080/10447310802205776.

76. Little R. Electronic aids for daily living. Phys Med Rehabil Clin N Am. 2010; 21:33-42. doi: 10.1016/j.pmr.2009.07.008.
77. Boman I-L, Tham K, Granqvist A, Bartfai A, Hemmingsson H. Using electronic aids to daily living after acquired brain injury: A study of the learning process and the usability. Disability and Rehabilitation: Assistive Technology. 2007; 2(1):23-33. doi: 10.1080/17483100600856213.
78. Boustany G, Itani AED, Youssef R, Chami O, Abu-Faraj ZO. Design and development of a rehabilitative eye-tracking based home automation system. In: Proceedings of 2016 3rd Middle East Conference on Biomedical Engineering (MECBME); 6-7th October 2016; Beirut (Lebanon). Institute of Electrical and Electronics Engineers (IEEE): Curran Associates, Inc.; Jan 2017. 30-33. doi: 10.1109/MECBME.2016.7745401.
79. Brennan CP, McCullagh PJ, Galway L, Lightbody G. Promoting autonomy in a smart home environment with a smarter interface. In: Proceedings of 2015 37th Annual International Conference of the IEEE Engineering in Medicine and Biology Society (EMBC); August 25-29 2015; MiCo – Milano Conference Center, Milan (Italy). Institute of Electrical and Electronics Engineers (IEEE): Curran Associates, Inc.; Dec 2015. 5032-5035. doi: 10.1109/EMBC.2015.7319522.
80. Hooper B, Verdonck M, Amsters D, Myburg M, Allan E. Smart-device environmental control systems: experiences of people with cervical spinal cord injuries. Disability and Rehabilitation: Assistive Technology. 2018; 13(8):24-730. doi: 10.1080/17483107.2017.1369591.
81. Verdonck MC, Chard G, Nolan M. Electronic aids to daily living: be able to do what you want. Disability and Rehabilitation: Assistive Technology. 2010; 6(3):268-281. doi:10.3109/17483107.2010.525291.

82. Judge S, Robertson Z, Hawley MS. Users' Perceptions of Environmental Control Systems. In: Emiliani PL, Burzagli L, Como A, Gabbanini F, Salminen A-L (eds.). Assistive Technology from Adapted Equipment to Inclusive Environments - AAATE 2009; August 31st–September 2nd 2009; Florence (Italy). Amsterdam: IOS Press; 2009. 426-431.

83. Harmer J, Bakheit AMO. The benefits of environmental control systems as perceived by disabled users and their carers. British Journal of Occupational Therapy. 1999; 62(9):394-398. doi: 10.1177/030802269906200902.

84. Palmer P, Seale J. Exploring the attitudes to environmental control systems of people with physical disabilities: a grounded theory approach. Technol Disabil. 2007; 9(1):17-27. doi: 10.3233/TAD-2007-19103.

85. Costa CR, Ferreira FMRM, Bortolus MV, Carvalho MGR. Dispositivos de tecnologia assistiva: fatores relacionados ao abandono. Cad. Ter. Ocup. UFSCar. 2015; 23(3):611-624. doi: 10.4322/0104-4931.ctoAR0544.7.8.

86. Verza R, Carvalho ML, Battaglia MA, Uccelli MM. An interdisciplinary approach to evaluating the need for assistive technology reduces equipment abandonment. Mult Scler. 2006; 12(1):88-93. doi: 10.1191/1352458506ms1233oa.

Figure 1

Click here to access/download;Figure;Fig 1.tif

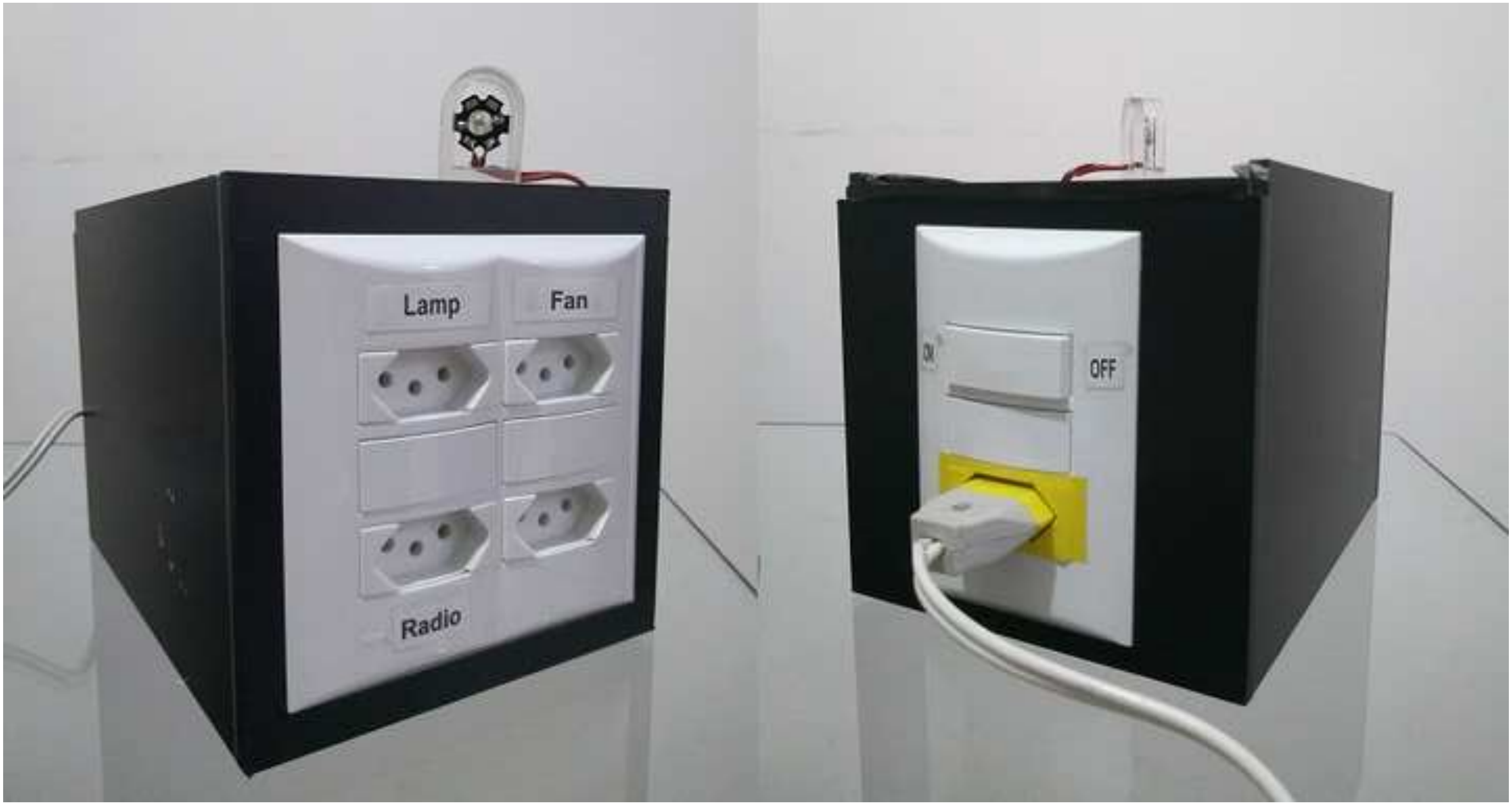

Figure 2

Click here to access/download;Figure;Fig 2.tif

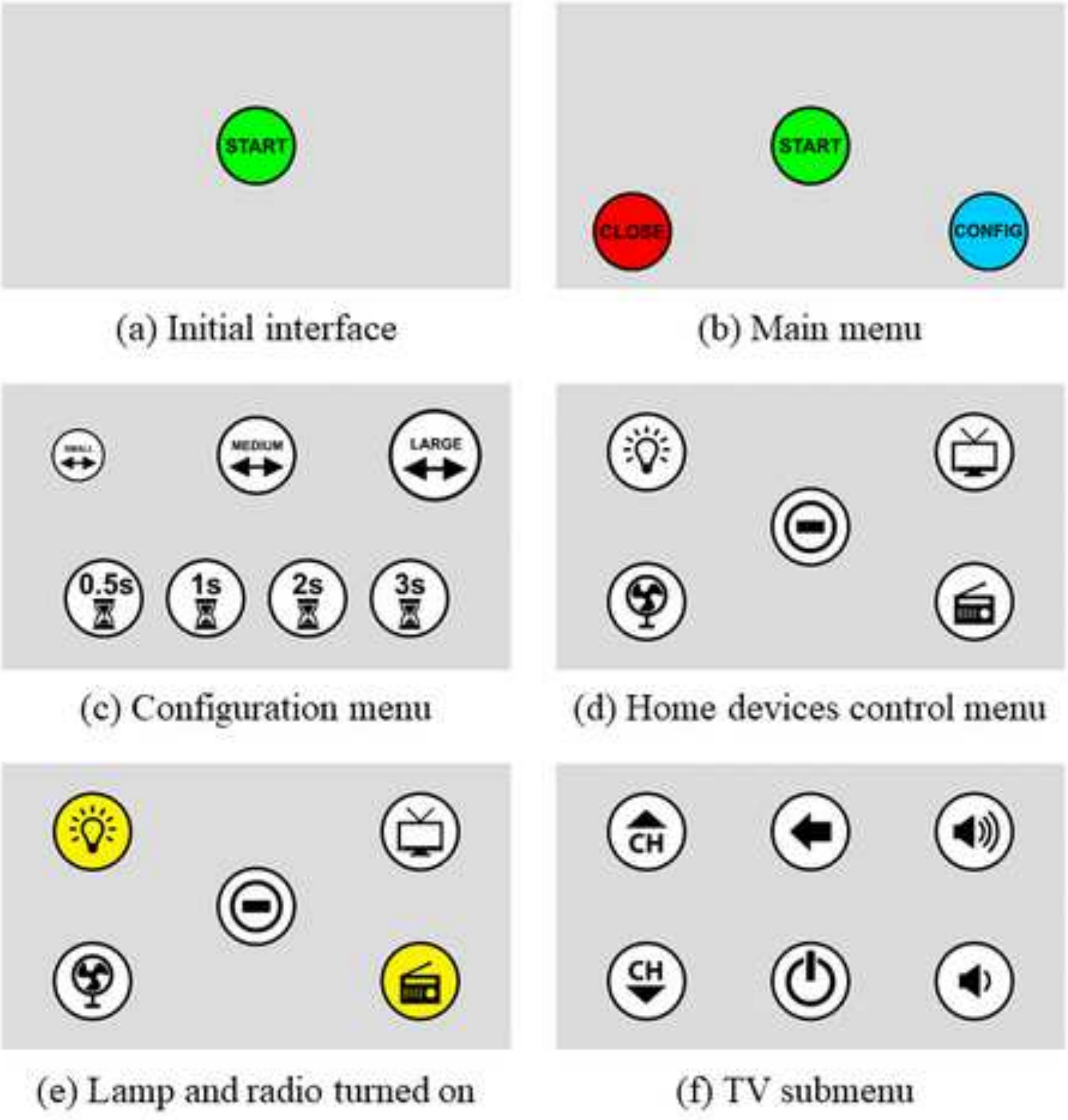

(a) Initial interface
(b) Main menu
(c) Configuration menu
(d) Home devices control menu
(e) Lamp and radio turned on
(f) TV submenu

Figure 5

Click here to access/download;Figure;Fig 5.tif

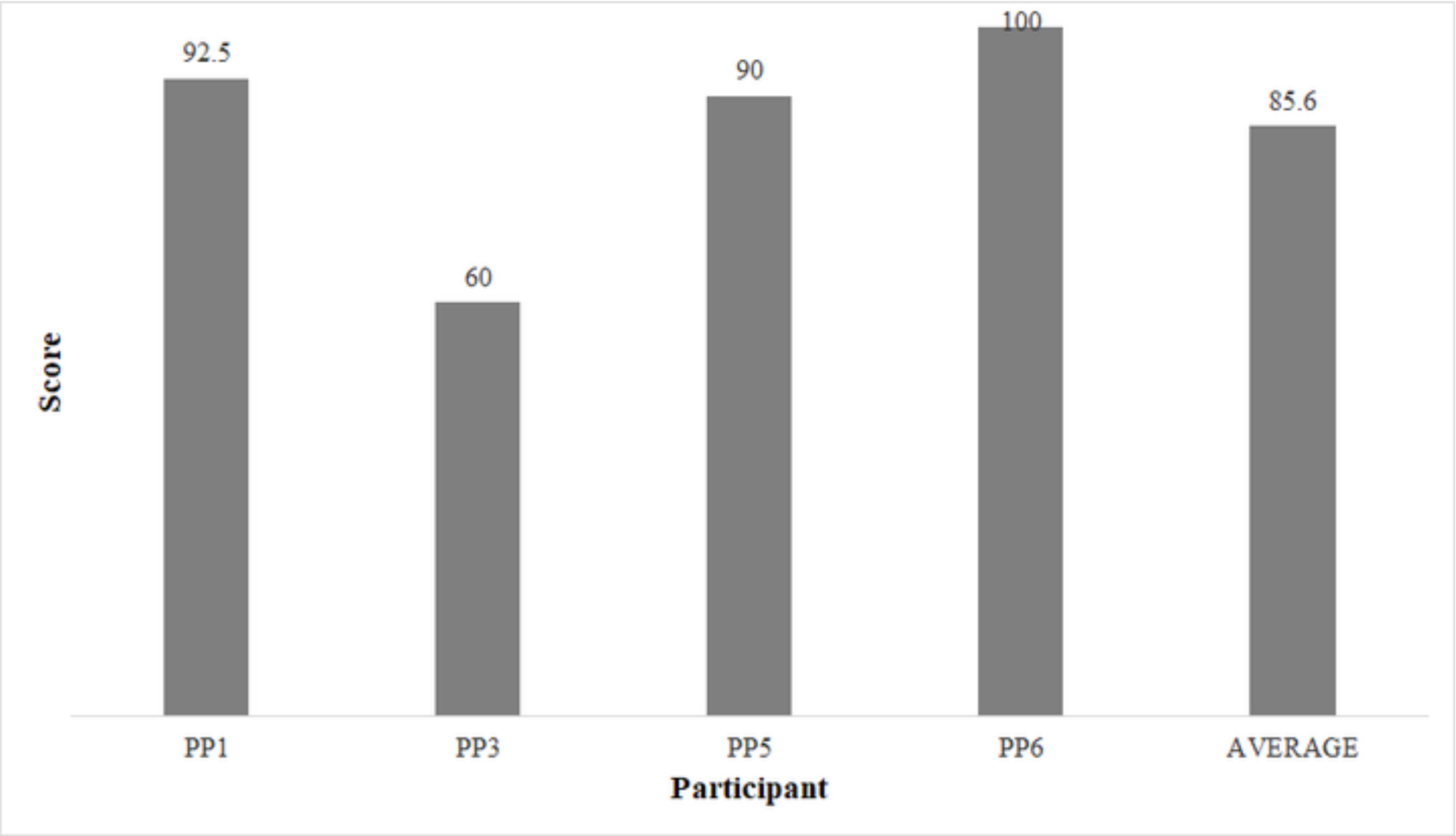

Figure 3

Click here to access/download;Figure;Fig 3.tif

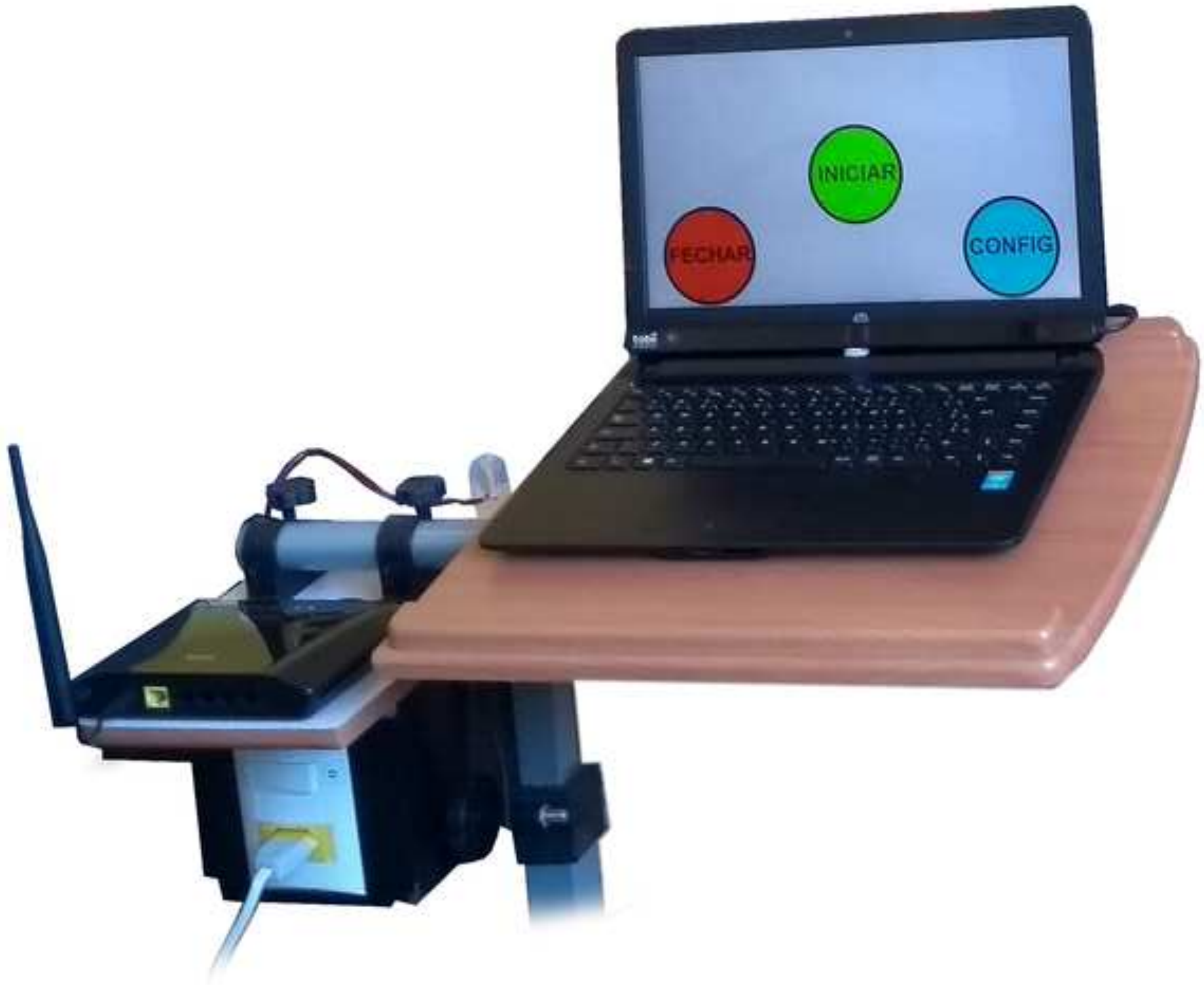

Figure 4

Click here to access/download;Figure;Fig 4.tif

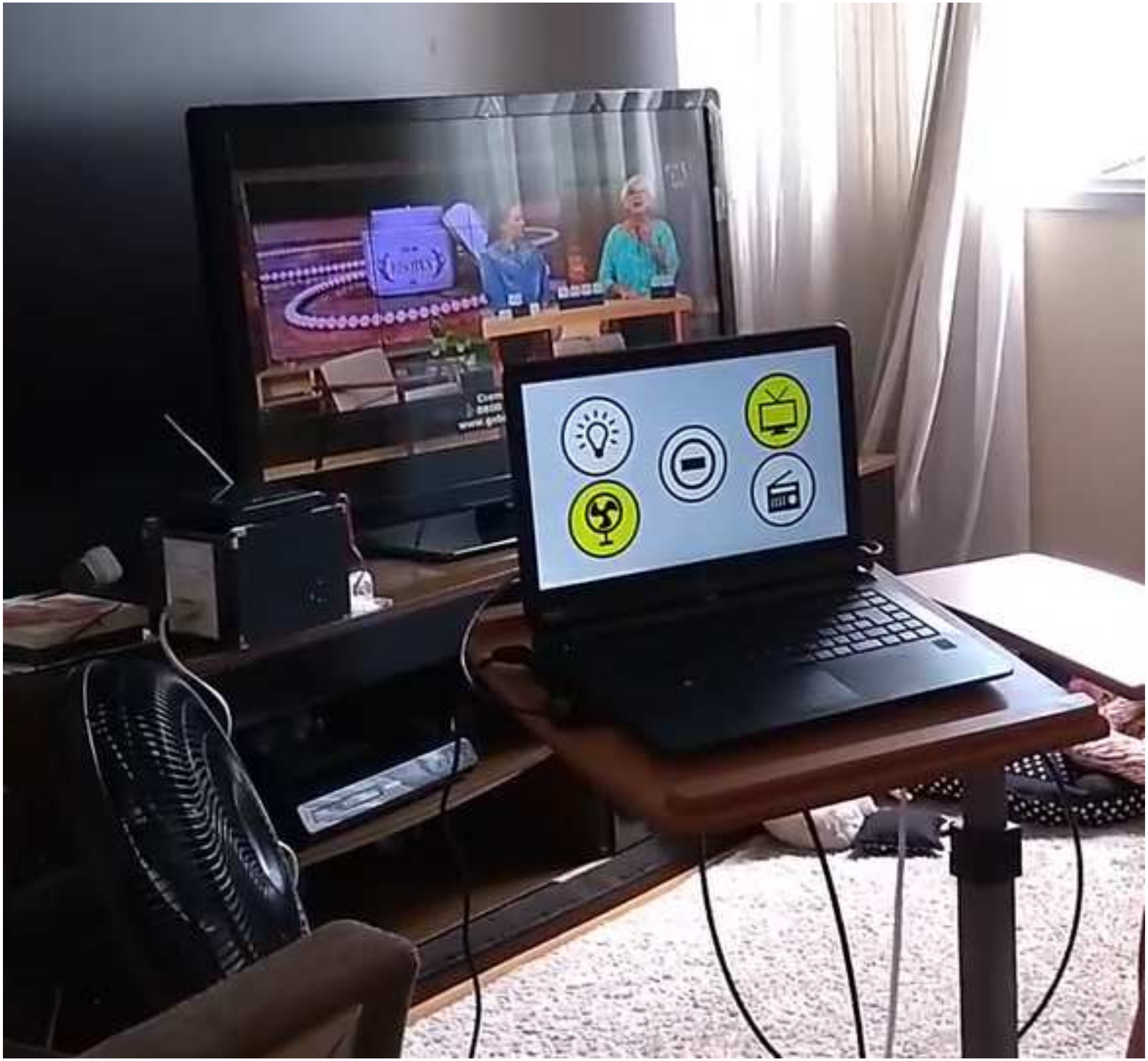

Click here to access/download
Supporting Information
Minimal Data Set.xls

Usability, occupational performance and satisfaction evaluation of a smart environment controlled by infrared oculography by people with severe motor disabilities

Mariana Midori Sime[1*¶,#a], Alexandre Luís Cardoso Bissoli[2&], Daniel Lavino-Júnior[3&], Teodiano Freire Bastos-Filho[4¶,#b]

[1] Occupational Therapy Department, Federal University of Espirito Santo (UFES), Vitoria, Brazil

[2] Postgraduate Program in Electrical Engineering, Electrical Engineering Department, Federal University of Espirito Santo (UFES), Vitoria, Brazil

[3] Electrical Engineering Department, Federal University of Espirito Santo (UFES), Vitoria, Brazil

[4] Postgraduate Program in Electrical Engineering, Electrical Engineering Department and Postgraduate Program in Biotechnology, Federal University of Espirito Santo (UFES), Vitoria, Brazil

[#a] Current Address: Department of Occupational Therapy, Federal University of Espirito Santo – Campus Maruipe, Vitoria, Brazil.

[#b] Current Address: Postgraduate Program in Electrical Engineering, Federal University of Espirito Santo – Campus Goiabeiras, Vitoria, Brazil.

* Corresponding author

E-mail: mariana.sime@ufes.br (MMS)

[¶] These authors contributed equally to this work.

[&] These authors also contributed equally to this work.

Abstract

A smart environment is an assistive technology space that can enable people with motor disabilities to control their equipment (TV, radio, fan, etc.) through a human-machine interface activated by different inputs. However, assistive technology resources are not always considered useful, reaching quite high abandonment rate. This study aims to evaluate the effectiveness of a smart environment controlled through infrared oculography by people with severe motor disabilities. The study sample was composed of six individuals with motor disabilities Initially, sociodemographic data forms, the Functional Independence Measure (FIM™), and the Canadian Occupational Performance Measure (COPM) were applied. The participants used the system in their domestic environment for a week. Afterwards, they were reevaluated with regards to occupational performance (COPM), satisfaction with the use of the assistive technology resource (QUEST 2.0), psychosocial impact (PIADS) and usability of the system (SUS), as well as through semi-structured interviews for suggestions or complaints. The most common demand from the participants of this research was ‘control of the TV’. Two participants did not use the system. All participants who used the system (four) presented positive results in all assessment protocols, evidencing greater independence in the control of the smart environment equipment. In addition, they evaluated the system as useful and with good usability. Non-acceptance of disability and lack of social support may have influenced the results.

Keywords: smart environment; smart home; people with disabilities; assistive technology; infrared oculography; effectiveness

Introduction

Assistive technology (AT) can be defined as an area of knowledge that includes products, resources, methodologies, strategies and services [1], or items, products and equipment acquired, adapted or modified [2], always with the aim of improving the functional performance, independence, and quality of life (QoL) of people with disabilities [1,2].

The literature indicates that individuals with diseases or injuries that affect the central nervous system, such as Multiple Sclerosis, Amyotrophic Lateral Sclerosis, Stroke, and Cranioencephalic or Spinal Cord Injury, can present sensory, motor, language and behavioral impairments at different levels, which lead to deficits in their occupational performance for carrying out Activities of Daily Living (ADL) independently, or interacting with people and objects [3–11], making them quite dependent on family members and/or caregivers [12].

According to the International Classification of Functioning, Disability and Health (ICF), people's impairments are configured as their environments/contexts limit their activities and restrict their social participation, not favoring their functionality [13–15].

The elements that constitute the ICF's model are Health Condition, Body Functions and Structures, and Activity, Participation, and Contextual Factors (Environmental and Personal), with AT devices and resources included in Environmental Factors [13–15], which improve the functionality of people with motor disabilities and/or older people, in different areas and health conditions [4,16–23].

However, although AT plays an important role in the recovery or improvement of the functionality of people with disabilities, the rates of abandonment and/or non-use of AT devices are high [25–28] for many reasons [19,23,24,28].

Conceptual models assist researchers and professionals with making better indications and implementation of AT devices. For instance, the Human Activity Assistive Technology (HAAT) model proposes to understand the role played by AT in the lives of people with disabilities. The HAAT model is based on four elements: the Human, the Activity, the Assistive Technology, and the Context in which the other three elements are inserted. It briefly describes “someone (human) doing something (activity) in a context using assistive technology” [29] (p.7).

Thus, during the process of preparing and/or indicating an AT resource or device, it is important to understand the activity that the person wants and needs to perform, the capacities they have, and the different aspects of the context that will influence their acquisition and use. Several studies have highlighted the importance of patient/user participation in the development of AT resources or devices [22,30–32], or in the process of defining and choosing the device that best suits their needs and of training and updating the team to evaluate and monitor the AT use [22,24,27,33].

Although there are several definitions of Smart Environment (SE) [34–36], it can be defined as a space (room, house, etc.) where services (temperature, lighting, entertainment, security, etc.) and/or equipment (lamps, home appliances, alarms, etc.) are managed intelligently using technology (personal computer, tablet, smartphone, remote control, etc.), through a Human-Machine Interface (HMI), aiming to assist users or residents with their ADL and provide them with better QoL [37,38].

Many studies have focused on the development of SEs that aim to provide greater independence for people with motor disabilities, combining their residual skills with the physical environment, since this group experiences several limitations in the use of environments and equipment control [37,39–45]. The secondary objective is to reduce their need for assistance from caregivers or family members [45].

Despite the gradual increase in the number of these studies, only few of them have addressed the benefits of SEs for people with disabilities regarding the exercise of autonomy, i.e., freedom of opinion, choice and decision [46], improvement of performance, and usability.

The reviews by Martin et al. [47] and Brandt et al. [48] found no evidence about the effectiveness of SEs for people with disabilities. Differences in sample size, interventions, and instruments used hinder comparison between these studies, but it was possible to notice a tendency to facilitate independence, instrumental ADL, socialization, and QoL.

Marikyan, Papagiannidis and Alamanos [49] consider that there is increased research addressing SEs; however, they are restricted to three themes: they ignore the multiple diversities of the concepts; focus on the functioning of technological devices, architecture and infrastructure; and are little dedicated to the perspective of users.

For the control of electronic equipment in an SE by people with motor disabilities, different ways of capturing their inputs can be used. Among them, Infrared Oculography (IROG) is a technique that has been significantly studied in computer science [42,45,50–52].

In IROG, a device performs eye movement tracking, calculating the point on the computer screen the user is looking at. Eye tracking devices have a video camera equipped with high resolution infrared (IR) light-emitting diodes (LED) that reflect and increase the contrast between the pupil and the iris, allowing precise pupil location and facilitating the tracking of eye movement. This movement then functions as an HMI modality, enabling users to control several applications [53–55].

This technique has proved to be one of the most indicated and useful for people with severe motor disabilities, enabling them to use HMIs in an easier, comfortable and

intuitive way [56], without the need to place electrodes or equipment on their bodies. Another contributing ~~factoris~~ factor is that eye movement is one of the few abilities maintained in people with severe motor disabilities [57].

Since the literature points to a lack of studies that address the effectiveness provided by AT [58,59] as well as the importance of good assessment using valid, reliable and viable instruments, and covering various resource aspects [60], it is important that further studies addressing the effectiveness of SEs in the everyday life of people with severe motor disabilities be conducted.

The SE system used in this study was developed at the Assistive Technology Center of the Federal University of Espirito Santo (UFES), Brazil. It consists of a smart global box (gBox) coupled to a computer software that enables the user to control the TV, radio, fan and/or lighting using eye-tracking technology [50].

In this sense, the main objective of this study was to evaluate the effectiveness of the developed SE controlled through IROG for specific use by people with severe motor disabilities.

Materials and Methods

This study was approved by the Human Research Ethics Committee of Federal University of Espirito Santo, Brazil, under protocol no. 976.828, CAAE 39410614.6.0000.5060. All participants or their legal guardians signed and received a copy of the Free and Informed Consent Form, allowing the publication of their data collected in the research, as long as the confidentiality of personal information is guaranteed.

Materials

The following materials were used in this study:

1. Notebook computer with Intel® Core™ i3-5005U processor, Windows® 10 Home Edition operating system, 4GB RAM, 500 GB HD memory, and 14” LED screen. 2. Tobii Eye Tracker 4C [61], which allows: a) booting with the computer, b) controlling with only one or both eyes, c) making movements with the head, maintaining the calibration. 3. Gaze Point software [62]: to control the mouse cursor using Tobii Eye Tracker 4C. 4. Global Box (gBox) (Fig 1): an SE controller module [50] that receives commands from the computer, via Wi-Fi, to activate home devices. 5. SE Control Interface (CI) (Fig 2) [50]: configured in a Web application in which it is possible to download the use data history, among other options. 6. Wi-Fi Router: to send the signal from the notebook computer to the gBox. 7. Portable table: used to position the equipment (Fig 3), facilitating its transport and use.

Fig 1. gBox: electronic module to control home devices in the SE.

Fig 2. User CI. Reproduced with permission from [50].

After clicking on START (a), the main menu (b) appears on the screen and the user can go to the icon associated with the device they wish to control (START), configure the system (CONFIG) (c), or return to the initial screen (CLOSE). To activate the devices (d), the mouse cursor must be positioned on desired icon for the time defined in the settings, then its background turns yellow (e), except for the TV icon, which has an individual submenu (f) to turn it on/off or control its channels or volume.

Fig 3. Portable table, eye tracker, and notebook computer are installed.

Data collection instruments

The following instruments were used to collect information about the participants during HMI use:

Sociodemographic data forms: used to collect the participants' personal data, information on the diagnosis and history of the disease or injury, and experience with technology.

Functional Independence Measure (FIM^{TM}) [63,64]: it assesses the degree of assistance needed for users to perform motor and cognitive ADL.

Canadian Occupational Performance Measure (COPM) [65]: it evaluates changes in the client's perception of their performance in activities and their satisfaction with them.

Psychosocial Impact of Assistive Devices Scale (PIADS) [66]: it assesses the effects of an AT device on the functional independence, well-being and QoL of users.

Quebec User Evaluation of Satisfaction with Assistive Technology (QUEST 2.0) [67,68]: it measures satisfaction with the AT resource and the service delivered.

System Usability Scale (SUS) [69]: it evaluates the usability of the environment control system.

Semi-structured interviews: they were audio recorded, carried out to obtain information about the process of using the system (positive and negative points, and suggestions).

Participants

Inclusion criteria: people aged ≥18 years with motor disabilities that compromised the normal interaction with equipment in the home environment, indicated by

rehabilitation institutions or professionals; they should also have a caregiver of age and literate. Exclusion criteria: individuals with cognitive deficits (related by their assisting professionals) that compromised understanding of the equipment functioning and use, as well as of the assessment instruments, and with visual deficits not corrected by glasses or contact lenses.

Procedures

A visit to each of the participant's homes was scheduled for the initial assessment and installation of the system. After acceptance, each participant or caregiver signed a FICF, responded to the sociodemographic data form and the FIM^{TM} and COPM measures, the last directed to activities that require the use of equipment.

The system was installed in the residence room most used during the day as indicated by the participant or caregiver (Figure 4).

Fig 4. System installed in the home of one of the study participants.

Both the participant and the caregiver were trained to use the system and received a copy of the user manual containing explanations on the eye tracker calibration and equipment control. The caregiver's role was to turn on the notebook computer, position the portable table, and calibrate the eye tracker whenever necessary.

The system remained installed at each participants' homes for one week, as in the study conducted by Calvo et al. [70]. When necessary, extra visits to the participants' homes were made in order to make adjustments or assist with use.

At the end of that period, reassessments were carried out using the COPM, the other instruments (QUEST 2.0, PIADS, and SUS), and the semi-structured interview.

It is worth mentioning that a pilot study was previously carried out with a participant with motor disabilities aiming to verify the system functionality in a common home environment, and whether the methodology was adequate to the study objectives.

The pre- and post-test statistical analyses of the COPM instrument were performed using the Paired Sample *t*-Test considering a statistically significant difference of 5% (p<0.05). This statistical test was chosen because of its robustness, considering the low sampling power obtained with small samples. Results of the other instruments are presented descriptively.

Results

Six people with disabilities participated in this study. Of these, two individuals did not use the system during the period that the equipment remained in their homes, and their cases will be presented and discussed separately.

Table 1 shows the main information about the participants who used the system.

Table 1. Data of participants who used the system

Participant	Gender	Type of caregiver	Health condition	Time elapsed since diagnosis
**PP1**	F[a]	Informal caregiver[c]	ALS[e] + Psoriatic Arthritis	5 months
**PP3**	F	Informal + formal caregiver[d]	Autoimmune vasculitis	8 years
**PP5**	M[b]	Informal caregiver	SCI[f] (incomplete C7 level)	29 years
**PP6**	M	Informal caregivers	ALS	1 year and 9 months

[a] F – Female; [b] M – Male; [c] Informal caregiver – refers to a family member who cares the person; [d] Formal caregiver – refers to a professionals who are paid to care; [e] ALS – Amyotrophic Lateral Sclerosis; [f] SCI – Spinal Cord Injury.

The mean age of participants was 49 years, ranging from 30 to 63 years. Participants PP1, PP5, and PP6 presented basic knowledge of technologies, more focused on the use of cell phones. Participant PP3, the youngest, had intermediate knowledge in using computers and cell phones before presenting the disease signs and symptoms.

Table 2 presents the results on functional independence collected through the FIM™.

Table 2. FIM™ results of participants who used the system

Participant	Motor FIM™	Cognitive FIM™	Total FIM™
**PP1**	47/91	35/35	82/126
**PP3**	25/91	23/35	48/126
**PP5**	44/91	35/35	79/126
**PP6**	67/91	35/35	102/126

As previously described, the FIM™ score considers the need for assistance in each activity. The participants' lower motor scores refer to difficulties in performing ADLs as well as in holding and manipulating objects used daily. Participant PP3 presented a lower cognitive score as a result of difficulty in communication.

In the COPM assessment, the participants were asked to indicate which activities were important in their everyday lives. Having 'control of the TV' was considered important by all participants, being able to 'turn the fan on/off' was deemed essential by two participants (PP5 and PP6), and having 'control of the lights' was a significant demand for only one of the participants (PP1).

At reassessment, participants PP1, PP5 and PP6 responded to the instruments with the help of the researcher to make markings on paper, and the interview was answered without help. With regard to participant PP3, the interview was conducted with her mother and, for the other evaluations, the scales were designed in the notebook computer and the participant indicate the ~~alternative~~ most appropriate moving the mouse cursor. The COPM was fully answered by the participant. The SUS and QUEST 2.0 were

answered jointly with the participant's mother. Due to ~~tiredness,~~ her mother responded to the PIADS based on what she believed her daughter's responses would be.

Results of the COPM and the Paired Sample *t*-Test for all participants are shown in Table 3.

Table 3. COPM and Paired Sample *t*-Test results of participants who used the system

Participant	Demands	Performance		Satisfaction		Change	
		P1[a]	P2[b]	S1[c]	S2[d]	P2 Total - P1 Total	S2 Total - S1 Total
**PP1**	Control of the TV	7	8	5	8		
	Control of the lights	6	7	5	8		
	Total score	6.5	7.5	5	8	1	3
**PP3**	Control of the TV	1	7	1	7		
	Total score	1	7	1	7	6	6
**PP5**	Control of the TV	1	10	5	10		
	Turn the fan on/off	1	10	1	10		
	Total score	1	10	3	10	9	7
**PP6**	Control of the TV	3	9	3	10		
	Turn the fan on/off	5	9	3	10		
	Total score	4	9	3	10	5	7
p*-value		Performance		Satisfaction			
**Control of the TV**		0.045		0.009			
**COPM total score**		0.050		0.009			

[a] P1- initial performance; [b] P2- final performance; [c] S1- initial satisfaction; [d] S2- final satisfaction. * Paired Sample *t*-Test (p<0.05).

For the Paired Sample *t*-Test, only the events 'control of the TV' and 'COPM total score' were analyzed, as these events were common to all participants.

Statistically significant results were observed for performance and satisfaction regarding ‘control of the TV’ and for total satisfaction after using the system. Borderline results were obtained with respect to total performance.

Table 4 shows the results obtained with application of the QUEST 2.0 instrument. The results are very close or equal to 5.0 (the highest possible score), corresponding to high levels of satisfaction.

Table 4. QUEST 2.0 scores

Participant	Resource	Service delivery	Total
**PP1**	4.5	5.0	4.7
**PP3**	4.4	5.0	4.6
**PP5**	5.0	5.0	5.0
**PP6**	5.0	5.0	5.0

Table 5 presents the items that the participants considered most important about the SE control system. Each participant should indicate three items, and ‘effectiveness’ was pointed by three of the four participants as an important feature of the SE tested.

Table 5. Important items regarding the SE system

Item	Number of citations
**Effectiveness**	3
**Adjustment**	2
**Simplicity of use**	2
**Professional services**	2
**Follow-up services**	1
**Comfort**	1
**Safety**	1

As for the PIADS instrument, Table 6 presents the score for each subscale and the final average of the participants, in which participants PP1, PP5, and PP6 were close to the maximum score (3.0).

Table 6. PIADS subscale scores

Participant	Competence	Adaptability	Self-esteem	Average
**PP1**	2.6	3.0	3.0	2.9
**PP3**	1.3	0.7	1.8	1.3
**PP5**	2.5	3.0	2.6	2.7
**PP6**	2.8	3.0	2.3	2.7

Figure 5 illustrates the results of the SUS, whose average score was 85.6.

Fig 5. SUS results of participants who used the system.

Through the interviews, all participants who used the SE system found it useful, mainly because it provided them with greater independence and exercise of autonomy in controlling the equipment, as it can be verified in some of their answers:

"Ah, it is useful in all aspects, right? Turn on, turn off" (PP1)

"I think it was good. I think (PP3) was happy to get it, right? You saw her expression of joy, right? So, it was (useful). The part that I found most positive is giving autonomy, right? This is fundamental!" (PP3's mother)

"Its ... accessibility to be able to move. (...) it was very useful ... with the difficulty that I have (...). The facility for you to pick up and do things" (PP5)

"It brings independence! Not depending on anybody to 'turn up the volume!', 'Switch channels!', or 'turn on the television!', 'turn off the television!'" (PP6)

As examples of difficulties or aspects that need to be improved in our system, the participants reported the delay to switch between distant TV channels; feeling tired or having a mild headache caused by the use of the eye tracker; dependence on a caregiver

or family member to start the system and open the CIs; complicated process for calibrating the eye tracker.

The following suggestions were made: a numeric keyboard to type the desired channel; remove the need to use the notebook computer keyboard for some tasks, such as login to CI; make the system simpler and more intuitive for people with little experience with computers; allow the system to also control all the home lighting of the residence and make and receive phone calls via a smartphone.

Regarding the user manual, participants PP1, PP5 and PP6 reported that they did not need to access it, because the explanation and training provided by the researchers were sufficient to use the SE system. Participant PP3's mother, on the other hand, reported that the manual did not clarify her doubts, requiring the presence of one of the researchers.

The system usage records, obtained through the Web application, enabled verification of the number of days that each participant effectively used the SE (Table 7).

Table 7. Usage registration information obtained through the Web application

Participant	Number of days of use
**PP1**	2
**PP3**	5
**PP5**	2
**PP6**	4

Table 8 shows the data of the participants who did not use the system.

Table 8. Data of participants who did not use the system

Participant	Gender	Type of caregiver	Health Condition	Time elapsed since diagnosis
PP2	F[a]	Formal caregivers[c]	Multiple Sclerosis	4 years
PP4	M[b]	Informal[d] + formal caregivers	SCI[e] (C5 level)	2 years

[a] F – Female; [b] M – Male; [c] Informal caregiver – refers to a family member who cares the person; [d] Formal caregiver – refers to a professionals who are paid to care; [e] SCI – Spinal Cord Injury.

At the initial assessment using the COPM, both PP2 and PP4 reported that watching TV was a very important activity in their everyday lives, but that they were not satisfied with the way they performed this activity. Thus, the TV was the only device connected to the gBox for control.

Table 9 presents the results of the FIM™ with respect to functional independence.

Table 9. FIM™ results of the participants who did not use the system

Participant	Motor FIM™	Cognitive FIM™	Total FIM™
**PP2**	26/91	35/35	61/126
**PP4**	13/91	35/35	48/126

According to the FIM™ data, both participants (PP2 and PP4) had need for maximum assistance to perform motor activities and presented total independence for cognitive activities.

At reassessment, these participants stated that they found the equipment useful, responding positively to all the assessment instruments. However, the system data records available at the Web application showed that they do not use the equipment at all.

Both participants present some similar characteristics that may have contributed to not using the equipment: they have difficulty dealing with the diagnosis or with their current health condition; caregivers not close or not engaged in this additional task; they report that the equipment does not allow total independence and that they have little knowledge of technology, limited to communication through the smartphone.

Discussion

This study was conducted with six participants. Despite its small sample size, this research aimed to analyze participants using the system in their homes, for a prolonged time, and not occasionally in the laboratory, because its main objectives were to assess occupational performance, usability, and satisfaction with the developed AT system.

All the participants who used the SE system need considerable assistance from their families or formal caregivers to perform their ADL, which was evidenced by the FIM™. For them, 'control the TV' was the most important activity, according to the COPM.

The TV is an extremely popular appliance and an important resource of entertainment, ~~mainly for people with disabilities.~~ According to Myburg et al. [71], TV control systems were among the most frequent environment control devices in the population studied (people with SCI).

Although the system presents more options to control electronic devices, the participants did not use all of them, according to the COPM. Several researchers have reported the significance of considering factors that are important for the people who will use an AT device [22,24,32] aiming at better adherence and results.

Two participants (PP1 and PP6) wear glasses. Duchowski [54] points out that the use of lenses (contact lenses or glasses) can interfere with the eye tracker ability to locate the corneal reflex, as they have reflective surfaces; however, the use of glasses did not interfere with the performance in using the system in the present study.

In the COPM, the participants self-evaluated their performance (P) in the activities they considered important, and their satisfaction (S) with performance before and after using the system. The higher the score, the better the performance or satisfaction.

The final assessments (P2 or S2) of all participants who used the system were higher than their initial assessments (P1 or S1). Except for the change in performance of

participant PP1, all other evaluations showed changes greater than two points, which is considered by Law et al. [65] as a clinically important change.

Statistical analysis of the COPM showed positive results. For participants PP3, PP5 and PP6, the initial evaluation scores indicate that they were unable to perform the activities or presented great difficulty in performing them, also reflecting on their low satisfaction. At the final assessment, the results clearly showed that the participants had a new way of interacting with the environment more actively and, consequently, greater satisfaction with performance.

Among all the participants, participant PP1 was the only one who still has some manual skills, thus she can operate the TV remote control, although with some difficulty, and getting tired along the process. Therefore, she presented higher initial scores and smaller changes at reevaluation.

Regarding the QUEST 2.0, to assess the satisfaction with the resource, the participants should consider the entire set of hardware (gBox, notebook computer, eye tracker, router, and portable table) and software (CI). Participants PP1 and PP3 scored less than 5.0, referring to difficulties in calibrating the eye tracker, occasional visual discomfort, and difficulty in using the system (in the case of the participant with the greatest motor impairment).

To assess the satisfaction with the service provided, the participants considered installation of the equipment, explanations, training, troubleshooting, and necessary follow-up during the week of use. In this item, all the scores were the highest (5.0). ~~Good~~ professionals and services are items appointed by Lenker et al. [22] as a ~~positive~~ point in the process of obtaining an AT resource, leading to better ~~results~~ with use. In contrast, lack of continuous support can lead participants to lose interest in its use [17].

The QUEST 2.0 total average (between 4.6 and 5.0) obtained in this research shows that the participants were satisfied with the SE. This result corroborates the findings of two studies of the systematic review conducted by Brandt et al. [48] on environmental control systems and smart homes used by people with disabilities.

The aspect that the participants considered most important in the SE control system was ‘effectiveness’. Demers et al. [72] defined this term as the “goal achievement with the AT device” (p.189), reinforcing that the system has met the needs of these people. Our findings corroborate those by Shone Stickel et al. [73], who also found effectiveness as the most important attribute of electronic AT devices for performance of ADL.

In the PIADS, respondents assessed how they were affected by the SE system. Participants PP1, PP5, and PP6 had the highest average values, indicating a maximum positive impact with the use of the SE. They assigned the highest values to the Adaptability subscale, indicating that with the use of the system they felt more willing to take risks and more motivated to participate socially [66]. Participant PP3, who has the most significant motor impairment, presented the lowest average among the participants. This instrument, as previously mentioned, was answered by her mother based on what she believed her daughter’s assessment would be. Thus, it may not reliably represent the participant's assessment.

The developers of this instrument [66] claim that these three subscales (Competence, Adaptability, and Self-esteem) are sufficiently sensitive to assess the psychosocial impact of an AT device or resource on the user, which are included in the QoL concept. In addition, the longer the period of use, the greater the feeling of competence [74], being that the hypotheses for it are that the longer the usage time: 1) the more the users appreciate the effect; 2) reflects the user's real need for the device.

Due to the short time of use of the SEs in this study (one week), it is not possible to state that there was a real change in the psychosocial aspects of the participants, but it indicates a tendency toward this change, in view of the results.

Regarding the SUS instrument, the result (85.6) indicates that the usability of our system was well evaluated. According to Bangor, Kortum and Miller [75], products evaluated in the range of 80 points are considered good, and products evaluated in the range of 90 points are considered exceptional.

The lowest evaluation refers, again, to participant PP3, whose answers pointed to some degree of complexity in the system and the need of a technical person or prior learning. Beyond the motor impairment of this participant, other factors may have interfered with the use of the eye tracker, such as her position in bed, small opening of the eye sometimes, ~~tiredness~~ with use, and difficulty of caregivers with the use of computers and programs.

Despite the lower ratings assigned by this participant, it seems that for all participants, on average, the assessment instruments showed positive results in relation to occupational performance, satisfaction with performance, satisfaction with the SE system, and usability of the system.

Many studies have evaluated improvements in these aspects after people with disabilities used environment control systems or electronic AT devices [17,44,73,76,77], whereas other studies have assessed ways of interacting with the environment through eye trackers [78,79]. However, no studies with the same objectives and using the same methodology of the present research, that is, use of IROG technology for SE control, have been found for comparison.

As the results show better occupational performance, satisfaction with performance ~~and~~ with the SE, and system usability, it can be concluded that the SE controlled by IROG

evaluated in this research provided people with motor disabilities with more independent operation and control of the equipment.

All the reports of participants point positive aspects with the use of the SE system. These statements corroborate the researched literature [80–83], since independence, control and privacy are highly important aspects pointed by people with disabilities who used environment control systems or electronic aids to daily living (EADL).

Participant PP6's speech also points to an outcome present in the study by Verdonck, Chard and Nolan's [81]: the embarrassment that people with disabilities present regarding their recurring requests for help, followed by apologies, as they feel uncomfortable to interrupt their caregivers' routine. According to those authors, the use of EADL changes this dynamic, with fewer apologies, less discomfort, and reduced caregiver burden.

The user manual was an additional material left with the participants to assist with the use of the SE system. The literature highlights how important explanations and training are for understanding the use and for adherence to the AT resource. Myburg et al. [71] found that training was considered crucial for the total integration of the environment control system in the lives of people with spinal cord injury, as well as the involvement of the occupational therapist in the testing, prescription and configuration of the system.

Information obtained through the Web application showed that the system was not used every day. The justifications given by the participants included trips, medical or rehabilitation consultations, and other leisure activities, such as going to church or taking short tours.

However, some other hypotheses were raised, corroborating the literature: the system has limitations, requiring other person to activate part of the equipment [82,84] or, when there is some voluntary movement, people prefer to behave as they are more accustomed

[85]. Another possibility is that the TV was controlled by a person who was in the same room as the participant, using the standard TV remote control.

About the participants who did not use the system, Costa et al. [85] found some factors that can contribute to the understanding: lack of equipment functionality (for not providing the desired independence), difficulty in use, embarrassment in using the device, lack of support from family members, lack of user motivation.

Regarding non-acceptance of diagnoses, studies have shown that this is an important factor to be considered when prescribing or selecting an AT device or resource [25,86]; however, in the present study, this information only appeared at the reevaluation.

Wessels et al. [25] reported that there is a difference in the way the AT resource will be viewed between people who were born with a disability (for them, technology opens a new range of possibilities) and those who have acquired a disability (because, for them, technology will never replace the lost function).

Participants PP2 and PP4 are in the second group, since they acquired the disability as adults. A recurring line in the interviews is that they were very active and independent in the past, and now they are dependent for practically all activities. For them, the disability has also brought other types of losses, such as moving from their hometowns, changing their standard of living, ending relationships, or losing jobs. Such cases often result in periods of depression [25]. In this sense, for these people, it is hypothesized that they would only benefit from technology if their dependency could be completely reversed.

Another associated factor that may have contributed to non-use of the SE is that the AT device can highlights the disability [19,23,86]. Verza et al. [86] found that 30.3% of the reasons for abandoning or not using an AT device are due to the patient's non-acceptance. For those authors, although the AT device is seen as a possibility to increase

functionality, it can be interpreted as a validation of the disability and loss of independence, resulting in decreased self-esteem.

It should be noted that, although the system registers activation of the equipment, this information was not passed on to the participants, so that the use of the system was based on their real needs and desires, and not on the fact that they felt obliged to use it.

It is worth restating that it is important that the professional involved perform a wide and in-depth assessment of the patient's real demands, expectations, and possibilities of the proposed AT device, as well as consider their participation in the choice. These points are important to ensure acceptance and continuity of use, since abandonment can represent a waste ~~of time and financial expenses~~ (their own or from the government) [17,24,26,27].

Conclusions

Participants who used the IROG-controlled SE system showed better occupational performance and satisfaction with performance. In addition, the psychosocial impact was close to the maximum, satisfaction with the system was well evaluated and, for three participants, the usability was considered good. The participant who had a more pronounced motor impairment and, therefore, more difficulty in using the system, rated its psychosocial impact and usability with lower scores.

The two participants who did not use the system presented characteristics such as non-acceptance of their diagnoses or current health conditions, weak relationship with caregivers, and need for assistance with using the equipment. Besides that, the fact that an AT device possibly reinforces disability may have corroborated these results.

The HAAT model was used as a basis for definition of the study methodology aiming to achieve its objectives. Although the AT resource (SE system) was previously defined, in order to be evaluated, the choice of participants was not random, and professionals were asked to indicate the possible participants. Regarding the Activity component, the activities considered important for each participant were evaluated according to their wishes, even though other possibilities are available for control. Still in the Activity component, it was considered the most familiar place to conduct the research, that is, each of the participant's homes.

Regarding the Human component, the motor and cognitive skills of the participant were taken into account. The eye tracker using IROG technology was choose as the most suitable technique, because is less intrusive. Motivation to resume performance of the activities was assessed through the COPM contemplating the most significant.

With respect to the Context component, it was noticed that the participants who had greater social support (from family members and/or formal caregivers) actually used the SE and were able to provide a more in-depth assessment. Those who did not have this support, or had it in a weakened way, did not use the SE.

Finally, regarding the Assistive Technology component, it was evaluated that the SE system functioned as a facilitator to carry out the desired activities, enabling greater exercise of autonomy and independence.

Concerning the ICF, its components can also be broken down into the elements of this research. Health Condition considered the severe motor disability of the participants. In Body Functions and Structures, the movement limitations interfered in the way each of them interacted with the electronic equipment. Activities important for the participants and their Participation were assessed through the FIM^{TM} and COPM instruments. Personal Factors were verified using the QUEST 2.0 and PIADS

instruments. Finally, about Environmental Factors, the SE system, as in the HAAT model, is considered a facilitator, and its usability was assessed through the SUS scale.

Considering that disability is indicated by the HAAT model and the ICF as inherent in social structures, and not in the person, the results found suggest that the SE system enabled reduction in the incapacity of the participants, who thus had greater participation in related activities.

This study provided a wide evaluation of equipment that aims to allow greater independence for people with severe motor disabilities, from the point of view of its operation and usability, as well as the benefits it provided to the people who used it. Several assessment instruments were used to achieve the study objectives.

For professionals, this study highlights the importance of a good evaluation for the prescription and development of AT resources, as well as new possibilities to provide people with severe motor disabilities with greater independence to carry out their everyday activities with regard to equipment control, avoiding abandonment or non-use.

Future studies with larger samples and longer duration should be conducted, expanding the possibilities of controlled equipment and devices, in order to understand whether the benefits remain in long term.

Patents

The gBox patent, together with the environment control system named “Remote micro-controlled device for charging residential loads via the Internet with emitter and receiver of commands via integrated infrared”, was submitted to the Institute of Technological Innovation – INIT at UFES, Brazil, and evaluation is under process.

Acknowledgments

The authors would like to thank all the professionals and participants involved in this study for their contribution.

References

1. Brasil. CORDE. Comitê de Ajudas Técnicas, ATA VII; 2008. Available from: <http://www.mj.gov.br/sedh/ct/corde/dpdh/corde/comite_at.asp>.
2. United States Congress. Assistive Technology Act of 2004: Amendment to the assistive technology act of 1998 (Public Law 108–364); 2004.
3. Siqueira SC, Vitorino PVO, Prudente COM, Santana TS, Melo GF. Quality of life of patients with Amyotrophic Lateral Sclerosis. Rev Rene. 2017; 18(1):139-146. doi: 10.15253/2175-6783.2017000100019.
4. Marrie RA, Cutter GR, Tyry T, Cofield SS, Fox R, Salter A. Upper limb impairment is associated with use of assistive devices and unemployment in multiple sclerosis. Multiple Sclerosis and Related Disorders. 2017; 13:87-92. doi: 10.1016/j.msard.2017.02.013.
5. Shamshiri H, Fatehi F, Abolfazli R, Harirchian MH, Sedighi B, Zamani B, et al. Trends of quality of life changes in amyotrophic lateral sclerosis patients. J Neurol Sci. 2016; 368:35-40. doi: 10.1016/j.jns.2016.06.056.
6. Bertoni R, Lamers I, Chen CC, Feys P, Cattaneo D. Unilateral and bilateral upper limb dysfunction at body functions, activity and participation levels in people with multiple sclerosis. Mult Scler. 2015; 21(12):1566-1574. doi: 10.1177/1352458514567553.
7. Souza FDA, Cruz DMC, Ferrigno ISV, Tsukimoto GR, Figliolia CS. Correlação entre papéis ocupacionais e independência de usuários com lesão medular em

processo de reabilitação. Mundo da Saúde. 2013; 37(2):166-175. doi: 10.15343/0104-7809.2013372166175.

8. Riazi A, Bradshaw SA, Playford ED. Quality of life in the care home: a qualitative study of the perspectives of residents with multiple sclerosis. Disabil Rehabil. 2012; 34(24):2095-2102. doi: 10.3109/09638288.2012.672539.
9. Lee YC, Chen YM, Hsueh IP, Wang YH, Hsieh CL. The impact of stroke: insights from patients in Taiwan. Occup Ther Int. 2010; 17(3):152-158. doi: 10.1002/oti.301.
10. Wolf TJ, Baum C, Connor LT. Changing face of stroke: Implications for occupational therapy practice. AJOT. 2009; 63:621-625. doi: 10.5014/ajot.63.5.621.
11. Pedretti LW, Early ME. Desempenho ocupacional e modelos de prática para disfunção física. In. Pedretti LW, Early ME. Terapia ocupacional: capacidades práticas para disfunções físicas. São Paulo: Roca; 2004.
12. Camacho A, Esteban J, Paradas C. Informe de la Fundación del Cerebro sobre el impacto social de la esclerosis lateral amiotrófica y las enfermedades neuromusculares. Neurología. 2015; 33:35-46. doi: 10.1016/j.nrl.2015.02.003.
13. World Health Organization – WHO. International Classification of Functioning, Disability and Health: ICF. World Health Organization; 2001.
14. Farias N, Buchalla CM. A Classificação Internacional de Funcionalidade, Incapacidade e Saúde da Organização Mundial da Saúde: conceitos, usos e perspectivas. Rev Bras Epidemiol. 2005; 8(2):187-193. doi: 10.1590/S1415-790X2005000200011.

15. Dahl TH. International classification of functioning, disability and health: an introduction and discussion of its potential impact on rehabilitation services and research. J Rehabil Med. 2002; 34:201-204. doi: 10.1080/165019702760279170.

16. Borg J, Lindström A, Larsson S. Assistive technology in developing countries: national and international responsibilities to implement the Convention on the Rights of Persons with Disabilities. Viewpoint. Lancet. 2009; 374(28):1863-1865. doi: 10.1016/S0140-6736(09)61872-9.

17. Jamwal R, Callaway L, Ackerl J, Farnworth L, Winkler D. Electronic assistive technology used by people with acquired brain injury in shared supported accommodation: Implications for occupational therapy. British Journal of Occupational Therapy. 2017; 80(2):89-98. doi: 10.1177/0308022616678634.

18. Cruz DMC, Ioshimoto MTA. Tecnologia assistiva para as atividades de vida diária na tetraplegia completa C6 pós-lesão medular. Rev Triang: Ens. Pesq. Ext. Uberaba. 2010; 3(2):177-190. doi: 10.18554/rt.v3i2.153.

19. Andrade VS, Pereira LSM. Influência da tecnologia assistiva no desempenho funcional e na qualidade de vida de idosos comunitários frágeis: uma revisão bibliográfica. Rev Bras Geriatr Gerontol. 2009; 12(1):113-122. doi: 10.1590/1809-9823.2009120110.

20. Plotkin A, Sela L, Weissbrod A, Kahana R, Haviv L, Yeshurun Y, et al. Sniffing enables communication and environmental control for the severely disabled. PNAS. 2010; 107(32):14413-14418. doi: 10.1073/pnas.1006746107.

21. Lulé D, Zickler C, Häcker S, Bruno MA, Demertzi A, Pellas F, et al. Life can be worth living in locked-in syndrome. Progress in Brain Research. 2009; 177:339-351. doi: 10.1016/S0079-6123(09)17723-3.

22. Lenker JA, Harris F, Taugher M, Smith RO. Consumer perspectives on assistive technology outcomes. Disabil Rehabil Assist Technol. 2013; 8(5):373-380. doi: 10.3109/17483107.2012.749429.

23. Squires LA, Williams N, Morrison VL. Matching and accepting assistive technology in multiple sclerosis: A focus group study with people with multiple sclerosis, carers and occupational therapists. Journal of Health Psychology. 2016; 24:1-15. doi: 10.1177/1359105316677293.

24. Cruz DMC, Emmel MLG, Manzini MG, Mendes PVB. Assistive technology accessibility and abandonment: challenges for occupational therapists. The Open Journal of Occupational Therapy. 2016; 4(1):Article 10. doi: 10.15453/2168-6408.1166.

25. Wessels R, Dijcks B, Soede M, Gelderblom GJ, De Witte L. Non-use of provided assistive technology devices: a literature overview. Technology and Disability. 2003; 15(4):231-238. doi: 10.3233/TAD-2003-15404.

26. Riemer-Reiss ML, Wacker RR. Factors associated with assistive technology discontinuance among individuals with disabilities. Journal of Rehabilitation. 2000; 66(3):44-50.

27. Phillips B, Zhao H. Predictors of assistive technology abandonment. Assistive Technology. 1993; 5(1):36-45. doi: 10.1080/10400435.1993.10132205.

28. Alper S, Raharinirina S. Assistive technology for individuals with disabilities: a review and synthesis of the literature. Journal of Special Education Technology. 2006; 21(2):47-64. doi: 10.1177/016264340602100204.

29. Cook A, Polgar J. Assistive Technologies: Principles and Practice. 4th ed. St. Louis: Mosby Elselvier; 2015.

30. Kinney A, Goodwin DM, Gitlow L. Measuring assistive technology outcomes: a user centered approach. Assistive Technology Outcomes and Benefits. 2016; 10:94-110.

31. Peterson DB, Murray GC. Ethics and assistive technology service provision. Disabil Rehabil Assist Technol. 2006; 1:59-67. doi: 10.1080/09638280500167241.

32. Rocha EF, Castiglioni MC. Reflexões sobre recursos tecnológicos: ajudas técnicas, tecnologia assistiva, tecnologia de assistência e tecnologia de apoio. Rev Ter Ocup Univ São Paulo. 2005; 16(3):97-104. doi: 10.11606/issn.2238-6149.v16i3p97-104.

33. Netten JJ, Dijkstra PU, Geertzen JHB, Postema K. What influences a patient's decision to use custom-made orthopaedic shoes? BMC Musculoskeletal Disorders. 2012; 13:92. doi: 10.1186/1471-2474-13-92.

34. Rampinelli M, Bastos TF, Vassallo RF, Pizarro D. Implementation of an intelligent space for localizing and controlling a robotic wheelchair. In: 2012 ISSNIP Biosignals and Biorobotics Conference: Biosignals and Robotics for Better and Safer Living (BRC); 9-11th January 2012; Manaus-Brazil: IEEE – Institute of Electrical and Electronics Engineers; 2012. 1-4. doi: 10.1109/BRC.2012.6222187.

35. Lee JH, Hashimoto H. Intelligent Space: concept and contents. Advanced Robotics. 2002; 16(3):265-280. doi: 10.1163/156855302760121936.

36. Aarts EH, Encarnação JL. Into Ambient Intelligence. In. Aarts EH, Encarnação JL, editors. True visions: The emergence of ambient intelligence. Springer: Netherlands; 2008. pp. 1-16.

37. Kadam R, Mahamuni P, Parikh Y. Smart Home System. International Journal of Innovative Research in Advanced Engineering. 2015; 2:81-86.

38. Rivera-Illingworth F, Callaghan V, Hagras H. A neural network agent based approach to activity detection in AmI environments. In: Proceedings of IEEE

International Workshop on Intelligent Environments; 28-29th June 2005; Colchester (United Kingdom). Institution of Engineering and Technology; 2005. 1-12. doi: 10.1049/ic:20050222.

39. Tello RJMG, Bissoli ALC, Ferrara F, Müller S, Ferreira A, Bastos-Filho TF. Development of a Human Machine Interface for Control of Robotic Wheelchair and Smart Environment. IFAC-PapersOnLine. 2015; 48:136-141. doi: 10.1016/j.ifacol.2015.12.023.

40. Hussein A, Adda M, Atieh M, Fahs W. Smart Home Design for Disabled People based on Neural Networks. Procedia Computer Science. 2014; 37:117-126. doi: 10.1016/j.procs.2014.08.020.

41. Rampinelli M, Covre VB, Queiroz FM, Vassallo RF, Bastos-Filho TF, Mazo M. An intelligent space for mobile robot localization using a multi-camera system. Sensors. 2014; 14:15039-15064. doi: 10.3390/s140815039.

42. Adami I, Antona M, Stephanidis C. Ambient Assisted Living for the Motor Impaired. In. Koroupetroglou G, editor. Assistive Technologies, Disability Informatics and Computer Access for Motor Limitations. USA: IGI Global; 2013, pp. 76-104.

43. Bastos Filho TF, Fernandes MR, Lucena-Jr VF, Pereira CE. Proposal of Architecture for Integration of a Wheelchair in an Intelligent Space. In: Proceedings of 4th IEEE Biosignals and Biorobotics Conference (ISSNIP); 18-20th February 2013; Rio de Janeiro-RJ (Brazil); 2013. 6-11.

44. Ocepek J, Roberts AEK, Vidmar G. Evaluation of treatment in the smart home IRIS in terms of functional independence and occupational performance and satisfaction. Computational and Mathematical Methods in Medicine. 2013; 2013:10 pages. doi: 10.1155/2013/926858.

45. Gentry T. Smart homes for people with neurological disability: State of the art. NeuroRehabilitation. 2009; 25:209-217. doi: 10.3233/NRE-2009-0517.

46. Muñoz D, Fortes P. O princípio da autonomia e o consentimento livre e esclarecido. In. Iniciação à Bioética. Costa SIF, Oselka G, Garrafa V, coordinators. Brasília: CFM; 1998. pp. 53-70.

47. Martin S, Kelly G, Kernohan WG, McCreight B, Nugent C. Smart home technologies for health and social support. Cochrane Database of Systematic Reviews. 2008; 4:1-10. doi: 10.1002/14651858.CD006412.pub2.

48. Brandt Å, Samuelsson K, Töytäri O, Salminen A-L. Activity and participation, quality of life and user satisfaction outcomes of environmental control systems and smart home technology: a systematic review. Disability and Rehabilitation: Assistive Technology. 2011; 6(3):189-206. doi: 10.3109/17483107.2010.532286.

49. Marikyan D, Papagiannidis S, Alamanos E. A systematic review of the smart home literature: A user perspective. Technological Forecasting & Social Change. 2019; 138:139-154. doi: 10.1016/j.techfore.2018.08.015.

50. Bissoli A, Lavino-Junior D, Sime M, Encarnação L, Bastos-Filho T. A Human-Machine Interface Based on Eye Tracking for Controlling and Monitoring a Smart Home Using the Internet of Things. Sensors. 2019; 19:859-884. doi: 10.3390/s19040859.

51. Ding Z, Luo J, Deng H. Accelerated exhaustive eye glints localization method for infrared video oculography. In: SAC '18 – Proceedings of the 33rd Annual ACM Symposium on Applied Computing; April 2018; Pau France. New York-NY (United States of America): Association for Computing Machinery; 2018. 620–627. doi: https://doi.org/10.1145/3167132.3167200.

52. Eid MA, Giakoumidis N, El-Saddik A. A Novel Eye-Gaze-Controlled Wheelchair System for Navigating Unknown Environments: Case Study with a Person with ALS. IEEE Access. 2016; 4:558-573. doi:10.1109/ACCESS.2016.2520093.
53. Giannotto EC. Uso de rastreamento do olhar na avaliação da experiência do tele-usuário de aplicações de TV interativa. M.Sc. Thesis, Polytechnic School of the University of São Paulo. Department of Computer Engineering and Digital Systems. 2009, 290 p. Available from: https://teses.usp.br/teses/disponiveis/3/3141/tde-15042009-151212/pt-br.php.
54. Duchowski AT. Eye tracking methodology: Theory and practice. 2nd ed. London: Springer-Verlag; 2007.
55. Anders S, Weiskopf N, Lule D, Birbaumer N. Infrared oculography—validation of a new method to monitor startle eyeblink amplitudes during fMRI. NeuroImage. 2004; 22(2):767-770. doi: 10.1016/j.neuroimage.2004.01.024.
56. Sharma A, Abrol P. Eye gaze techniques for human computer interaction: a research survey. International Journal of Computer Applications. 2013; 71(9):18-29. doi: 10.5120/12386-8738.
57. Van Es MA, Hardiman O, Chio A, Al-Chalabi A, Pasterkamp RJ, Veldink JH, et al. Amyotrophic lateral sclerosis. The Lancet. 2017; 390(10107):2084-2098. doi: 10.1016/S0140-6736(17)31287-4.
58. Alves ACJ, Matsukura TS. Revisão sobre avaliações para indicação de dispositivos de tecnologia assistiva. Rev Ter Ocup Univ São Paulo. 2014; 25(2):199-207. doi: 10.11606/issn.2238-6149.v25i2p199-207.
59. Lovarini M, Mccluskey A, Curtin M. Editorial: Critically appraised papers. Limited high-quality research in the effectiveness of assistive technology. Aust Occup Ther J. 2006; 50(1):50.

60. Gelderblom GJ, De Witte LP. The assessment of assistive technology outcomes, effects and costs. Technol and Disabil. 2002; 14(3):91-94. doi: 10.3233/tad-2002-14302.

61. Tobii Eye Tracker 4C. Available from: https://gaming.tobii.com/product/tobii-eye-tracker-4c/. Access in: February 24, 2019.

62. Tobii Dynavox. Gaze Point. Available from: https://www.tobiidynavox.com/en-us/software/free-resources/gaze-point-1/. Acess in: February 24, 2019.

63. Riberto M, Miyazaki MH, Jucá SSH, Sakamoto H, Pinto PPN, Battistella LR. Validação da Versão Brasileira da Medida de Independência Funcional. Acta Fisiatr. 2004; 11(2):72-76. doi: 10.5935/0104-7795.20040003.

64. Linacre JM, Heinemann AW, Wright BD, Granger CV, Hamilton BB. The structure and stability of the Functional Independence Measure. Arch Phys Med Rahabil. 1994; 75:127-132. doi: 10.1016/0003-9993(94)90384-0.

65. Law M, Baptiste S, Carswell A, McColl M, Polatajko H, Pollock N. Medida canadense de desempenho ocupacional (COPM). Magalhães LC, Magalhães, LV, Cardoso AA, organizers and translators. Belo Horizonte: Ed UFMG; 2009.

66. Jutai J, Day H. Psychosocial Impact of Assistive Devices Scale (PIADS). Technol and Disabil. 2002; 14:107-111. doi: 10.1037/t45599-000.

67. Demers L, Weiss-Lambrou R, Ska B. The Quebec User Evaluation of Satisfaction with Assistive Technology (QUEST 2.0): an overview and recent progress. Technol and Disabil. 2002; 14:101-105. doi: 10.13072/midss.298.

68. Carvalho KEC, Gois Junior MB, Sá KN. Tradução e validação do Quebec User Evaluation of Satisfaction with Assistive Technology (QUEST 2.0) para o idioma português do Brasil. Rev. Bras. Reumatol. 2014; 54(4):260-267. doi: 10.1016/j.rbr.2014.04.003.

69. Brooke J. SUS: a "quick and dirty" usability scale. In: Jordan PW, Thomas B, McClelland IL, Weerdmeester B, editors. Usability Evaluation in Industry. London: Taylor and Francis; 1996. 189-194.

70. Calvo A, Chiò A, Castellina E, Corno F, Farinetti L, Ghiglione P, et al. Eye tracking impact on Quality-of-life of ALS patients. In: Computers Helping People with Special Needs. Proceedings of 11th International conference on computers helping people with special needs; 9-11th July 2008; Linz (Austria): Springer-Verlag Berlin Heidelberg; 2008. 70-77. doi: 10.1007/978-3-540-70540-6.

71. Myburg M, Allan E, Nalder E, Schuurs S, Amsters D. Environmental control systems – the experiences of people with spinal cord injury and the implications for prescribers. Disability and Rehabilitation: Assistive Technology. 2017; 12(2):128-136. doi: 10.3109/17483107.2015.1099748.

72. Demers L, Wessels R, Weiss-Lambrou R, Ska B, de Witte LP. Key dimensions of client satisfaction with assistive technology: a cross-validation of a Canadian measure in the Netherlands. J Rehabil Med. 2001; 33:187-191. doi: 10.1080/165019701750300663.

73. Shone SM, Ryan S, Rigby PJ, Jutai JW. Toward a comprehensive evaluation of the impact of electronic aids to daily living: evaluation of consumer satisfaction. Disabil Rehabil. 2002; 24:115-125. doi: 10.1080/09638280110066794.

74. Day H, Jutai J. Measuring the psychosocial impact of assistive devices: the PIADS. Canadian Journal of Rehabilitation. 1996; 9(2):159-168.

75. Bangor A, Kortum P, Miller JA. The System Usability Scale (SUS): An Empirical Evaluation. International Journal of Human-Computer Interaction. 2008; 24(6):574-594. doi: 10.1080/10447310802205776.

76. Little R. Electronic aids for daily living. Phys Med Rehabil Clin N Am. 2010; 21:33-42. doi: 10.1016/j.pmr.2009.07.008.

77. Boman I-L, Tham K, Granqvist A, Bartfai A, Hemmingsson H. Using electronic aids to daily living after acquired brain injury: A study of the learning process and the usability. Disability and Rehabilitation: Assistive Technology. 2007; 2(1):23-33. doi: 10.1080/17483100600856213.

78. Boustany G, Itani AED, Youssef R, Chami O, Abu-Faraj ZO. Design and development of a rehabilitative eye-tracking based home automation system. In: Proceedings of 2016 3rd Middle East Conference on Biomedical Engineering (MECBME); 6-7th October 2016; Beirut (Lebanon). Institute of Electrical and Electronics Engineers (IEEE): Curran Associates, Inc.; Jan 2017. 30-33. doi: 10.1109/MECBME.2016.7745401.

79. Brennan CP, McCullagh PJ, Galway L, Lightbody G. Promoting autonomy in a smart home environment with a smarter interface. In: Proceedings of 2015 37th Annual International Conference of the IEEE Engineering in Medicine and Biology Society (EMBC); August 25-29 2015; MiCo – Milano Conference Center, Milan (Italy). Institute of Electrical and Electronics Engineers (IEEE): Curran Associates, Inc.; Dec 2015. 5032-5035. doi: 10.1109/EMBC.2015.7319522.

80. Hooper B, Verdonck M, Amsters D, Myburg M, Allan E. Smart-device environmental control systems: experiences of people with cervical spinal cord injuries. Disability and Rehabilitation: Assistive Technology. 2018; 13(8):24-730. doi: 10.1080/17483107.2017.1369591.

81. Verdonck MC, Chard G, Nolan M. Electronic aids to daily living: be able to do what you want. Disability and Rehabilitation: Assistive Technology. 2010; 6(3):268-281. doi:10.3109/17483107.2010.525291.

82. Judge S, Robertson Z, Hawley MS. Users' Perceptions of Environmental Control Systems. In: Emiliani PL, Burzagli L, Como A, Gabbanini F, Salminen A-L (eds.). Assistive Technology from Adapted Equipment to Inclusive Environments - AAATE 2009; August 31st–September 2nd 2009; Florence (Italy). Amsterdam: IOS Press; 2009. 426-431.

83. Harmer J, Bakheit AMO. The benefits of environmental control systems as perceived by disabled users and their carers. British Journal of Occupational Therapy. 1999; 62(9):394-398. doi: 10.1177/030802269906200902.

84. Palmer P, Seale J. Exploring the attitudes to environmental control systems of people with physical disabilities: a grounded theory approach. Technol Disabil. 2007; 9(1):17-27. doi: 10.3233/TAD-2007-19103.

85. Costa CR, Ferreira FMRM, Bortolus MV, Carvalho MGR. Dispositivos de tecnologia assistiva: fatores relacionados ao abandono. Cad. Ter. Ocup. UFSCar. 2015; 23(3):611-624. doi: 10.4322/0104-4931.ctoAR0544.7.8.

86. Verza R, Carvalho ML, Battaglia MA, Uccelli MM. An interdisciplinary approach to evaluating the need for assistive technology reduces equipment abandonment. Mult Scler. 2006; 12(1):88-93. doi: 10.1191/1352458506ms1233oa.

Response to Reviewers

Title: Usability, occupational performance and satisfaction evaluation of a smart environment controlled by infrared oculography by people with severe motor disabilities

In response to the reported pending issues, the following information is required:

- **Point 1**: The paper needs to be substantially shortened and condensed.

Answer to point 1: As recommended by the reviewer, the introduction and review have been combined and condensed. The description of the materials and instruments has been reduced. The results and discussion were separated and reorganized.

- **Point 2**: Details and photos that might allow to identify the participants should be omitted.

Answer to point 2: The vignettes with the details and photos of the participants have been removed and only the sociodemographic information that contributed to the discussion was maintained.

- **Point 3**: About the funding received of the Google Inc.

Answer to point 3: Google Inc. only supported the research through Google's Latin America Research Awards to two authors, Teodiano Freire Bastos-Filho and Alexandre Luís Cardoso Bissoli, and this does not alter our adherence to Plos ONE policies on sharing data and materials. This statement can also be found in the cover letter, in the Competing Interests Statement section, as recommended

- **Point 4**: Please explain in more detail why "data cannot be shared publicly because of Ethics Committee terms".

Answer to point 4: Our data contained information that identified the participants. This information has been omitted and the data are being shared in the Supporting Information files.

- **Point 5**: Please amend the methods section and ethics statement of the manuscript to explicitly state that the patient/participant has provided consent for publication: “The individual in this manuscript has given written informed consent (as outlined in PLOS consent form) to publish these case details”.

Answer to point 5: About the use of personal data, participants or their guardians signed the Free and Informed Consent Form (FICF), which guarantees that all personal data are confidential and private, even after the publication of the research. The data protection has been guaranteed by identifying participants by codes, instead of their names or initials, and by not including information such as date of birth and address. Detailed information about the participants health condition had already been excluded from the manuscript after the original decision letter. The terms with the highlighted subsection in both the original version in Portuguese and the translated version in English were included in the list of documents for submission. The current situation in Brazil, due to the COVID-19 pandemic is very serious, and it has been very difficult to meet the participants to obtain their signature on the PLOS specific consent form. Since they are people with disabilities and comorbidities, they have been in quarantine, as a measure to prevent spread of the Sars-CoV-2 virus.

The methods section and ethics statement of the manuscript were amended to explicitly state that the participants has provided consent for publication, through the FICF.

Sincerely,

Mariana Midori Sime (e-mail: mariana.sime@ufes.br)

Vitória-ES (Brazil), May 01, 2021.
