# Supplementary material for: Usability, occupational performance and satisfaction evaluation of a smart environment controlled by infrared oculography by people with severe motor disabilities
Source: PLoS One. 2021 Aug 13;16(8):e0256062. doi: 10.1371/journal.pone.0256062 (PMC8362986; doi:10.1371/journal.pone.0256062)
Supplement: Attachment — Submitted filename: Response to Reviewers.docx [file pone.0256062.s003.docx]

**Response to Reviewers**

**Title**: Usability, occupational performance and satisfaction evaluation of a smart environment controlled by infrared oculography by people with severe motor disabilities

In response to the reported pending issues, the following information is required:

- **Point 1**: The paper needs to be substantially shortened and condensed.

Answer to point 1: As recommended by the reviewer, the introduction and review have been combined and condensed. The description of the materials and instruments has been reduced. The results and discussion were separated and reorganized.

- **Point 2**: Details and photos that might allow to identify the participants should be omitted.

Answer to point 2: The vignettes with the details and photos of the participants have been removed and only the sociodemographic information that contributed to the discussion was maintained.

- **Point 3**: About the funding received of the Google Inc.

Answer to point 3: Google Inc. only supported the research through Google’s Latin America Research Awards to two authors, Teodiano Freire Bastos-Filho and Alexandre Luís Cardoso Bissoli, and this does not alter our adherence to Plos ONE policies on sharing data and materials. This statement can also be found in the cover letter, in the Competing Interests Statement section, as recommended

- **Point 4**: Please explain in more detail why "data cannot be shared publicly because of Ethics Committee terms".

Answer to point 4: Our data contained information that identified the participants. This information has been omitted and the data are being shared in the Supporting Information files.

- **Point 5**: Please amend the methods section and ethics statement of the manuscript to explicitly state that the patient/participant has provided consent for publication: “The individual in this manuscript has given written informed consent (as outlined in PLOS consent form) to publish these case details”.

Answer to point 5: About the use of personal data, participants or their guardians signed the Free and Informed Consent Form (FICF), which guarantees that all personal data are confidential and private, even after the publication of the research. The data protection has been guaranteed by identifying participants by codes, instead of their names or initials, and by not including information such as date of birth and address. Detailed information about the participants health condition had already been excluded from the manuscript after the original decision letter. The terms with the highlighted subsection in both the original version in Portuguese and the translated version in English were included in the list of documents for submission. The current situation in Brazil, due to the COVID-19 pandemic is very serious, and it has been very difficult to meet the participants to obtain their signature on the PLOS specific consent form. Since they are people with disabilities and comorbidities, they have been in quarantine, as a measure to prevent spread of the Sars-CoV-2 virus.

The methods section and ethics statement of the manuscript were amended to explicitly state that the participants has provided consent for publication, through the FICF.

Sincerely,

Mariana Midori Sime (e-mail: mariana.sime@ufes.br)

Vitória-ES (Brazil), May 01, 2021.
