# Supplementary material for: Usability, occupational performance and satisfaction evaluation of a smart environment controlled by infrared oculography by people with severe motor disabilities
Source: PLoS One. 2021 Aug 13;16(8):e0256062. doi: 10.1371/journal.pone.0256062 (PMC8362986; doi:10.1371/journal.pone.0256062)
Supplement: Attachment — Submitted filename: Response to Reviewers.docx [file pone.0256062.s005.docx]

Vitória, Brazil, 25 July 2021

**Response to Reviewers**

**Title**: Usability, occupational performance and satisfaction evaluation of a smart environment controlled by infrared oculography by people with severe motor disabilities

In response to the reported pending issues, the following information is required:

- **Point 1**: The conclusion could be condensed further.

Answer to point 1: The Conclusions section has been shortened, as solicited.

- **Point 2**: It is necessary to add a paragraph where the potential limitations of the study are discussed.

Answer to point 2: A paragraph describing the limitations of the study resulting from its small sample size has been included in the Conclusions section.

- **Point 3**: Explain how the FIM^TM^ was administered.

Answer to point 3: Regarding the questioning of the reviewer about the application of the FIM^TM^, it has been included in the text that the instrument was applied by a certified researcher through interviews, as validated in Brazil. Validation of the FIM^TM^ in Brazil was performed by Riberto et al. (2004), which is reference no. 63 in the paper.

Sincerely,

Mariana Midori Sime (e-mail: mariana.sime@ufes.br)
